# Diverse biosynthetic pathways in Arctic hydrothermal biofilms

**DOI:** 10.1038/s41467-026-77384-5

**Published:** 2026-09-03

**Authors:** Thuc Trong Nguyen, Ida Helene Steen, Runar Stokke

**Affiliations:** Department of Biological Sciences and Centre for Deep Sea Research, University of Bergen, Bergen, Norway

**Keywords:** Water microbiology, Environmental microbiology

## Abstract

Biosynthetic gene clusters (BGCs) which encode diverse secondary metabolites are ubiquitous across microbiomes and support critical ecological functions. They also serve as attractive targets for new drug discovery. Here we combined genome-resolved metagenomics with long-read Nanopore RNA sequencing, yielding 1016 bacterial and 124 archaeal medium-to-high quality MAGs from previously unchartered microbial communities in Arctic hydrothermal vent biofilms. We identified 2965 BGCs from 870 metagenome-assembled genomes (MAGs) comprising a distinctive and rich diversity of BGCs, with ribosomally synthesized and post-translationally modified peptides (RiPPs) predominating across all samples. RiPPs and non-ribosomal peptide synthetases (NRPs), also known to encode metabolites with antimicrobial potential, are represented among the most expressed transcripts. Terpenes, though less expressed, contribute to microbial signaling and defense. Notably, we identify hydrogen cyanide (HCN) synthesis pathways in archaeal genomes, challenging the view that cyanogenesis is restricted to bacteria and eukaryotes. Our findings demonstrate that microbial adaptation to extreme environments favors RiPP-based biosynthesis and that HCN may play a role in archaeal ecological interactions. Moreover, microbial communities in Arctic hydrothermal vent biofilms provide a rich reservoir of unique bioactive compounds, with implications for drug discovery.

## Abstract (web summary)

Biosynthetic genes produced from microbes have unique industrial properties, however, the diversity of such genes from extreme environments remains limited. Here, the authors use meta-omics of Arctic hydrothermal vent biofilms and uncover nearly 3000 genes, including a possibility for hydrogen cyanide production in archaea.

## Introduction

Antibiotic resistance is a growing global crisis, responsible for over 1.27 million deaths annually as of 2019^1^. This situation raises the need to find new antibiotics, particularly those derived from natural products, as many approved antibiotics have natural origins^1–3^. These bioactive compounds are typically synthesized by BGCs, which are physically co-located sets of genes encoding the enzymes, transporters, and regulatory elements required for secondary metabolite biosynthesis^4^. BGCs play important ecological roles by mediating chemical interactions within microbial communities^5^. Their products, known as secondary metabolites, can function as antimicrobials, toxins, siderophores, or signaling molecules, facilitating competition, cooperation, or adaptation in diverse environments^5^. BGCs are recognized as an important source of clinically valuable natural products, such as antibiotics (e.g., streptomycin, erythromycin), anticancer agents (e.g., doxorubicin), and immunosuppressants (e.g., rapamycin)^3^. Due to their essential biological activities, BGC-derived metabolites remain central to drug discovery efforts^6,7^. In response to rising antibiotic resistance, genome mining of uncultivated microorganisms, particularly those from underexplored environments, is increasingly employed to identify novel BGCs with therapeutic potential^6,8^.

Deep-sea hydrothermal vent ecosystems represent one of the most extreme and dynamic habitats on Earth, where primary production is predominantly fueled by chemosynthesis rather than photosynthesis^9^. Since the first discovery in the late 1970s^10^, a vast microbial diversity has been discovered on a global scale^11–13^. The use of single-cell and genome-resolved metagenomics has been a driver for microbial discoveries in hydrothermal and subseafloor environments^14–25^. Coupled with improved analytical and computational tools, this has led to the discovery of novel metabolisms, novel microbial lineages, as well as known metabolisms in new lineages^26–29^. Prokaryotic BGCs from various marine environments, including water columns, deep-sea sediments, cold-seep sediments, and bacterial mats, have been shown to encode a wide array of novel secondary metabolites, many with potential antimicrobial properties^21,30–34^. In contrast, the microbial communities inhabiting deep-sea hydrothermal vent biofilms remain largely unexplored in this context, despite their biodiversity and potential to yield previously unrecognized biosynthetic pathways.

Biofilm-forming microorganisms play important ecological roles in vent ecosystems by structuring microbial assemblages and mediating nutrient flux^35^. Given the spatial and resource limitations within biofilms, microbial interactions in marine biofilms occur through various mechanisms, including cooperation, communication, and competition^36^. The production and secretion of secondary metabolites, such as toxins and antibiotics, are common strategies employed by microbes to compete within biofilm communities^36,37^. Despite this, deep-sea hydrothermal vent biofilms have been largely overlooked as a source of secondary metabolites, and to date, BGCs in biofilm communities from Arctic Mid-Ocean Ridges (AMOR) hydrothermal systems have not been systematically described.

In this study, we explore the biodiversity and biosynthetic potential of biofilm microbial communities from the Loki’s Castle, Ægir Vent Field, and Seven Sisters vent field along the AMOR. By integrating Nanopore long-read metatranscriptomic data with BGC predictions from MAGs, we assess the transcriptional activity and site-specific expression profiles of BGCs across distinct vent ecosystems. Biofilm communities were taxonomically diverse, with bacteria dominating across all samples. Common deep-sea hydrothermal vent phyla such as *Pseudomonadota*, *Bacteriota*, *Aquificota*, and *Campylobacteota* were consistently present^21^. RiPPs represented the most prevalent BGC class, both at the genomic and transcriptomic levels, and were detected in both bacterial and archaeal lineages. Comparative analysis with the Minimum Information about a Biosynthetic Gene Cluster database (MIBiG)^4^ revealed that a large proportion of predicted BGCs do not cluster with any known reference, underscoring the uniqueness of hydrothermal vent biofilms as rich reservoirs of previously uncharacterized secondary metabolites. Notably, long-read transcriptomics data confirmed that many of these BGCs are actively expressed in situ, providing direct evidence that microbes within these extreme environments produce these secondary metabolites as part of their ecological strategy. This is the first comprehensive report on the microbial communities and biosynthetic capabilities of the Ægir vent field, and the first observation of the potential for HCN synthesis in the domain Archaea, specifically from an *Asgardarchaeota*-affiliated MAG. Together, our findings reveal the ecological importance of secondary metabolism in hydrothermal vent biofilms and highlight their untapped potential for natural product discovery.

## Results

### Microbial composition across biofilm communities

**Fig. 1:**
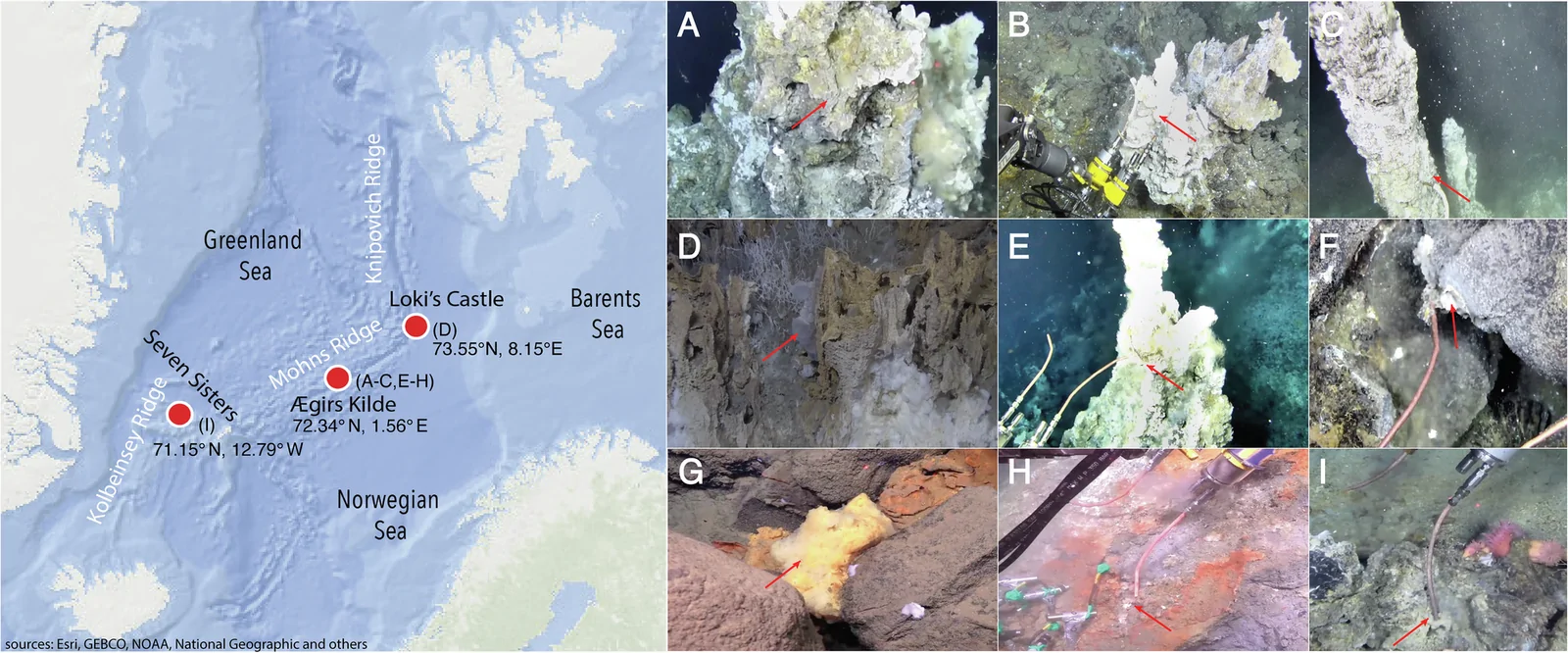
Sampling locations and biofilm collection across Arctic mid-ocean ridge hydrothermal vent fields. Sampling locations shown in the map. **A**–**C** and **E**–**H** intra-vent field sampling of biofilms from the Ægir Vent Field (Table 1; Metagenome ID M24, M25, M26, M36, M37, M38 and M39), **D** the Barite Field within the Loki’s Castle Vent Field (Table 1; Metagenome ID M33), and **I** the Seven Sisters Vent Field (Table 1; Metagenome ID M42). Sampled biofilms using a hydraulic biosyringe attached to the ROV arm are indicated by a red arrow. Samples were collected during four sampling campaigns by AMOR on board the research vessel G.O. SARS (Summer of 2017, 2020, 2021, and 2022). The map image is the intellectual property of Esri and is used herein under license. Copyright © 2026 Esri and its licensors. All rights reserved.

To resolve the microbial diversity and biosynthetic capacity of Arctic hydrothermal vent biofilms, we performed an integrated metagenome- and metatranscriptome-based study of nine unique hydrothermal biofilms (Fig.1, Table 1). The biofilms were collected from three distinct hydrothermal vent fields along AMOR, the shallow-water Seven Sisters vent field, the deep-sea Ægir vent field, and the deep-sea Loki’s Castle vent field. From the biofilms, we recovered 1147 MAGs. In addition, long-read nanopore metatranscriptomic data were obtained from seven of the biofilms. Sequencing information is given in Supplementary Data 1 and Supplementary Data 2.

**Table 1:** Samples and collection sites used in the study

Sample ID	Vent field	Metagenome ID	Metatranscriptome ID	Depth (m)	BioProject
GS21-ROV-106-ROV432	Ægir Vent Field	M24	c24	2288	PRJNA1301805
GS20-ROV9-BS1	Ægir Vent Field	M25	c25	2288	PRJNA881934
GS20-ROV9-BS2	Ægir Vent Field	M26	NA	2288	PRJNA1301797
GS17-BB	Loki’s Castle Vent Field (Barite field)	M33	c33	2390	PRJNA1301837
GS22-107-ROV569-BS1	Ægir Vent Field	M36	c36	2306	PRJNA1301809
GS22-107-ROV569-BS2	Ægir Vent Field	M37	c37	2308	PRJNA1301812
GS22-107-ROV579-BS1	Ægir Vent Field	M38	c38	2304	PRJNA1301820
GS22-107-ROV579-BS2	Ægir Vent Field	M39	c39	2317	PRJNA1301824
GS21-106-ROV435A-BS	Seven Sisters Vent Field	M42	NA	165	PRJNA1301830

**Fig. 2:**
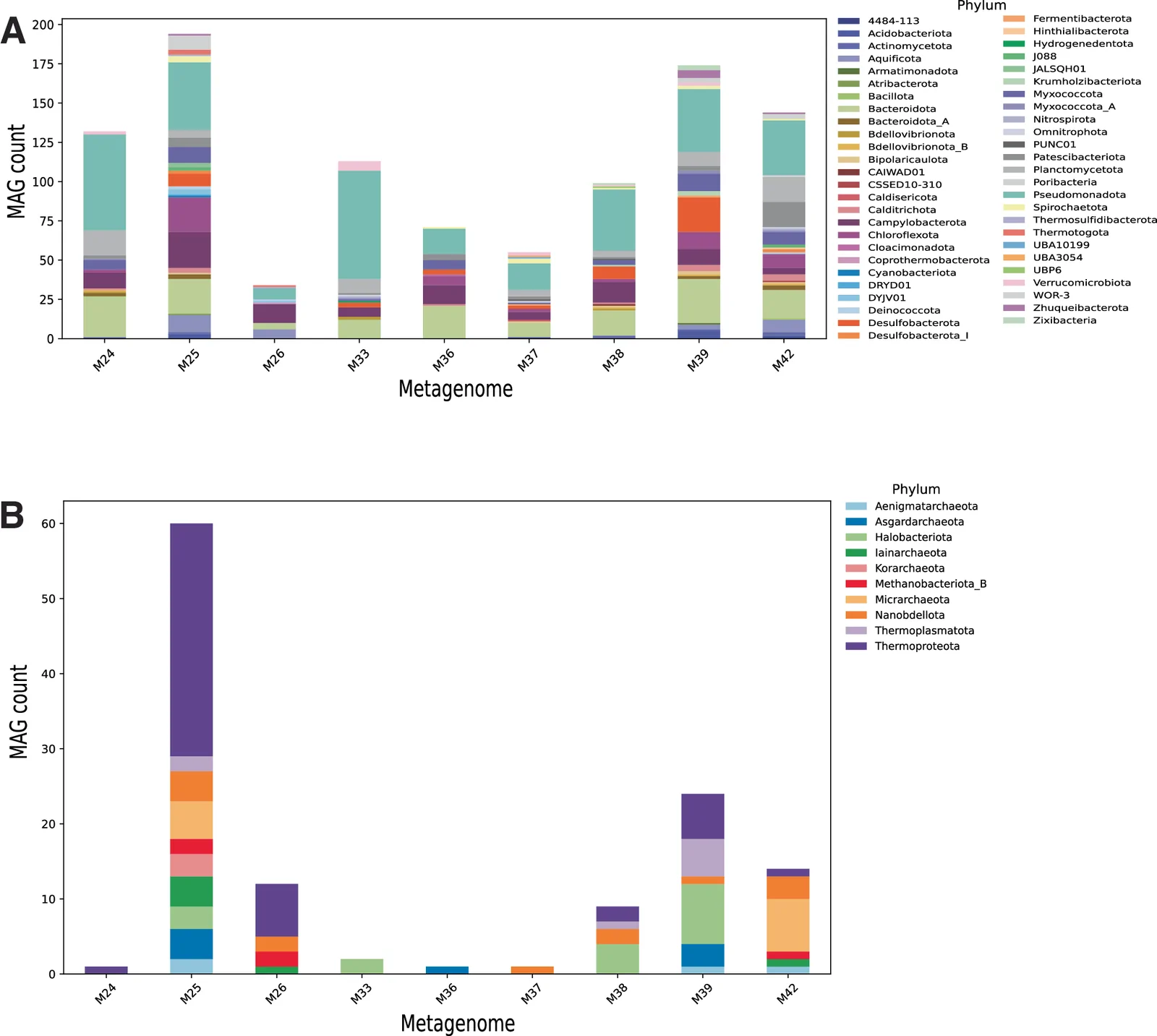
Taxonomic composition of bacterial and archaeal MAGs in AMOR biofilms. The number of MAGs identified in each of the nine biofilms, grouped by phylum within: **A** Bacteria; **B** Archaea. Taxonomic classifications are based on the Genome Taxonomy Database (GTDB v. R226).

After de-replication, 1140 medium-to-high quality bacterial and archaeal MAGs (minimum contig size >2000 bp) were retained (Supplementary Data 3). Among them, 124 were classified using GTDB-tk with database version R226 into 10 archaeal phyla, while 1016 MAGs belonged to 51 bacterial phyla. Within bacteria, *Pseudomonadota* was the most prevalent phylum, detected in all samples (327 MAGs), followed by *Bacteroidota* (156) (Fig. 2A). *Thermoproteota* was the dominant archaeal phylum, present in six of the nine metagenomes (Fig. 2B). Detailed taxonomic information is provided in the supplementary materials (Supplementary Data 3). Sample M25 exhibited the highest biodiversity, containing 254 MAGs from 27 bacterial and 10 archaeal phyla (Fig. 2A, B). Bacterial MAGs dominated all samples, but archaeal abundance was noticeably high in M25 and M39, with 60 and 24 archaeal MAGs, respectively. The archaeal taxa present suggest an elevated temperature in these samples, supported by the measured sediment temperature using a live temperature probe connected to the remotely operated vehicle (ROV). Just below the biofilm in M39, the temperature was measured at 33 °C, and at 10 cm sediment depth, the temperature was elevated to 96 °C. The recently described phylum JALSQH01 from deep-sea hydrothermal deposits^21^ was classified into three MAGs from sample M25. This suggests that this new taxon is more widely distributed and specific to the hydrothermal vent ecosystem.

Relative abundance analysis based on mapped reads to de-replicated MAGs using CoverM revealed that *Pseudomonadota* dominated seven of the nine biofilm communities, while *Campylobacterota* prevailed in samples M26 and M36. Notably, *Aquificota* demonstrates significant relative abundance in samples M25 (3.53%), M26 (11.85%), and M42 (10.56%). *Bacteroidota* is also an important bacterial phylum within the biofilm communities, contributing >1% to the total relative abundance in all samples except M26. Overall, between 20 and 75.3% of the sequenced microbial community was represented by the MAGs in the study. Detailed information on the relative abundance of each phylum is provided in the supplementary materials (Supplementary Data 3). The presence of well-known deep-sea groups such as *Pseudomonadota*, *Campylobacterota*, *Bacteroidota*, *Aquificota*, *Chloroflexota*, and *Planctomycetota* aligns with other studies on deep-sea microbial communities^21,33,38,39^. *Desulfobacterota* was detected at >1% relative abundance in samples M37, M38, and M39, suggesting adaptation to sulfur-rich environments. Sample M39 exhibited the highest diversity of this phylum, with 22 MAGs classified as *Desulfobacteria*. M25 showed the greatest diversity within the archaeal phylum *Thermoproteota*, with 31 MAGs classified to this phylum, accounting for 6.47% of its metagenome. 29 of these MAGs belonged to *Sulfolobales*, a group specialized in sulfur metabolism and known for thriving in high-temperature, acidic, and alkaline environments^40,41^. *Halobacteriota* was another archaeal phylum frequently observed in our biofilms, exhibiting a relative abundance of 1.2% in sample M38, where 4 MAGs were identified, and 2.32% in M39, with 8 MAGs detected.

Community dissimilarity analysis based on Bray-Curtis and Sørensen indices revealed distinct clustering among the samples. Bray-Curtis dissimilarities, which account for relative abundance, showed that sample M26 was significantly different from the others (0.70−0.89), with high bootstrap confidence intervals (upper CI > 0.95 in most pairwise comparisons) (Supplementary Fig. 1). In contrast, samples such as M24, M36, and M39 exhibited lower dissimilarity values (e.g., M24 and M39, 0.49), indicating more similar community structures. This was supported by Sørensen dissimilarities, which are based on the presence or absence of taxa, with M26 showing high dissimilarity (e.g., M26 and M37, 0.81; M26 and M42, 0.86), suggesting a distinct taxonomic composition (Supplementary Fig. 2). Permutation tests showed that most pairwise similarities did not differ significantly (*p* > 0.1). These results indicate that M26 harbors a microbial community that is compositionally distinct from the other samples. Meanwhile, samples M24, M36, M38, and M39 appear to form a more cohesive group in terms of both shared taxa and relative abundances.

### Linking diversity and transcription of BGCs across hydrothermal biofilms

**Fig. 3:**
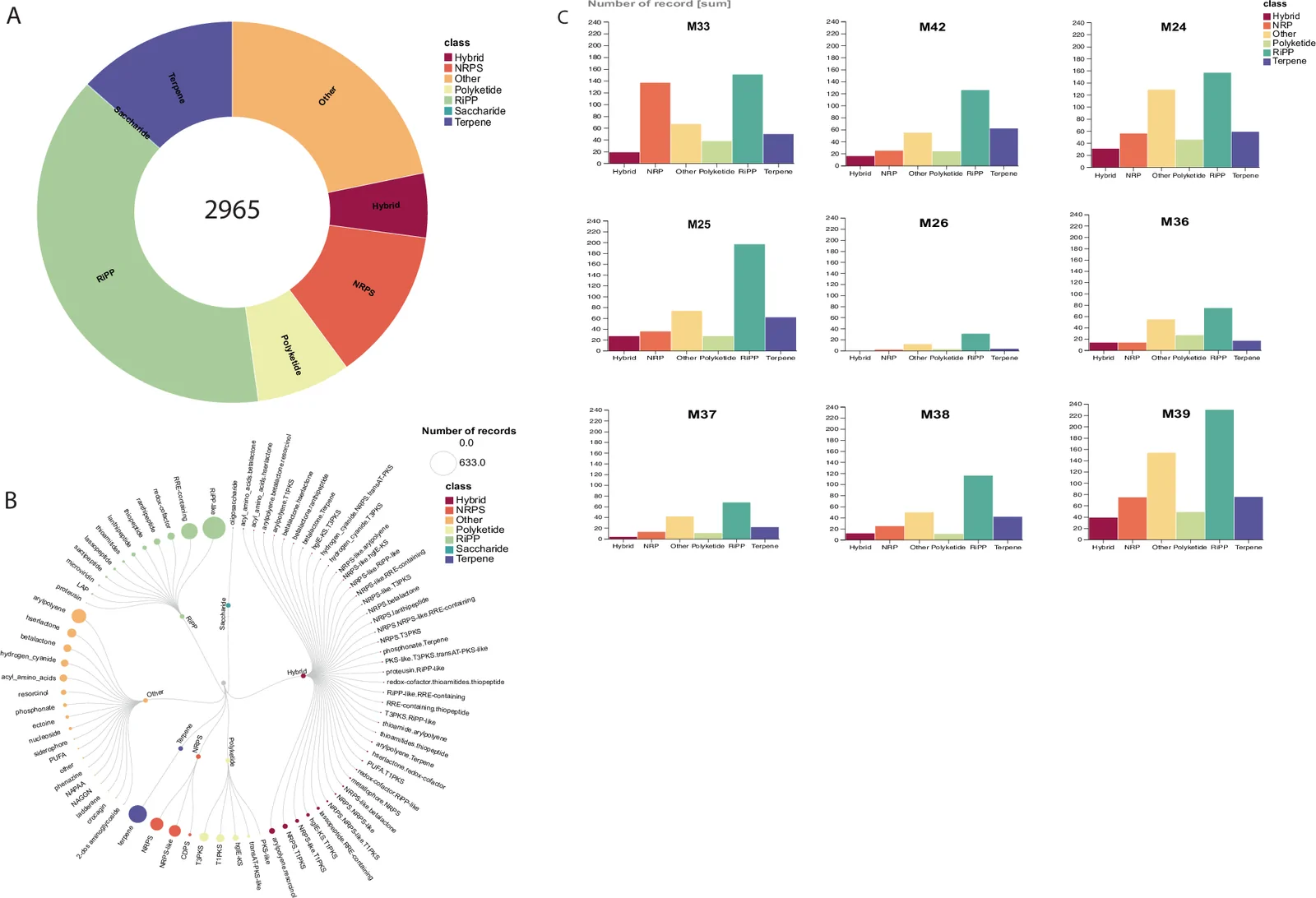
Diversity of BGCs and predicted products in AMOR biofilms. **A** Abundance of individual BGC classes, **B** Predicted product types, with circle sizes indicating relative abundance. **C** Composition of BGC classes across nine metagenomes, excluding saccharides. BGCs are categorized based on the biosynthetic classes of their core enzymes, as assigned by antiSMASH v7.0 and BiG-SLiCE v2.0.0.

**Fig. 4:**
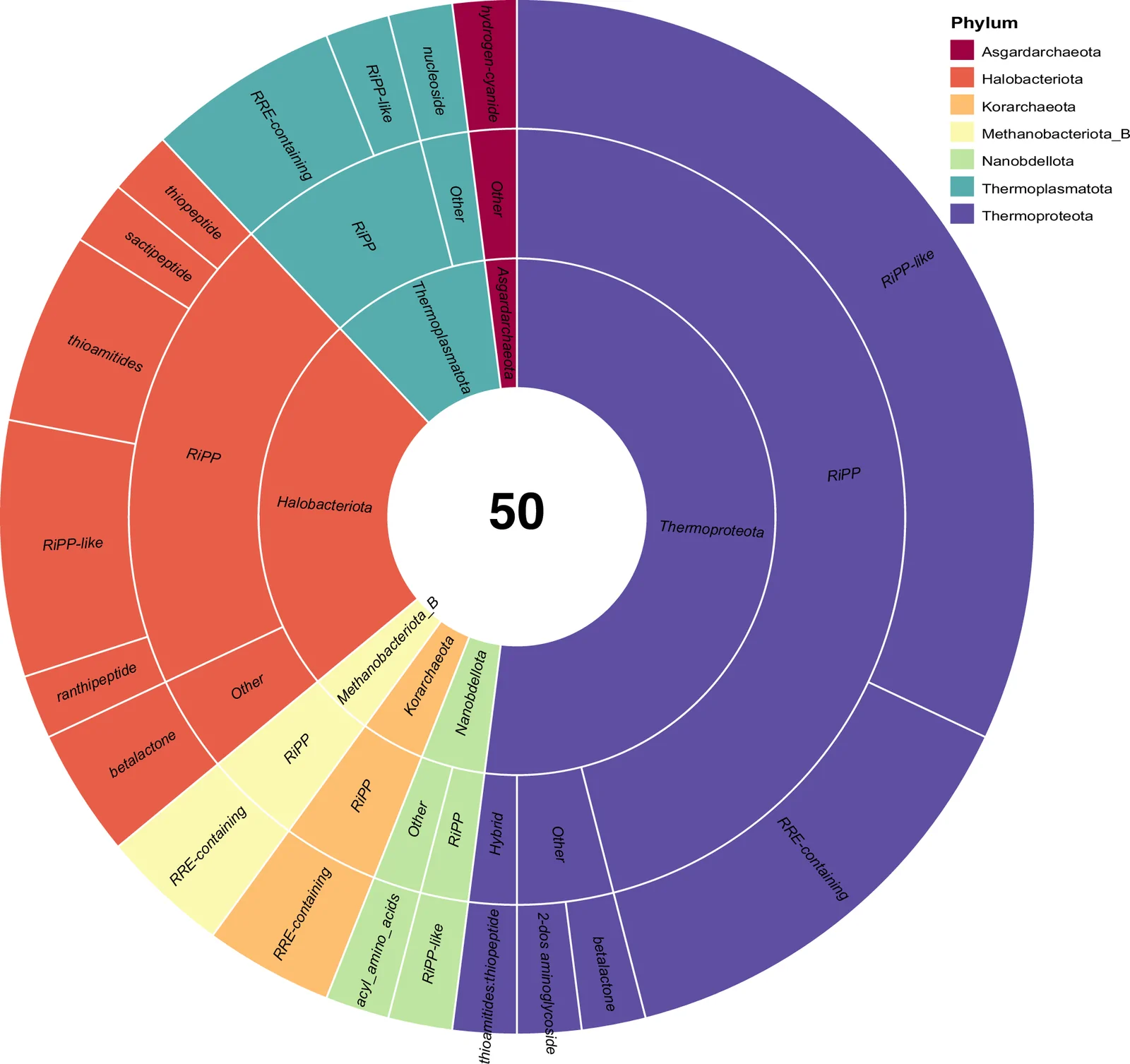
Distribution of BGCs and predicted metabolites in archaeal MAGs. The number and types of BGCs identified across archaeal phyla from nine biofilms, categorized by BGC class and predicted metabolite. Taxonomic classifications are based on GTDB release R226. BGC classes and predicted products were assigned using antiSMASH v7.0 and BiG-SLiCE v2.0.

Metagenomic analysis of nine hydrothermal biofilm samples revealed a total of 2965 BGCs across 870 MAGs (Fig. 3A). The dominant phylum, *Pseudomonadota*, encoded the largest number of BGCs (*n* = 1258), followed by *Bacteroidota* (*n* = 299) and *Myxococcota* (*n* = 277). BGCs were distributed across 46 of 51 bacterial and 7 of 10 archaeal phyla, with *Nitrospirota* exhibiting the highest BGC density. Only 11.97% (*n* = 355) of BGCs were complete (i.e., not located at contig edges, and thus less likely to be truncated), while the majority (*n* = 2610) were fragmented. RiPPs were the most abundant BGC class (*n* = 1151), followed by other (*n* = 638), Terpene (*n* = 394), NRP (*n* = 383), Polyketide (*n* = 236), Hybrid (*n* = 162), and Saccharide (*n* = 1) (Fig. 3B, Supplementary Fig. 6A, Supplementary Data 4). RiPP-like clusters, reclassified from bacteriocins in antiSMASH since the release of version 6.0^42^, were the most frequently predicted subtype (*n* = 633) (Fig. 3B; Supplementary Fig. 3A). RiPPs were consistently dominant across all biofilm samples (Fig. 3C), suggesting a conserved ecological role. In comparison, archaeal MAGs encoded 50 BGCs across seven phyla, with lower diversity and representation limited to RiPP, Other, and Hybrid classes (Fig. 4).

**Fig. 5:**
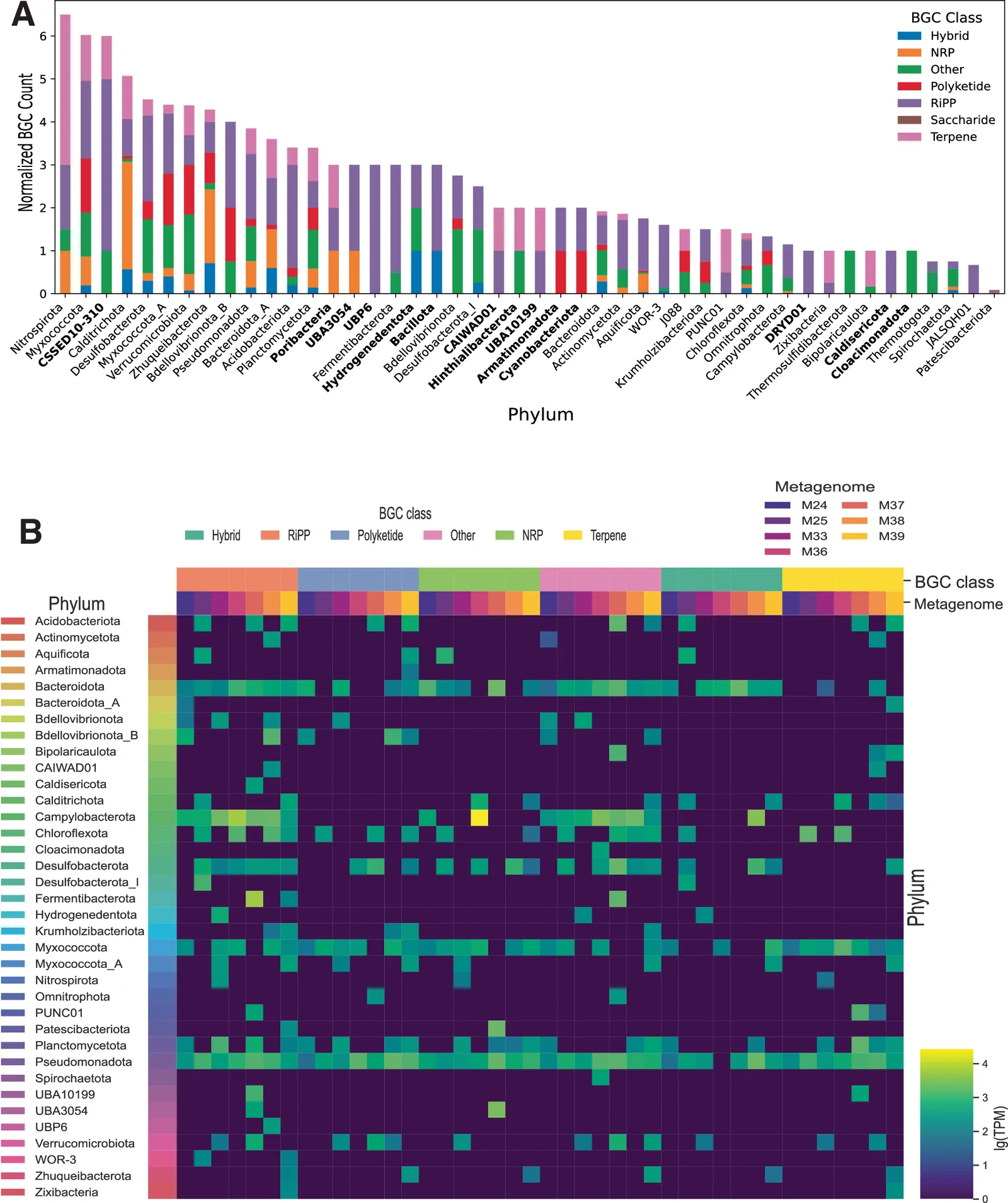
BGC richness and expression across Bacterial phyla in the AMOR biofilms. **A** normalized BGC counts across bacterial phyla in all samples, with phyla represented by a single MAG indicated in bold. Taxonomic classifications are based on GTDB release R226, and **B** expression profiles of bacterial BGC classes in AMOR biofilms. The heatmap shows the expression levels of predicted BGC classes across the seven AMOR biofilms. Expression levels were estimated using BiG-MAP v1.0.0 and normalized as average log₁₀-transformed TPM values. BGC detection and classification were performed using antiSMASH v7.0 and BiG-SLiCEv2.0.0.

**Fig. 6:**
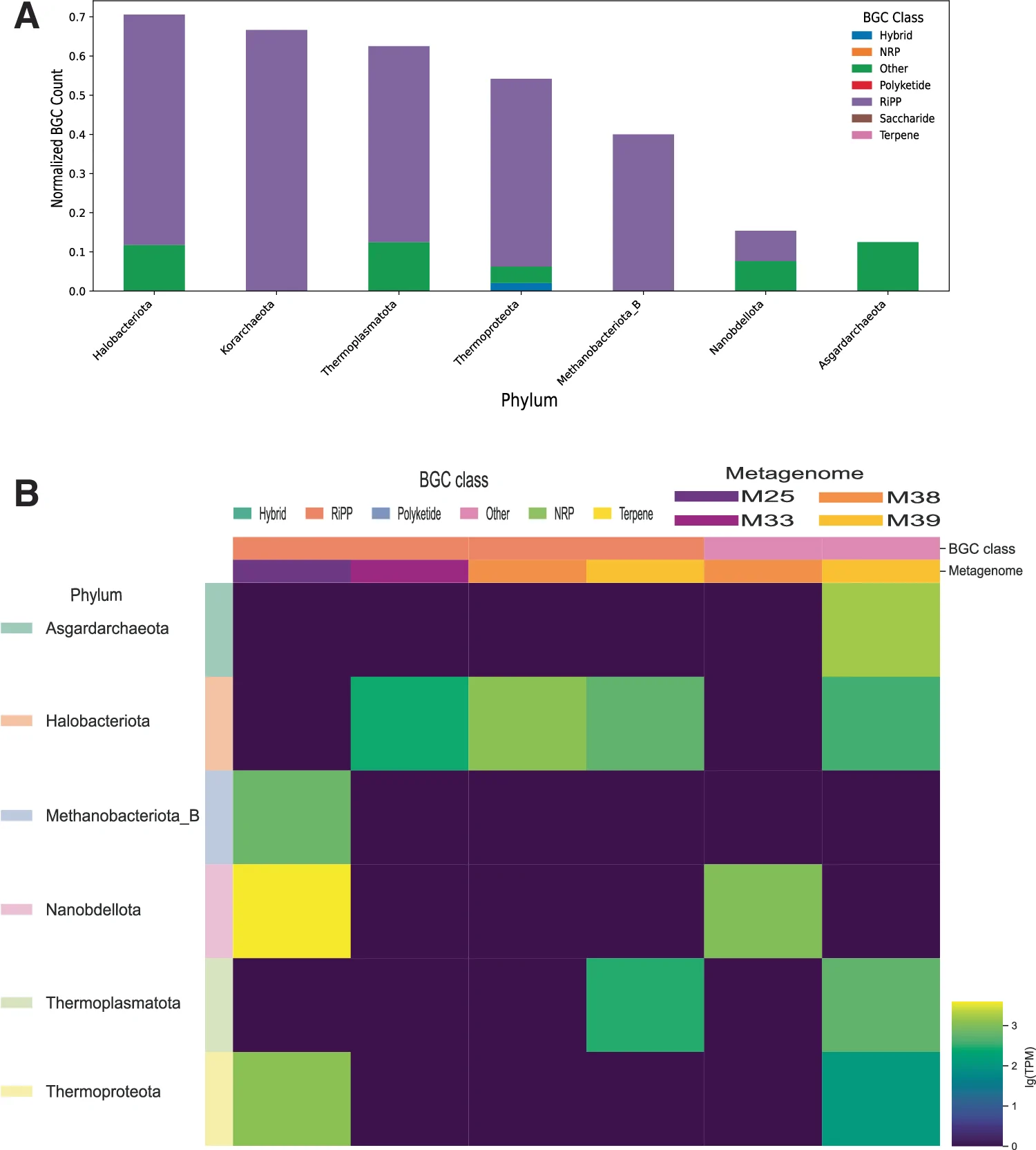
BGC richness and expression across Archaeal phyla in AMOR biofilms. **A** Normalized BGC counts across archaeal phyla in all samples, and **B** expression profiles of archaeal BGC classes in the AMOR biofilms. The heatmap shows the expression levels of predicted BGC classes across the seven AMOR biofilms. Expression levels were estimated using BiG-MAP v1.0.0 and normalized as the average log₁₀-transformed TPM values. Taxonomic classifications are based on the GTDB release R226; BGC detection and classification were performed using antiSMASH v7.0 and BiG-SLiCEv2.0.0.

To assess functional expression, metatranscriptomic profiling using long-read nanopore sequencing was successful on seven of the nine biofilm samples. A total of 1450 BGCs were actively transcribed across 519 MAGs, spanning 41 microbial phyla: 35 bacterial (Fig. 5) and 6 archaeal (Fig. 6). Expression patterns closely mirrored metagenomic abundance, whereby *Pseudomonadota*, which encoded the highest number of BGCs, also exhibited the highest transcriptional activity (*n* = 616 expressed BGCs), followed by *Bacteroidota* (*n* = 164), *Desulfobacterota* (*n* = 136), and *Myxococcota* (*n* = 135). Sample M39 showed the highest overall BGC expression (*n* = 411 clusters) (Fig. 5B). The MAG Barite_M33_B89 (*Pseudomonadota*, candidate genus *JABSOG01*) exemplified the link between genomic potential and transcriptional output. antiSMASH identified 107 BGC regions containing core biosynthetic domains in this MAG, spanning NRPS (*n* = 74), polyketides (*n* = 15), RiPPs (*n* = 8), Hybrids (*n* = 6), and Other (*n* = 4), and exhibited the highest number of expressed clusters (*n* = 75), suggesting that this MAG may represent a potentially important biosynthetic contributor within its biofilm community.

RiPPs were the most transcriptionally active BGC class (*n* = 532), consistent with their genomic prevalence (Figs. 5B and 6B). Expression levels, quantified as transcripts per million (TPM), are detailed in Supplementary Data 5. Statistical analysis (Kruskal–Wallis with Dunn’s post-hoc test) revealed significant differences in expression among BGC classes (Supplementary Fig. 6B; Supplementary Data 7). RiPPs showed significantly higher expression than Hybrid (*p* = 0.0238), Polyketide (*p* = 0.0008), and NRP (*p* = 0.0002) clusters after Bonferroni correction (Supplementary Data 7). Similarly, the Other class showed significantly higher expression compared to NRP (*p* = 0.0127) and Polyketide (*p* = 0.0179). No significant differences were observed among Hybrid, NRP, Polyketide, and Terpene groups (*p* > 0.05).

### Functional roles of expressed BGCs in biofilm adaptation

**Fig. 7:**
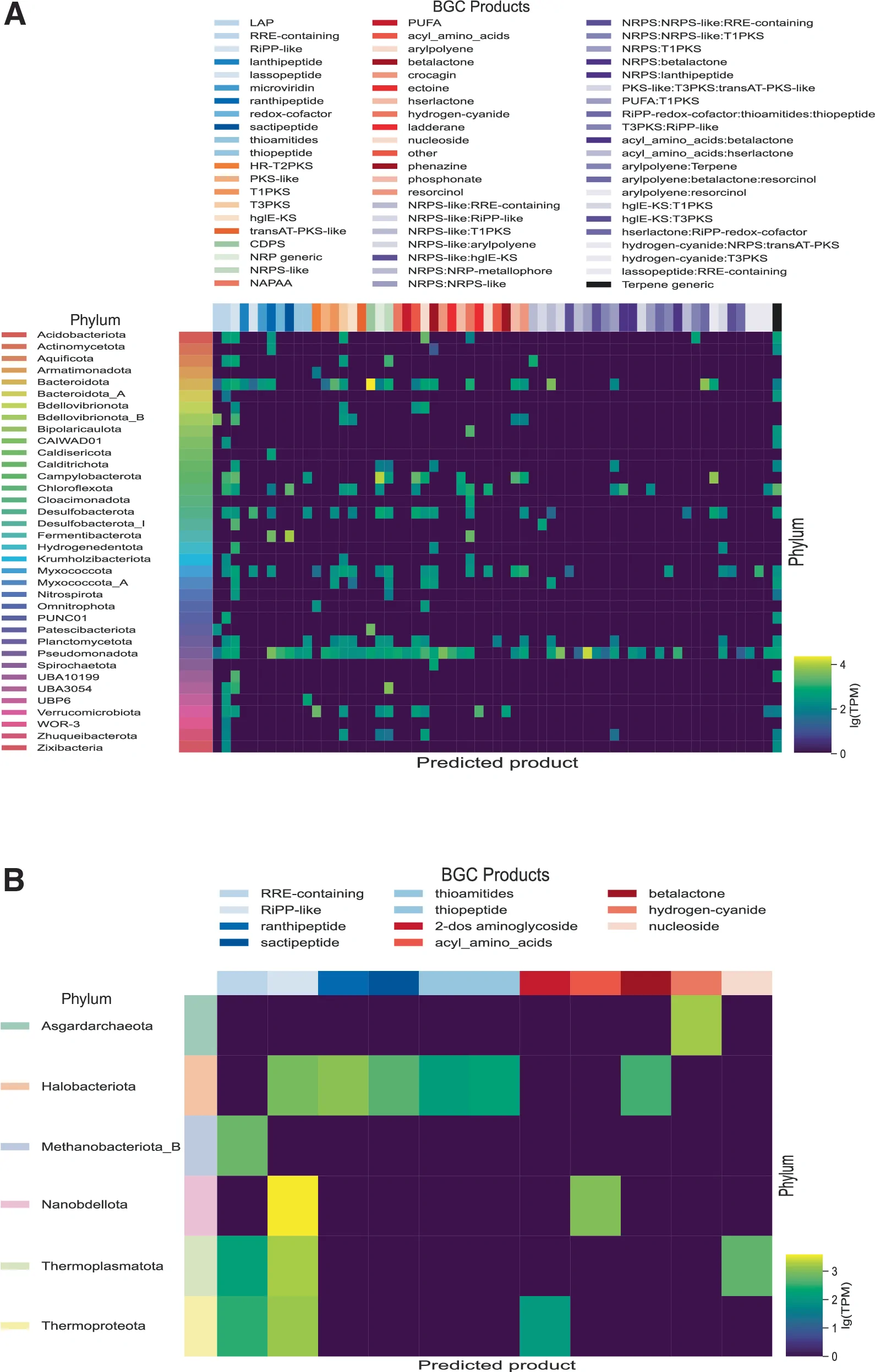
Expression profiles of BGC products in AMOR biofilms. Heatmaps show the expression levels of predicted BGC products across seven AMOR biofilms, separated by domain in **A** Bacteria and **B** Archaea. Expression levels were estimated using BiG-MAP v1.0.0 and normalized as average log₁₀-transformed TPM values.

Several BGCs linked to microbial adaptation were both genomically encoded and transcriptionally active. Aryl polyene (APE) BGCs (*n* = 253), known to enhance oxidative stress tolerance and biofilm formation^43^, were consistently expressed across all metatranscriptomes, particularly in *Pseudomonadota*, *Bacteroidota*, *Campylobacterota*, *Desulfobacterota*, *Myxococcota, Acidobacteriota, Bdellovibrionota, Planctomycetota, Verrucomicrobiota, Omnitrophota* and *Zhuqueibacterota* (Fig. 7, Supplementary Fig. 3B, Supplementary Data 5). Ectoine BGCs (*n* = 14), primarily from *Pseudomonadota*, were detected in eight metagenomes and actively transcribed in M24, M36, M37, and M38, suggesting roles in osmotic and thermal stress mitigation^44^. BGCs associated with nutrient acquisition and regulation included phosphonates (*n* = 17), thioamitides (*n* = 8), and siderophores (*n* = 2)^30,45^. Quorum sensing and biofilm regulation were supported by homoserine lactone (hserlactone) BGCs (*n* = 98) and ranthipeptides (*n* = 51)^46–48^. Hserlactone BGCs were present in all metagenomes, and transcriptional activity was detected in all seven screened metatranscriptomes, indicating the importance of this BGC in biofilm development (Supplementary Data 5).

Hydrothermal biofilms harbored a diverse array of potential antimicrobial BGCs, many of which were actively transcribed (Fig. 7). Among RiPP subclasses, we detected thiopeptides (*n* = 23)^49^, lanthipeptides (*n* = 20)^39,50^, lasso peptides (*n* = 8)^51^, sactipeptides (*n* = 6)^52^, and linear azol(in)e-containing peptides (LAPs; *n* = 4)^53^. Several of these RiPP families include members with known antimicrobial activities^50,54,55^. Additional antimicrobial classes included β-lactones (*n* = 73)^56^, N-acyl amino acids (*n* = 65)^57^, resorcinols (*n* = 34)^58^, NRPS (*n* = 203)^59^, and polyketide synthases (T1PKS: *n* = 78; T3PKS: *n* = 96)^60,61^. Although many of the detected BGCs are linked to antimicrobial activity in other organisms, their functional roles within these hydrothermal biofilms are not yet clear. Evidence indicates that such metabolites can act as signaling compounds at low concentrations, and some BGCs may remain silent unless triggered by specific environmental conditions^6,54^. The MAG Barite_M33_B89 (phylum *Pseudomonadota*, candidate genus *JABSOG01*) was a major contributor to antimicrobial potential, expressing BGCs across multiple classes (Supplementary Data 5). These BGCs spanned five classes: NRPS (74), polyketides (15), RiPPs (8), Hybrids (6), and Other (4). Given that NRPs, polyketides, and RiPPs are renowned for producing diverse antimicrobial compounds^8,30,62^, this understudied genus, JABSOG01, represents a compelling candidate for future studies on novel natural bioactive compounds. The MAG Aegir_M24_B72, classified within the phylum *Bacteroidota*, contained the longest BGC (105.77 kb; Supplementary Data 4), predicted to encode an NRPS–T1PKS hybrid.

### Network analysis of biosynthetic gene clusters

**Fig. 8:**
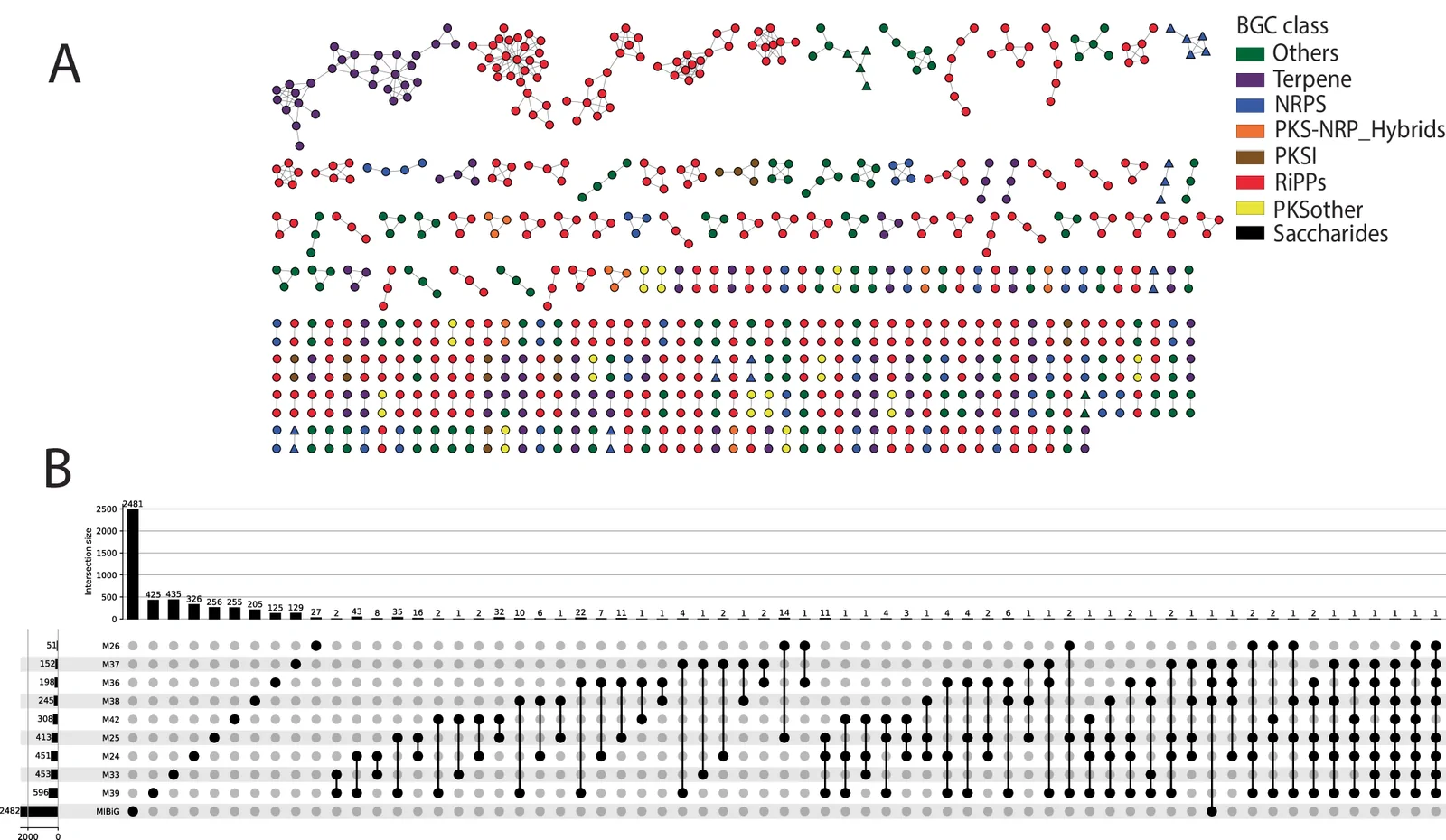
Cluster analysis of BGCs from AMOR biofilms. **A** Sequence similarity network of GCFs identified using BiG-SCAPE v1.1.5 (similarity cutoff = 0.3). Each node represents a BGC, colored by class as assigned by BiG-SCAPE. Circular nodes indicate BGCs from AMOR biofilms, and triangular nodes represent reference BGCs from the MIBiG v3.1 database. Singletons are excluded for clarity. **B** UpSet plot showing the number of shared GCFs across biofilms, based on the sequence similarity network.

To evaluate the uniqueness of BGCs recovered from biofilm communities, we performed a comprehensive network analysis comparing BGCs identified in nine biofilm samples with those curated in the MIBiG database (version 3.1). In total, 5450 BGCs were analyzed, comprising reference BGCs from MIBiG and 2953 BGCs from our metagenomic dataset. These BGCs were clustered into 4953 gene cluster families (GCFs), of which 4643 were singletons, and 310 contained two or more BGCs (Fig. 8A; Supplementary Fig. 7, Supplementary Data 6). Strikingly, only a single GCF included BGCs from both the biofilm-derived dataset and the MIBiG reference set, underscoring the pronounced divergence between these two sources. This minimal overlap highlights the distinct biosynthetic repertoire of biofilm-associated microorganisms and suggests the presence of novel metabolic pathways not represented in existing databases. The high proportion of singleton GCFs further supports the hypothesis that biofilm communities harbor a largely untapped reservoir of unique biosynthetic potential. Pairwise cluster sharing analysis revealed substantial variability in BGC composition among biofilm samples. For instance, samples M24 and M39 shared 75 GCFs, as did M25 and M39, while M24 and M25 shared 50 GCFs (Fig. 8B). Sample M36 exhibited moderate overlap with M39 (46 shared GCFs), M25 (25), and M24 (22), indicating partial biosynthetic similarity. In contrast, M26 was markedly distinct, sharing only 9 GCFs with M39 and between 2–4 GCFs with other samples. Notably, only a single GCF was shared between MIBiG and the biofilm samples M36, M37, and M38, further reinforcing the novelty of the biofilm-derived BGCs.

### Previously unrecognized biosynthetic capacities in archaea

A total of 60 BGCs associated with HCN synthesis were identified, including one in the archaeal MAG Aegir_M39_B184, taxonomically assigned to the class *Heimdallarchaeia* within the phylum *Asgardarchaeota* (Supplementary Fig. 4A and 4B). HCN is a well-characterized virulence factor predominantly found in bacterial taxa such as *Pseudomonas* spp^63^. Its biosynthesis is mediated by HCN synthase, encoded by the canonical *hcnABC* operon, which catalyzes the oxidative decarboxylation of glycine to yield carbon dioxide and HCN^64^, a process termed cyanogenesis. This pathway is tightly regulated by environmental conditions, including iron and phosphate availability, as well as microaerobic conditions^64^.

In contrast to the tripartite *hcnABC* operon of *Pseudomonas fluorescens* (BGC0002345, MIBiG 3.1), the putative HCN synthase in Aegir_M39_B184 is encoded by two genes, *hcnAB* and *hcnC* (Protein file: https://doi.org/10.6084/m9.figshare.30788630). Structural modeling using the AlphaFold3^65^ webserver (https://deepmind.google/science/alphafold/) and comparative superimposition with the structural model of *hcnA* and *hcnB* from *P. fluorescens* (Supplementary Fig. 5A), support the presence of a complete and potentially functional HCN synthase. A high structural similarity was also observed for the *hcnC* subunit (https://doi.org/10.6084/m9.figshare.30788579). Furthermore, transcriptomic data confirmed expression of both genes (Supplementary Data 5). Although the identified proposed *hcn*AB and *hcn*C genes were located on a contig edge, high-scoring blastp hits to other Archaea, in particular *Heimdallarchaeia* from Guayamas Basin^66^ containing additional flanking genes, support the presence of a complete HCN cluster in Archaea (Supplementary Data 8). Comparative genomic analysis using antiSMASH v8.0.4, revealed a homologous BGC architecture in another *Asgardarchaeota* genome, *Candidatus* Prometheoarchaeum syntrophicum strain MK-D1 (NZ_CP042905.1), in addition to the Guayamas MAGs (Supplementary Fig. 4C). A superimposed model of *hcn*AB from Aegir_M39_B184 and a *Candidatus* Heimdallarchaeaceae archaeon MAG from Guayamas Basin (MHA1303415.1) not located at a contig edge, show high structural similarity (Supplementary Fig. 5B). Overall, this supports the potential for archaeal cyanogenesis.

Additionally, the MAG Aegir_M39_B193, classified within the phylum *Thermoproteota*, harbors a BGC predicted to encode the biosynthesis of 2-deoxystreptamine, a central scaffold of aminoglycoside antibiotics such as streptomycin, kanamycin, and gentamicin^67^. This compound is predominantly associated with bacterial lineages, particularly within the phyla *Actinomycetota* and *Bacillota*^68–70^. The presence of aminoglycoside biosynthetic pathways in archaeal genomes is exceedingly rare and, to our knowledge, has not previously been reported in hyperthermophilic archaea such as *Thermoproteota*^7^, particularly with supporting transcriptomic evidence. To limit the probability of these features being part of contaminating contigs, an additional quality assessment was made using MAGpurify2. The genes of the HCN synthase and the aminoglycoside pathway were retained on contigs with low contaminant probability scores (both 0.05), classified to the same taxa as the overall MAGs, class *Heimdallarchaeia* within the phylum *Asgardarchaeota* and class *Thermoproteia* within the phylum *Thermoproteota*, respectively.

## Discussion

Microbial biofilms from extreme environments, shaped by extreme environmental pressures and adapted through cooperative and competitive interactions^71,72^, represent promising reservoirs of novel bioactive compounds. Despite their potential, the biosynthetic landscape of Arctic deep-sea hydrothermal biofilms remains largely unexplored. In this study, we investigated nine biofilms from three Arctic hydrothermal vent sites, analyzing BGC diversity across 1140 archaeal and bacterial MAGs. Our findings underscore the potential biosynthetic richness of these biofilms and highlight their promise as untapped sources of antimicrobial agents. The microbial consortia inhabiting Arctic hydrothermal biofilms exhibit remarkable taxonomic and metabolic diversity, encompassing a broad-spectrum of bacterial and archaeal phyla. BGCs serve as adaptive molecular tools, conferring selective advantages that promote microbial persistence and competitive success in these extreme environments. Notably, the M26 biofilm displayed a distinct community composition despite originating from the same vent field (Ægir vent field) as six other samples, suggesting that biofilm assembly is influenced not only by environmental parameters but also by intra-community interactions. Diversity metrics based on abundance and presence/absence data support niche specialization, as shown by the unique BGC repertoire identified in M26. These patterns point to a metabolically distinct microbial consortium structured within this biofilm. The average number of BGCs per MAG (2.60) in the AMOR biofilms exceeds the global marine average, emphasizing the influence of habitat on secondary metabolism^34^. For instance, Antarctic soils yielded 1,417 bacterial BGCs with no archaeal BGCs, whereas cold seep communities contained 238 archaeal BGCs among 487 MAGs, predominantly encoding RiPPs^30,73^. These observations suggest that selective pressures in deep-sea ecosystems favor the retention and expression of RiPP BGCs as a survival strategy. RiPPs emerged as the most abundant BGC class across all nine biofilms, contrasting with other extreme environments such as Antarctic soils and Whalers Bay sediments, where terpenes predominate^73,74^. This divergence suggests that adaptation to hydrothermal conditions may favor RiPP biosynthesis, potentially due to their roles in microbial antagonism and communication. The consistent dominance of RiPPs across samples, rather than being driven by outliers, indicates community-wide selection for these compounds. This pattern aligns with global surveys of marine microbial genomes, which also report RiPPs and terpenes as the most prevalent BGC classes^7,34^. Moreover, RiPP BGCs in our dataset encode a diverse array of potential antimicrobial peptides, some of which have previously demonstrated in vitro antimicrobial activity^34^. For example, thiopeptides, detected in both the metagenomic and metatranscriptomic datasets, are well-known for their potent antibiotic activity against Gram-positive bacteria^49,75,76^. Sactipeptides, also detected in both the metagenomic and metatranscriptomic datasets, exhibit broad-spectrum activity against Gram-positive and Gram-negative bacteria^50,77–79^. While some members of these RiPP classes are known to exhibit antimicrobial activity, such functional properties cannot be inferred without detailed characterization of individual clusters. The expression of these antimicrobial peptides in AMOR biofilms suggests that they play an important role in microbial competition within the biofilm.

From an ecological perspective, APE BGCs, encoding widespread bacterial pigments^43^, were consistently detected and transcriptionally active across all samples, indicating their potential role in microbial fitness and biofilm stability. Phosphonate BGCs, which contribute to marine phosphorus cycling^45^, were identified in 17 MAGs across five biofilms and seven bacterial phyla. Due to their frequent occurrence in marine and cold seep prokaryotic genomes^30,31^, transcriptomic activity was detected from three samples (M25, M33, and M39) in five bacterial phyla (*Pseudomonadota*, *Bacteroidota, Myxococcota, Campylobacterota*, and *Bdellovibrionota*). In contrast, siderophore BGCs were rare, with only two detected (M25 and M26, phylum *Pseudomonadota*) and no expression detected, suggesting limited iron acquisition strategies in these biofilms.

Thioamitide BGCs, previously reported in the archaeal phylum *Halobacteriota* from cold seeps^30^, were also identified in our dataset, spanning bacterial phyla *Actinomycetota* and *Pseudomonadota*, as well as archaeal phyla *Halobacteriota* (order *Alkanophagales*). These clusters mediate thioamidation, a biochemical process replacing an amide oxygen with sulfur^80^. Prior studies proposed that *Halobacteriota* members utilize thioamide pathways to modify methyl-coenzyme M reductase, facilitating anaerobic oxidation of methane (AOM)^30^. Consistent with this, MAGs Barite_M33_B83 and Aegir_M39_B54 (order *Alkanophagales*) exhibited transcriptomic expression of a thioamitide BGC, indicating active AOM metabolism. Another *Alkanophagales* MAG (Aegir_M38_B4) expressed ranthipeptides, metabolites previously considered bacterial and only recently reported in archaea^48,81^, expanding their known distribution and suggesting a role in *Halobacteriota* adaptability. Collectively, these findings demonstrate that BGC-encoded metabolites contribute to microbial resilience in extreme environments and influence biofilm dynamics through selective pressures, interspecies signaling, and physiological protection. Furthermore, NRPS BGCs, another prominent class of antimicrobial producers, were detected in eight of nine biofilms. M33 was notably enriched, harboring 91 of the 203 NRP generic clusters. Transcriptomic data revealed active expression of 128 NRP generic BGCs, including 65 from M33, affiliated with diverse hydrothermal vent phyla such as *Pseudomonadota*, *Planctomycetota*, *Campylobacterota*, *Chloroflexota*, *Calditrichota*, *Bacteroidota*, *Desulfobacterota, Myxococcota, Verrucomicrobiota*, and *Zhuqueibacterota*. These data suggest that NRPS-derived metabolites play a significant role in microbial competition and niche establishment. Terpenes, though less abundant than RiPPs and NRPS, were detected in all metagenomes (394 BGCs), exclusively in bacterial taxa. Their known roles in microbial signaling and defense^82^, coupled with transcriptomic evidence from 162 BGCs from all screened metatranscriptomes, highlight their functional relevance^34^.

The discovery of HCN biosynthetic pathways in archaeal genomes expands the known metabolic repertoire of this domain and challenges the long-standing assumption that cyanogenesis is restricted to bacteria and eukaryotes. This suggests that archaea may utilize HCN as a chemical mediator in ecological interactions, particularly within the competitive and chemically dynamic environment of deep-sea biofilms. Given HCN’s established role in microbial antagonism and defense^83^, its presence in archaea raises intriguing questions about convergent evolution or horizontal gene transfer events. The broader taxonomic distribution of cyanogenic BGCs observed here, extending beyond traditional producers such as *Pseudomonas* and *Cyanobacteria*^84^, indicates that HCN synthesis may be a more widespread and ecologically relevant trait in marine microbial communities. The expression of these BGCs in multiple biofilm samples further supports their functional role in situ, suggesting that cyanogenesis contributes to niche differentiation, microbial competition, and defense strategies in hydrothermal ecosystems. These findings prompt a reevaluation of archaeal roles in chemically mediated interactions and highlight the need for further investigation into the evolutionary origins and regulatory mechanisms of archaeal cyanogenesis. However, additional biochemical and/or cultivation efforts, integrating comparative genomics, transcriptomics, and metabolomics, are needed to verify the HCN pathway in archaea and to elucidate the functional significance of HCN production and its impact on biofilm community dynamics.

In summary, the diversity and expression of BGCs across AMOR biofilms reflect finely tuned adaptive strategies to the physicochemical pressures of hydrothermal vent ecosystems. The broad-spectrum of predicted bioactive compounds, many with potential antimicrobial properties, positions these biofilm-associated microbiomes as a valuable and underexplored resource for biotechnological and pharmaceutical innovation. As our analyses are based on MAGs of varying completeness, some predicted BGCs (e.g., those located at contig edges) may represent partial clusters or isolated biosynthetic genes rather than complete operons. This limitation is inherent to rule-based prediction tools such as antiSMASH and may also affect downstream clustering and novelty assessment using BiG-SCAPE^85^. Therefore, while the overall diversity of predicted BGCs in AMOR biofilms remains robust, the absolute number of clusters should be interpreted with caution, and future work is required to validate their completeness and presence in situ.

## Methods

### Sampling sites and collection

Biofilm samples were collected at three hydrothermal locations, both from shallow water and deep-sea, along the AMOR. In the current study 9, biofilms were collected: one from the shallow-water even Sisters vent field (125 m depth)^86^, one from the diffuse venting area at the Loki’s Castle vent field (Barite field, 2340 m depth)^87^, and seven samples from the Ægir vent field (2310 m depth)^88^ (Table 1). Samples were collected using the Ægir6000 ROV on board the R/V G.O. SARS, equipped with a biosyringe (a hydraulic sampling cylinder) connected to the ROV manipulator arm. Sampling was conducted during sampling campaigns in 2017, 2020, 2021 and 2022 to AMOR, led by the Centre for Deep Sea Research at the University of Bergen. Samples retrieved onboard R/V G.O. SARS were centrifuged at 6000 x *g* for 5 minutes and the supernatant wewasemoved. Aliquots were frozen in liquid nitrogen and stored at −80 °C until processing.

### Genome-resolved metagenomics

DNA was extracted using FastDNA Spin Kit for Soil (MP Biomedicals) from frozen samples and sent to the Norwegian Sequencing Centre (University of Oslo, Norway) for shotgun metagenomic sequencing. 150-bp paired-end sequencing was performed using an Illumina NovaSeq S4 flow cell. Raw reads were scanned and filtered for quality, duplication rate, and adapter contamination using fastp (v0.23.2)^89^.

Filtered reads from individual biofilms were assembled into contigs by MEGAHIT (v1.2.9)^90^ with parameters *--min-contig-len 2000 --presets meta-large*. Contigs with lengths longer than 2000 bp were subjected to automatic MAG reconstruction using metaWRAP (v1.3.2)^91^ which implements the combinatorial use of MaxBin2 (v2.2.6)^92^, CONCOCT (v1.0.0)^93^, and MetaBat2 (v2.12.1)^94^ for MAG refinement. The quality of each MAG was estimated with CheckM1 (v1.0.12) and CheckM2 (1.1.0)^95^ in addition to the presence/absence of rRNA and tRNA determined by Barrnap v0.9^96^ and tRNAscan v2.0.12^97^, respectively. Low-quality genomes following the MIMAG standard^98^ (completeness <50% and contamination >10%) were removed from downstream analysis, with the exception of 25 MAGs first estimated to be medium-quality by checkM1. High-quality MAGs are defined with completeness ≥90% and contamination ≤5%, and include at least one copy of 16S, 23S, and 5S rRNA genes and ≥18 unique tRNAs. Medium-quality MAGs are defined with completeness ≥90% and contamination ≤10%. In addition to the quality assessment described above, key MAGs were further purified using MAGpurify2^99^ to reduce the possibility of contaminating contigs using --probability_threshold 0.7 for removal of contaminants.

De-replication of MAGs from all 9 biofilms was performed at the species level using dRep (v3.4.0)^100^ with parameter settings *-l 40000 -comp 50 -con 10 --S_ani 98*. Although stringent, an ANI value of 98% was selected to balance genome diversity while minimizing cross-mapping issues and avoiding redundancy >98%^101^. Relative abundance of de-replicated MAGs in each biofilm was estimated by mapping filtered paired-end reads from each biofilm sample to MAGs using CoverM v0.6.1 (https://github.com/wwood/CoverM). Taxonomic classification was performed using the Genome Taxonomy Database^102,103^ via the Genome Taxonomy Database Toolkit^104^ (GTDB-Tk, v2.5.2 – GTDB R226 taxonomy) with the classify_wf using default parameters.

### RNA extraction, complementary DNA synthesis, and nanopore sequencing

RNA was extracted using the Quick-DNA/RNA Miniprep Kit (Zymo Research, USA), and subsequent RNA purification steps utilized HighPrep RNA Elite beads (MagBio Genomics Inc., USA). Ribosomal RNA (rRNA) was removed from total RNA samples for all samples, except for c25, with the Pan-Prokaryote riboPOOL (Bacteria and Archaea) (siTOOLs Biotech GmbH, Germany). Polyadenylation was performed using *E. coli* Poly(A) Polymerase (New England Biolabs, USA). Complementary DNA (cDNA) synthesis was carried out using the Ligation Sequencing Kit V14, and sequencing was performed on the MinION Mk1C device (Oxford Nanopore Technologies plc, UK). The full description of the adapted protocol following the Nanopore sequencing guidelines for microbial metatranscriptomics is available at protocols.io (https://doi.org/10.17504/protocols.io.dm6gpzz9plzp/v1). Sequencing utilized the Oxford Nanopore Flow Cell R10.4.1 (FLO-MIN114, Oxford Nanopore Technologies, UK).

### Biosynthetic gene clusters; screening and expression profiling

Gene annotation was conducted on MAG sequences using the Prokka annotation pipeline (version 1.14.6)^105^. The dataset was divided into two separate runs for archaeal and bacterial MAGs. The following parameters were applied: *--addgenes*, *--force*, *--increment* 10, --compliant, *--centre* NorSeq, *--norrna*, and *--notrna*. Secondary metabolite BGCs were identified and analyzed using antiSMASH (version 7.0)^106^ with the following options: *--cc-mibig, --cb-general, --cb-knownclusters, --cb-subclusters, --asf, --pfam2go, --smcog-trees, --rre*, and *--genefinding-tool* prodigal. Predicted BGCs were subjected to downstream analysis with BiG-SLiCE (version 2.0.0)^107^ to extract features, construct GCF models, and assign GCFs.

A network analysis of recovered BGCs was performed using BiG-SCAPE v1.1.5^108^. The program was executed in ‘--auto’ mode. Networks were computed with the ‘mix’ and ‘mibig’ options, which analyze all classes together and incorporate MIBiG v3.1^109^ BGCs into the network. The ‘--include singletons’ option was enabled to visualize singleton BGCs. Multiple raw distance cutoff values (ranging from 0.1 to 1.0) were tested, and networks generated with a 0.3 cutoff were selected for further analysis. Lower cutoff values are more suitable for grouping BGCs that produce identical compounds, whereas higher cutoffs provide a broader view of related product families. The resulting sequence similarity matrices were visualized using Cytoscape v3.10.3^110^. Additionally, the network annotation table generated by BiG-SCAPE was included to differentiate between the two sets of BGCs and to categorize different BGC classes.

Nanopore sequencing reads were basecalled using Dorado (version 0.5.1) (https://github.com/nanoporetech/dorado) with low-quality reads removed (*--min-qscore 10*) and adapter sequences trimmed (*--trim*). Ribosomal RNA sequences were filtered out using SortMeRNA v.4.3.6^111^. The resulting cDNA sequences were mapped against their corresponding metagenomic datasets using BiG-MAP (v.1.0.0)^112^ to profile the expression of BGCs. The BiG-MAP.map pipeline was modified to replace the default aligner with Minimap2 (v.2.26-r1175)^113,114^ to ensure more accurate mapping of Nanopore long reads. Modifications to the BiG-MAP.map module were permitted under the tool’s MIT license, and the modified script is provided in the Data Availability section of this paper. The relative abundance of biosynthetic genes across metagenomes was also estimated using the BiG-MAP.map module. For Nanopore metatranscriptomic reads, the -U option (unpaired mode) was applied, while Illumina metagenomic reads were processed using the -I1 and -I2 paired-end options. Abundance values were reported as TPM for metatranscriptomic datasets and genes per million (GPM) for metagenomic datasets.

### Statistical analyses and data visualizations

All statistical analyses were conducted using Python (version 3.13.2). Principal component analysis of microbial communities across biofilm samples was performed using the scikit-learn package (v.1.6.1). Bray–Curtis dissimilarities based on relative abundances of MAGs were calculated using the pdist function from the scipy package (v.1.15.2). Pairwise community similarity based on microbial order presence-absence data was quantified using the Dice–Sørensen coefficient. Statistical significance of the observed similarities was assessed using a permutation test (1000 permutations per pair). Kruskal–Wallis tests comparing the expression of different BGC classes were performed using the kruskal function from the scipy package. Post-hoc Dunn’s tests to identify significant pairwise differences among groups were conducted with the scikit-posthocs package (v.0.11.4), using TPM for each BGC class across samples. Data visualizations were created using RAWGraphs (v.2.0.0)^115^ and Python’s Matplotlib library (v.3.10.1), with all source data provided in the supplementary materials. No statistical method was used to predetermine sample size. No data were excluded from the analyses. The experiments were not randomized, and blinding was not relevant to the current study. To ensure the reproducibility of the metatranscriptomic pipeline used in the study, the full protocol is made available on protocols.io: https://doi.org/10.17504/protocols.io.dm6gpzz9plzp/v1.

### Reporting summary

Further information on research design is available in the Nature Portfolio Reporting Summary linked to this article.

## Supplementary information

Supplementary Information

Description of Additional Supplementary Files

Supplementary Data 1

Supplementary Data 2

Supplementary Data 3

Supplementary Data 4

Supplementary Data 5

Supplementary Data 6

Supplementary Data 7

Supplementary Data 8

Reporting Summary

Transparent Peer Review file

## Source data

Source Data

## Data Availability

Metadata of all samples and metagenomes in the study is deposited at NCBI under BioProjects listed in Table 1. Sequence data of individual MAGs has been deposited under the respective BioSample accession numbers listed in Supplementary Data 3, and all raw sequencing read data has been deposited to the Sequence Read Archive (SRA) with the following accession numbers: SRR36181650 (M24) (https://www.ncbi.nlm.nih.gov/sra/?term=SRR36181650), SRR36183670 (M25) (https://www.ncbi.nlm.nih.gov/sra/?term=SRR36183670), SRR36184633 (M26) (https://www.ncbi.nlm.nih.gov/sra/?term=SRR36184633), SRR36184876 (M33) (https://www.ncbi.nlm.nih.gov/sra/?term=SRR36184876), SRR36184925 (M36) (https://www.ncbi.nlm.nih.gov/sra/?term=SRR36184925), SRR36185683 (M37) (https://www.ncbi.nlm.nih.gov/sra/?term=SRR36185683), SRR36199626 (M38) (https://www.ncbi.nlm.nih.gov/sra/?term=SRR36199626), SRR36199942 (M39) (https://www.ncbi.nlm.nih.gov/sra/?term=SRR36199942) and SRR36203984 (M42) (https://www.ncbi.nlm.nih.gov/sra/?term=SRR36203984). Nanopore cDNA raw reads have been deposited to SRA under accession numbers: SRR36202719 (c24) (https://www.ncbi.nlm.nih.gov/sra/?term=SRR36202719), SRR36203181 (c25) (https://www.ncbi.nlm.nih.gov/sra/?term=SRR36203181), SRR36217904 (c33) (https://www.ncbi.nlm.nih.gov/sra/?term=SRR36217904), SRR36218254 (c36) (https://www.ncbi.nlm.nih.gov/sra/?term=SRR36218254), SRR36218304 (c37) (https://www.ncbi.nlm.nih.gov/sra/?term=SRR36218304), SRR36218718 (c38) (https://www.ncbi.nlm.nih.gov/sra/?term=SRR36218718), SRR36218944 (c42) (https://www.ncbi.nlm.nih.gov/sra/?term=SRR36218944). A FigShare project (https://doi.org/10.6084/m9.figshare.c.8159183) has been established for the study which includes GenBank and index files of all identified BGCs, in addition to the network file generated from BiG-SCAPE (https://doi.org/10.6084/m9.figshare.30762029), a protein sequence file of the *hcnAB* and *hcnC* from the *Heimdallarchaeion* Aegir_M39_B184 (https://doi.org/10.6084/m9.figshare.30788630), and a figure of the superimposed AlphaFold3 models of the *hcnC* from the *Heimdallarchaeion* Aegir_M39_B184 and *Pseudomonas fluorescence* PS619 (https://doi.org/10.6084/m9.figshare.30788579). Source data are provided with this paper.

## Code availability

The source code and data for all statistical analyses are available through Zenodo at https://doi.org/10.5281/zenodo.20057600^116^. The Zenodo repository represents the archived version of the GitHub repository associated with this paper and ensures long-term accessibility and reproducibility.

## References

[1] Murray, C. J. L. et al. Global burden of bacterial antimicrobial resistance in 2019: a systematic analysis. * Lancet* **399**, 629–655 (2022). 10.1016/S0140-6736(21)02724-0 PMC8841637 35065702

[2] Hemmerling, F. & Piel, J. Strategies to access biosynthetic novelty in bacterial genomes for drug discovery. *Nat. Rev. Drug Discov.* **21**, 359–378 (2022). 10.1038/s41573-022-00414-6 35296832

[3] Newman, D. J. & Cragg, G. M. Natural products as sources of new drugs over the nearly four decades from 01/1981 to 09/2019. *J. Nat. Prod.* **83**, 770–803 (2020). 10.1021/acs.jnatprod.9b01285 32162523

[4] Medema, M. H. et al. Minimum information about a biosynthetic gene cluster. *Nat. Chem. Biol.* **11**, 625–631 (2015). 10.1038/nchembio.1890 PMC5714517 26284661

[5] Sharrar, A. M. et al. Bacterial secondary metabolite biosynthetic potential in soil varies with phylum, depth, and vegetation Type. *mBio* **11**, e00416-20 (2020). 10.1128/mBio.00416-20 PMC7298704 32546614

[6] Quinn, G. A. & Dyson, P. J. Going to extremes: progress in exploring new environments for novel antibiotics. *Npj Antimicrob. Resist*. **2**, 8 (2024). 10.1038/s44259-024-00025-8 PMC11721673 39843508

[7] Chen, J. et al. Global marine microbial diversity and its potential in bioprospecting. *Nature* **633**, 371–379 (2024). 10.1038/s41586-024-07891-2 PMC11390488 39232160

[8] Sayed, A. M. et al. Extreme environments: microbiology leading to specialized metabolites. *J. Appl. Microbiol.* **128**, 630–657 (2020). 10.1111/jam.14386 31310419

[9] Jannasch, H. W. & Taylor, C. D. Deep-sea microbiology. *Annu. Rev. Microbiol.* **38**, 487–487 (1984). 10.1146/annurev.mi.38.100184.002415 6437324

[10] Corliss, J. B. et al. Submarine thermal springs on the Galápagos Rift. *Science* **203**, 1073–1083 (1979). 10.1126/science.203.4385.1073 17776033

[11] Ding, J. et al. Microbial community structure of deep-sea hydrothermal vents on the ultraslow spreading Southwest Indian Ridge. *Front. Microbiol.* **8**, 1012 (2017). 10.3389/fmicb.2017.01012 PMC5468387 28659873

[12] Crépeau, V. et al. Diversity and function in microbial mats from the Lucky Strike hydrothermal vent field: Diversity and function in Lucky Strike mats. *FEMS Microbiol. Ecol.* **76**, 524–540 (2011). 10.1111/j.1574-6941.2011.01070.x 21348883

[13] Dick, G. J. The microbiomes of deep-sea hydrothermal vents: distributed globally, shaped locally. *Nat. Rev. Microbiol.* **17**, 271–283 (2019). 10.1038/s41579-019-0160-2 30867583

[14] Stokke, R. et al. Functional interactions among filamentous *E psilonproteobacteria* and *B acteroidetes* in a deep-sea hydrothermal vent biofilm. *Environ. Microbiol.* **17**, 4063–4077 (2015). 10.1111/1462-2920.12970 26147346

[15] Li, M., Jain, S. & Dick, G. J. Genomic and transcriptomic resolution of organic matter utilization among deep-sea bacteria in guaymas basin hydrothermal plumes. *Front. Microbiol*. **7**, 1125 (2016). 10.3389/fmicb.2016.01125 PMC4962555 27512389

[16] Meier, D. V. et al. Heterotrophic *Proteobacteria* in the vicinity of diffuse hydrothermal venting. *Environ. Microbiol.* **18**, 4348–4368 (2016). 10.1111/1462-2920.13304 27001712

[17] Anderson, R. E. et al. Genomic variation in microbial populations inhabiting the marine subseafloor at deep-sea hydrothermal vents. *Nat. Commun.* **8**, 1114 (2017). 10.1038/s41467-017-01228-6 PMC5655027 29066755

[18] Dombrowski, N., Seitz, K. W., Teske, A. P. & Baker, B. J. Genomic insights into potential interdependencies in microbial hydrocarbon and nutrient cycling in hydrothermal sediments. *Microbiome* **5**, 106 (2017). 10.1186/s40168-017-0322-2 PMC5569505 28835260

[19] Dombrowski, N., Teske, A. P. & Baker, B. J. Expansive microbial metabolic versatility and biodiversity in dynamic Guaymas Basin hydrothermal sediments. *Nat. Commun.* **9**, 4999 (2018). 10.1038/s41467-018-07418-0 PMC6258724 30479325

[20] Galambos, D., Anderson, R. E., Reveillaud, J. & Huber, J. A. Genome-resolved metagenomics and metatranscriptomics reveal niche differentiation in functionally redundant microbial communities at deep-sea hydrothermal vents. *Environ. Microbiol.* **21**, 4395–4410 (2019). 10.1111/1462-2920.14806 PMC6899741 31573126

[21] Zhou, Z., St John, E., Anantharaman, K. & Reysenbach, A.-L. Global patterns of diversity and metabolism of microbial communities in deep-sea hydrothermal vent deposits. *Microbiome* **10**, 241 (2022). 10.1186/s40168-022-01424-7 PMC9793634 36572924

[22] Speth, D. R. et al. Microbial communities of Auka hydrothermal sediments shed light on vent biogeography and the evolutionary history of thermophily. *ISME J.* **16**, 1750–1764 (2022). 10.1038/s41396-022-01222-x PMC9213671 35352015

[23] Lloyd, K. G. et al. Predominant archaea in marine sediments degrade detrital proteins. *Nature* **496**, 215–218 (2013). 10.1038/nature12033 23535597

[24] Fortunato, C. S., Larson, B., Butterfield, D. A. & Huber, J. A. Spatially distinct, temporally stable microbial populations mediate biogeochemical cycling at and below the seafloor in hydrothermal vent fluids. *Environ. Microbiol.* **20**, 769–784 (2018). 10.1111/1462-2920.14011 29205750

[25] McGonigle, J. M., Lang, S. Q. & Brazelton, W. J. Genomic evidence for formate metabolism by *Chloroflexi* as the key to unlocking deep carbon in lost city microbial ecosystems. *Appl. Environ. Microbiol.* **86**, e02583–19 (2020). 10.1128/AEM.02583-19 PMC7117926 32033949

[26] Seitz, K. W., Lazar, C. S., Hinrichs, K.-U., Teske, A. P. & Baker, B. J. Genomic reconstruction of a novel, deeply branched sediment archaeal phylum with pathways for acetogenesis and sulfur reduction. *ISME J.* **10**, 1696–1705 (2016). 10.1038/ismej.2015.233 PMC4918440 26824177

[27] Zaremba-Niedzwiedzka, K. et al. Asgard archaea illuminate the origin of eukaryotic cellular complexity. *Nature* **541**, 353–358 (2017). 10.1038/nature21031 28077874

[28] Carr, S. A. et al. Carboxydotrophy potential of uncultivated Hydrothermarchaeota from the subseafloor crustal biosphere. *ISME J.* **13**, 1457–1468 (2019). 10.1038/s41396-019-0352-9 PMC6775978 30728468

[29] Anantharaman, K. et al. Thousands of microbial genomes shed light on interconnected biogeochemical processes in an aquifer system. *Nat. Commun.* **7**, 13219 (2016). 10.1038/ncomms13219 PMC5079060 27774985

[30] Dong, X. et al. A vast repertoire of secondary metabolites potentially influences community dynamics and biogeochemical processes in cold seeps. *Sci. Adv.* **10**, eadl2281 (2024). 10.1126/sciadv.adl2281 PMC11051675 38669328

[31] Paoli, L. et al. Biosynthetic potential of the global ocean microbiome. *Nature* **607**, 111–118 (2022). 10.1038/s41586-022-04862-3 PMC9259500 35732736

[32] Chen, R. et al. Discovery of an abundance of biosynthetic gene clusters in Shark bay microbial mats. *Front. Microbiol.* **11**, 1950 (2020). 10.3389/fmicb.2020.01950 PMC7472256 32973707

[33] Mara, P. et al. Metagenomic profiles of archaea and bacteria within thermal and geochemical gradients of the Guaymas Basin deep subsurface. *Nat. Commun.* **14**, 7768 (2023). 10.1038/s41467-023-43296-x PMC10681998 38012208

[34] Wei, B. et al. Global analysis of the biosynthetic chemical space of marine prokaryotes. *Microbiome* **11**, 144 (2023). 10.1186/s40168-023-01573-3 PMC10304266 37370187

[35] O’Brien, C. E. et al. Microbial biofilms associated with fluid chemistry and megafaunal colonization at post-eruptive deep-sea hydrothermal vents. *Deep Sea Res. Part II Top. Stud. Oceanogr.* **121**, 31–40 (2015).

[36] Qian, P.-Y., Cheng, A., Wang, R. & Zhang, R. Marine biofilms: diversity, interactions and biofouling. *Nat. Rev. Microbiol.* **20**, 671–684 (2022). 10.1038/s41579-022-00744-7 35614346

[37] Goel, N., Fatima, S. W., Kumar, S., Sinha, R. & Khare, S. K. Antimicrobial resistance in biofilms: exploring marine actinobacteria as a potential source of antibiotics and biofilm inhibitors. *Biotechnol. Rep.* **30**, e00613 (2021). 10.1016/j.btre.2021.e00613 PMC8105627 33996521

[38] Acinas, S. G. et al. Deep ocean metagenomes provide insight into the metabolic architecture of bathypelagic microbial communities. *Commun. Biol.* **4**, 604 (2021). 10.1038/s42003-021-02112-2 PMC8139981 34021239

[39] Vander Roost, J., Thorseth, I. H. & Dahle, H. Microbial analysis of zetaproteobacteria and co-colonizers of iron mats in the troll wall vent field, Arctic mid-ocean ridge. *PLoS One* **12**, e0185008 (2017). 10.1371/journal.pone.0185008 PMC5607188 28931087

[40] Liu, L.-J. et al. Physiology, taxonomy, and sulfur metabolism of the sulfolobales, an order of thermoacidophilic archaea. *Front. Microbiol.* **12**, 768283 (2021). 10.3389/fmicb.2021.768283 PMC8551704 34721370

[41] Qi, Y.-L. et al. Analysis of nearly 3000 archaeal genomes from terrestrial geothermal springs sheds light on interconnected biogeochemical processes. *Nat. Commun.* **15**, 4066 (2024). 10.1038/s41467-024-48498-5 PMC11094006 38744885

[42] Blin, K. et al. antiSMASH 6.0: improving cluster detection and comparison capabilities. *Nucleic Acids Res.* **49**, W29–W35 (2021). 10.1093/nar/gkab335 PMC8262755 33978755

[43] Johnston, I. et al. Identification of essential genes for Escherichia coli aryl polyene biosynthesis and function in biofilm formation. *Npj Biofilms Microbiomes* **7**, 56 (2021). 10.1038/s41522-021-00226-3 PMC8253772 34215744

[44] Hermann, L. et al. The ups and downs of ectoine: structural enzymology of a major microbial stress protectant and versatile nutrient. *Biol. Chem.* **401**, 1443–1468 (2020). 10.1515/hsz-2020-0223 32755967

[45] Lockwood, S., Greening, C., Baltar, F. & Morales, S. E. Global and seasonal variation of marine phosphonate metabolism. *ISME J.* **16**, 2198–2212 (2022). 10.1038/s41396-022-01266-z PMC9381506 35739297

[46] Shiner, E. K., Rumbaugh, K. P. & Williams, S. C. Interkingdom signaling: deciphering the language of acyl homoserine lactones. *FEMS Microbiol. Rev.* **29**, 935–947 (2005). 10.1016/j.femsre.2005.03.001 16219513

[47] Fuqua, C. & Greenberg, E. P. Listening in on bacteria: acyl-homoserine lactone signalling. *Nat. Rev. Mol. Cell Biol.* **3**, 685–695 (2002). 10.1038/nrm907 12209128

[48] Chen, Y. et al. The SCIFF-derived ranthipeptides participate in quorum sensing in solventogenic clostridia. *Biotechnol. J*. **15**, e2000136 (2020). 10.1002/biot.202000136 32713052

[49] Chan, D. C. K. & Burrows, L. L. Thiopeptides: antibiotics with unique chemical structures and diverse biological activities. *J. Antibiotics* **74**, 161–175 (2021). 10.1038/s41429-020-00387-x 33349675

[50] Ayikpoe, R. S. et al. A scalable platform to discover antimicrobials of ribosomal origin. *Nat. Commun.* **13**, 6135 (2022). 10.1038/s41467-022-33890-w PMC9576775 36253467

[51] Jangra, M. et al. A broad-spectrum lasso peptide antibiotic targeting the bacterial ribosome. *Nature* **640**, 1022–1030 (2025). 10.1038/s41586-025-08723-7 PMC12497486 40140562

[52] Benjdia, A. & Berteau, O. Radical SAM enzymes and ribosomally-synthesized and post-translationally modified peptides: a growing importance in the microbiomes. *Front. Chem*. **9**, 679965 (2021). 10.3389/fchem.2021.678068 PMC8326336 34350157

[53] Travin, D. Y. et al. Structure of ribosome-bound azole-modified peptide phazolicin rationalizes its species-specific mode of bacterial translation inhibition. *Nat. Commun*. **10**, 4563 (2019). 10.1038/s41467-019-12589-5 PMC6783444 31594941

[54] Bartholomae, M., Buivydas, A., Viel, J. H., Montalbán-López, M. & Kuipers, O. P. Major gene-regulatory mechanisms operating in ribosomally synthesized and post-translationally modified peptide (RiPP) biosynthesis. *Mol. Microbiol.* **106**, 186–206 (2017). 10.1111/mmi.13764 28787536

[55] Pfeiffer, I. P.-M., Schröder, M.-P. & Mordhorst, S. Opportunities and challenges of RiPP-based therapeutics. *Nat. Prod. Rep.* **41**, 990–1019 (2024). 10.1039/d3np00057e 38411278

[56] Robinson, S. L., Christenson, J. K. & Wackett, L. P. Biosynthesis and chemical diversity of β-lactone natural products. *Nat. Prod. Rep.* **36**, 458–475 (2019). 10.1039/c8np00052b 30191940

[57] Brady, S. F. & Clardy, J. Long-chain N-acyl amino acid antibiotics isolated from heterologously expressed environmental DNA. *J. Am. Chem. Soc.* **122**, 12903–12904 (2000).

[58] Romagnoli, C. et al. Antidermatophytic action of resorcinol derivatives: ultrastructural evidence of the activity of phenylethyl resorcinol against microsporum gypseum. *Molecules* **21**, 1306 (2016). 10.3390/molecules21101306 PMC6274034 27706019

[59] Schwarzer, D., Finking, R. & Marahiel, M. A. Nonribosomal peptides: from genes to products. *Nat. Prod. Rep*. **20**, 275 (2003). 10.1039/b111145k 12828367

[60] Peters, L. et al. Polyketide synthase-derived sphingolipids mediate microbiota protection against a bacterial pathogen in C. elegans. *Nat. Commun*. **16**, 5151 (2025). 10.1038/s41467-025-60234-1 PMC12134224 40461452

[61] Navarro-Muñoz, J. C. & Collemare, J. Evolutionary histories of type III polyketide synthases in fungi. *Front. Microbiol*. **10**, (2020). 10.3389/fmicb.2019.03018 PMC6985275 32038517

[62] Ortega, M. A. & van der Donk, W. A. New insights into the biosynthetic logic of ribosomally synthesized and post-translationally modified peptide natural products. *Cell Chem. Biol.* **23**, 31–44 (2016). 10.1016/j.chembiol.2015.11.012 PMC4779184 26933734

[63] Chowdhury, N. & Bagchi, A. Structural insight into the gene expression profiling of the hcn Operon in Pseudomonas aeruginosa. *Appl. Biochem. Biotechnol.* **182**, 1144–1157 (2017). 10.1007/s12010-016-2388-x 28064429

[64] Laville, J. et al. Characterization of the hcnABC Gene cluster encoding hydrogen cyanide synthase and anaerobic regulation by ANR in the strictly aerobic biocontrol agent Pseudomonas fluorescens CHA0. *J. Bacteriol.* **180**, 3187–3196 (1998). 10.1128/jb.180.12.3187-3196.1998 PMC107821 9620970

[65] Abramson, J. et al. Accurate structure prediction of biomolecular interactions with AlphaFold 3. *Nature* **630**, 493–500 (2024). 10.1038/s41586-024-07487-w PMC11168924 38718835

[66] Appler, K. E. et al. Oxygen metabolism in descendants of the archaeal-eukaryotic ancestor. *Nature*10.1038/s41586-026-10128-z (2026). 41708851

[67] Park, S. R., Park, J. W., Ban, Y. H., Sohng, J. K. & Yoon, Y. J. 2-Deoxystreptamine-containing aminoglycoside antibiotics: recent advances in the characterization and manipulation of their biosynthetic pathways. *Nat. Prod. Rep.* **30**, 11–20 (2013). 10.1039/c2np20092a 23179168

[68] Distler, J., Mansouri, K., Mayer, G., Stockmann, M. & Piepersberg, W. Streptomycin biosynthesis and its regulation in Streptomycetes. *Gene* **115**, 105–111 (1992). 10.1016/0378-1119(92)90547-3 1377151

[69] Pissowotzki, K., Mansouri, K. & Piepersberg, W. Genetics of streptomycin production in Streptomyces griseus: molecular structure and putative function of genes strELMB2N. *Mol. Gen. Genet. MGG* **231**, 113–123 (1991). 10.1007/BF00293829 1661369

[70] Ota, Y. et al. Butirosin-biosynthetic gene cluster from Bacillus circulans. *J. Antibiot.* **53**, 1158–1167 (2000). 10.7164/antibiotics.53.1158 11132962

[71] Pandey, A., Israr, J., Pandey, J. & Misra, S. Current approaches and implications in discovery of novel bioactive products from microbial sources. *Curr. Microbiol.* **82**, 258 (2025). 10.1007/s00284-025-04237-7 40263159

[72] Bhat, S. P. & Roach, D. J. Bioactivity of microbial biofilms in extreme environments. *Front. Microbiol.* **16**, 1602583 (2025). 10.3389/fmicb.2025.1602583 PMC12446376 40980321

[73] Waschulin, V. et al. Biosynthetic potential of uncultured Antarctic soil bacteria revealed through long-read metagenomic sequencing. *ISME J.* **16**, 101–111 (2022). 10.1038/s41396-021-01052-3 PMC8692599 34253854

[74] Medeiros, W. et al. Unlocking the biosynthetic potential and taxonomy of the Antarctic microbiome along temporal and spatial gradients. *Microbiol. Spectr.* **12**, e00244–24 (2024). 10.1128/spectrum.00244-24 PMC11237469 38747631

[75] Bagley, M. C., Dale, J. W., Merritt, E. A. & Xiong, X. Thiopeptide antibiotics. *Chem. Rev.* **105**, 685–714 (2005). 10.1021/cr0300441 15700961

[76] Vinogradov, A. A. & Suga, H. Introduction to thiopeptides: biological activity, biosynthesis, and strategies for functional reprogramming. *Cell Chem. Biol.* **27**, 1032–1051 (2020). 10.1016/j.chembiol.2020.07.003 32698017

[77] Duarte, A. F. D. S. et al. Hyicin 4244, the first sactibiotic described in staphylococci, exhibits an anti-staphylococcal biofilm activity. *Int. J. Antimicrob. Agents* **51**, 349–356 (2018). 10.1016/j.ijantimicag.2017.06.025 28705677

[78] Jia, Y., Han, Y., Liu, X. & Zhang, Q. Synergistic action of two radical SAM enzymes in the biosynthesis of thuricin CD, a two-component sactibiotic. *Chem. Sci.* **16**, 10722–10730 (2025). 10.1039/d5sc01546d PMC12093266 40406209

[79] Hudson, G. A. & Mitchell, D. A. RiPP antibiotics: biosynthesis and engineering potential. *Curr. Opin. Microbiol.* **45**, 61–69 (2018). 10.1016/j.mib.2019.06.001 31303539

[80] Liu, A. et al. Functional elucidation of TfuA in peptide backbone thioamidation. *Nat. Chem. Biol.* **17**, 585–592 (2021). 10.1038/s41589-021-00771-0 PMC8084935 33707784

[81] Song, Z. et al. Decoding the chemical language of ribosomally synthesized and post-translationally modified peptides from the untapped archaea domain. *Angew. Chem. Int. Ed*. **64**, e202501074 (2025). 10.1002/anie.202501074 PMC12144867 40180612

[82] Wei, X. et al. Exploring and expanding the natural chemical space of bacterial diterpenes. *Nat. Commun*. **16**, 3721 (2025). 10.1038/s41467-025-57145-6 PMC12009305 40253449

[83] Díaz-Rueda, P., Morales De Los Ríos, L., Romero, L. C. & García, I. Old poisons, new signaling molecules: the case of hydrogen cyanide. *J. Exp. Bot.* **74**, 6040–6051 (2023). 10.1093/jxb/erad317 PMC10575699 37586035

[84] Anand, A. et al. Biological hydrogen cyanide emission globally impacts the physiology of both HCN-emitting and HCN-perceiving *Pseudomonas*. *mBio* **14**, e00857-23 (2023). 10.1128/mbio.00857-23 PMC10653877 37650608

[85] Rojas-Coll, A., Valencia, J.-I., Tognarelli, J. & Fernández-Bunster, G. Genomic-driven identification of conserved biosynthetic gene clusters in cladosporium limoniforme: the case of the DHN-melanin pathway. *Metabolites* **16**, 77 (2026). 10.3390/metabo16010077 PMC12843665 41590685

[86] A. Marques, A. F. et al. The seven sisters hydrothermal system: first record of shallow hybrid mineralization hosted in mafic volcaniclasts on the Arctic mid-ocean ridge. *Minerals* **10**, 439 (2020).

[87] Steen, I. H. et al. Novel barite chimneys at the Loki’s castle vent field shed light on key factors shaping microbial communities and functions in hydrothermal systems. *Front. Microbiol*. **6**, 1510 (2016). 10.3389/fmicb.2015.01510 PMC4703759 26779165

[88] Boonnawa, C. et al. Diverse styles of hydrothermal chemistry on the ultraslow Arctic Mohns Ridge: the Fåvne, Ægir and Loki’s Castle vent fields. *AGU Fall Meet. Abstr.* **12**, OS55B–02 (2022).

[89] Chen, S. Ultrafast one-pass FASTQ data preprocessing, quality control, and deduplication using fastp. *iMeta* **2**, e107 (2023). 10.1002/imt2.107 PMC10989850 38868435

[90] Li, D., Liu, C.-M., Luo, R., Sadakane, K. & Lam, T.-W. MEGAHIT: an ultra-fast single-node solution for large and complex metagenomics assembly via succinct *de Bruijn* graph. *Bioinformatics* **31**, 1674–1676 (2015). 10.1093/bioinformatics/btv033 25609793

[91] Uritskiy, G. V., DiRuggiero, J. & Taylor, J. MetaWRAP—a flexible pipeline for genome-resolved metagenomic data analysis. *Microbiome* **6**, 158 (2018). 10.1186/s40168-018-0541-1 PMC6138922 30219103

[92] Wu, Y.-W., Simmons, B. A. & Singer, S. W. MaxBin 2.0: an automated binning algorithm to recover genomes from multiple metagenomic datasets. *Bioinformatics* **32**, 605–607 (2016). 10.1093/bioinformatics/btv638 26515820

[93] Alneberg, J. et al. Binning metagenomic contigs by coverage and composition. *Nat. Methods* **11**, 1144–1146 (2014). 10.1038/nmeth.3103 25218180

[94] Kang, D. D. et al. MetaBAT 2: an adaptive binning algorithm for robust and efficient genome reconstruction from metagenome assemblies. *PeerJ* **7**, e7359 (2019). 10.7717/peerj.7359 PMC6662567 31388474

[95] Chklovski, A., Parks, D. H., Woodcroft, B. J. & Tyson, G. W. CheckM2: a rapid, scalable and accurate tool for assessing microbial genome quality using machine learning. *Nat. Methods* **20**, 1203–1212 (2023). 10.1038/s41592-023-01940-w 37500759

[96] Seemann, T. Barrnap 0.9: Rapid ribosomal RNA prediction. *RNA* **30**, 1264–1276 (2018).

[97] Chan, P. P., Lin, B. Y., Mak, A. J. & Lowe, T. M. tRNAscan-SE 2.0: improved detection and functional classification of transfer RNA genes. *Nucleic Acids Res.* **49**, 9077–9096 (2021). 10.1093/nar/gkab688 PMC8450103 34417604

[98] *The Genome Standards Consortium* et al. Minimum information about a single amplified genome (MISAG) and a metagenome-assembled genome (MIMAG) of bacteria and archaea. *Nat. Biotechnol*. **35**, 725–731 (2017). 10.1038/nbt.3893 PMC6436528 28787424

[99] Camargo, A. P. et al. Plant microbiomes harbor potential to promote nutrient turnover in impoverished substrates of a Brazilian biodiversity hotspot. *ISME J.* **17**, 354–370 (2023). 10.1038/s41396-022-01345-1 PMC9938248 36536072

[100] Olm, M. R., Brown, C. T., Brooks, B. & Banfield, J. F. dRep: a tool for fast and accurate genomic comparisons that enables improved genome recovery from metagenomes through de-replication. *ISME J.* **11**, 2864–2868 (2017). 10.1038/ismej.2017.126 PMC5702732 28742071

[101] Delmont, T. O. et al. Nitrogen-fixing populations of Planctomycetes and Proteobacteria are abundant in surface ocean metagenomes. *Nat. Microbiol.* **3**, 804–813 (2018). 10.1038/s41564-018-0176-9 PMC6792437 29891866

[102] Parks, D. H. et al. GTDB: an ongoing census of bacterial and archaeal diversity through a phylogenetically consistent, rank normalized and complete genome-based taxonomy. *Nucleic Acids Res.* **50**, D785–D794 (2022). 10.1093/nar/gkab776 PMC8728215 34520557

[103] Chaumeil, P.-A., Mussig, A. J., Hugenholtz, P. & Parks, D. H. GTDB-Tk: a toolkit to classify genomes with the Genome Taxonomy Database. *Bioinformatics* **36**, 1925–1927 (2020). 10.1093/bioinformatics/btz848 PMC7703759 31730192

[104] Chaumeil, P.-A., Mussig, A. J., Hugenholtz, P. & Parks, D. H. GTDB-Tk v2: memory friendly classification with the genome taxonomy database. *Bioinformatics* **38**, 5315–5316 (2022). 10.1093/bioinformatics/btac672 PMC9710552 36218463

[105] Seemann, T. Prokka: rapid prokaryotic genome annotation. *Bioinformatics* **30**, 2068–2069 (2014). 10.1093/bioinformatics/btu153 24642063

[106] Blin, K. et al. antiSMASH 7.0: new and improved predictions for detection, regulation, chemical structures and visualisation. *Nucleic Acids Res.* **51**, W46–W50 (2023). 10.1093/nar/gkad344 PMC10320115 37140036

[107] Kautsar, S. A., van der Hooft, J. J. J., de Ridder, D. & Medema, M. H. BiG-SLiCE: a highly scalable tool maps the diversity of 1.2 million biosynthetic gene clusters. *GigaScience* **10**, giaa154 (2021). 10.1093/gigascience/giaa154 PMC7804863 33438731

[108] Navarro-Muñoz, J. C. et al. A computational framework to explore large-scale biosynthetic diversity. *Nat. Chem. Biol.* **16**, 60–68 (2020). 10.1038/s41589-019-0400-9 PMC6917865 31768033

[109] Terlouw, B. R. et al. MIBiG 3.0: a community-driven effort to annotate experimentally validated biosynthetic gene clusters. *Nucleic Acids Res.* **51**, D603–D610 (2023). 10.1093/nar/gkac1049 PMC9825592 36399496

[110] Smoot, M. E., Ono, K., Ruscheinski, J., Wang, P.-L. & Ideker, T. Cytoscape 2.8: new features for data integration and network visualization. *Bioinformatics* **27**, 431–432 (2011). 10.1093/bioinformatics/btq675 PMC3031041 21149340

[111] Kopylova, E., Noé, L. & Touzet, H. SortMeRNA: fast and accurate filtering of ribosomal RNAs in metatranscriptomic data. *Bioinformatics* **28**, 3211–3217 (2012). 10.1093/bioinformatics/bts611 23071270

[112] Pascal Andreu, V. et al. BiG-MAP: an automated pipeline to profile metabolic gene cluster abundance and expression in microbiomes. *mSystems* **6**, 10.1128/msystems.00937-21 (2021). PMC8547482 34581602

[113] Li, H. Minimap2: pairwise alignment for nucleotide sequences. *Bioinformatics* **34**, 3094–3100 (2018). 10.1093/bioinformatics/bty191 PMC6137996 29750242

[114] Li, H. New strategies to improve minimap2 alignment accuracy. *Bioinformatics* **37**, 4572–4574 (2021). 10.1093/bioinformatics/btab705 PMC8652018 34623391

[115] Mauri, M., Elli, T., Caviglia, G., Uboldi, G. & Azzi, M. RAWGraphs: a visualisation platform to create open outputs. in *Proceedings of the 12th Biannual Conference on Italian SIGCHI Chapter* 1–5 10.1145/3125571.3125585 (ACM, 2017)

[116] Thuc Nguyen. trongthucnguyen/DeepSeaQuence_biofilms: Source data and analysis scripts for: Diverse Biosynthetic Pathways in Arctic Hydrothermal Biofilms. *Zenodo* 10.5281/ZENODO.20057600 (2026). PMC13635244 42830300

[117] Goddard, T. D. et al. UCSF ChimeraX: Meeting modern challenges in visualization and analysis. *Protein Sci.* **27**, 14–25 (2018). 10.1002/pro.3235 PMC5734306 28710774

[118] Meng, E. C. et al. UCSF ChimeraX: tools for structure building and analysis. *Protein Sci.* **32**, e4792 (2023). 10.1002/pro.4792 PMC10588335 37774136

[119] Pettersen, E. F. et al. UCSF ChimeraX: structure visualization for researchers, educators, and developers. *Protein Sci.* **30**, 70–82 (2021). 10.1002/pro.3943 PMC7737788 32881101

