# Supplementary material for: Diverse biosynthetic pathways in Arctic hydrothermal biofilms
Source: Nat Commun. 2026 Sep 3;17:10469. doi: 10.1038/s41467-026-77384-5 (PMC13635244; doi:10.1038/s41467-026-77384-5)
Supplement: Supplementary file 1 [file 41467_2026_77384_MOESM11_ESM.pdf]

nature portfolio

Corresponding author(s): Runar Stokke

Last updated by author(s): May 5, 2026

Reporting Summary

Nature Portfolio wishes to improve the reproducibility of the work that we publish. This form provides structure for consistency and transparency in reporting. For further information on Nature Portfolio policies, see our Editorial Policies and the Editorial Policy Checklist.

Statistics

For all statistical analyses, confirm that the following items are present in the figure legend, table legend, main text, or Methods section.

n/a	Confirmed	
☐	☒	The exact sample size (*n*) for each experimental group/condition, given as a discrete number and unit of measurement
☐	☒	A statement on whether measurements were taken from distinct samples or whether the same sample was measured repeatedly
☐	☒	The statistical test(s) used AND whether they are one- or two-sided<br>*Only common tests should be described solely by name; describe more complex techniques in the Methods section.*
☒	☐	A description of all covariates tested
☐	☒	A description of any assumptions or corrections, such as tests of normality and adjustment for multiple comparisons
☐	☒	A full description of the statistical parameters including central tendency (e.g. means) or other basic estimates (e.g. regression coefficient) AND variation (e.g. standard deviation) or associated estimates of uncertainty (e.g. confidence intervals)
☐	☒	For null hypothesis testing, the test statistic (e.g. *F*, *t*, *r*) with confidence intervals, effect sizes, degrees of freedom and *P* value noted<br>*Give P values as exact values whenever suitable.*
☒	☐	For Bayesian analysis, information on the choice of priors and Markov chain Monte Carlo settings
☒	☐	For hierarchical and complex designs, identification of the appropriate level for tests and full reporting of outcomes
☒	☐	Estimates of effect sizes (e.g. Cohen's *d*, Pearson's *r*), indicating how they were calculated

Our web collection on statistics for biologists contains articles on many of the points above.

Software and code

Policy information about availability of computer code

Data collection

Data collected for DNA sequencing aquired via the Norwegian Sequencing Centre (University of Oslo, Norway) . Data for cDNA sequencing using an in-house MinION Mk1C device (Oxford Nanopore Technologies plc, UK).

Data analysis

Metagenomics: Quality, duplication rate, and adapter contamination of Illumina NovaSeq S4 150bp paired-end using fastp (v0.23.2). Metagenomic assembly into contigs by MEGAHIT (v1.2.9). MAG reconstruction using metaWRAP (v1.3.2) which implements the combinatorial use of MaxBin2 (v2.2.6) , CONCOCT (v1.0.0), and MetaBat2 (v2.12.1). Quality of each MAG was estimated with CheckM1 (v1.0.12) and CheckM2 (1.1.0) in addition to the presence/absence of rRNA and tRNA determined by barrnap v0.9 and tRNAscan v2.0.12, respectively. . De-replication uisng dRep (v3.4.0) and abundance estimates using CoverM (v.0.6.1). Taxonomic classification was performed using the Genome Taxonomy Database via the Genome Taxonomy Database Toolkit (GTDB-Tk, v2.5.2 – GTDB R226 taxonomy).
Metatranscriptomics: A detailed workflow of the RNA work is uploaded to protocols.io for repoducibility: http://doi.org/10.17504/protocols.io.dm6gpzz9plzp/v1. Nanopore sequencing reads were basecalled using Dorado (version 0.5.1). Ribosomal RNA sequences were filtered out using SortMeRNA (v.4.3.6). cDNA sequences were mapped against their corresponding metagenomic datasets using BiG-MAP (v.1.0.0).

Gene annotation of MAGs was performed using the Prokka annotation pipeline (v.1.14.6). Secondary metabolite BGCs were identified and analyzed using antiSMASH (v.7.0). Predicted BGCs were subjected to downstream analysis with BiG-SLiCE (v.2.0.0). Network analysis of recovered BGCs was performed using BiG-SCAPE (v1.1.5). Resulting sequence similarity matrices were visualized using Cytoscape (v.3.10.3).

All statistical analyses were conducted using Python (version 3.13.2); principal Component Analysis (PCA) of microbial communities across biofilm samples was performed using the scikit-learn package (v.1.6.1), Bray-Curtis dissimilarities based on relative abundances of MAGs were calculated using the pdist function from the scipy package (v.1.15.2). Pairwise community similarity based on microbial order presence-

absence data was quantified using the Dice–Sørensen coefficient. Statistical significance of the observed similarities was assessed using a permutation test (1,000 permutations per pair). Kruskal-Wallis tests comparing the expression of different BGC classes were performed using the kruskal function from the scipy package. Post-hoc Dunn's tests to identify significant pairwise differences among groups were conducted with the scikit-posthocs package (v.0.11.4), using TPM for each BGC class across samples. Visualisations were created using RAWGraphs (v.2.0.0) and Python's Matplotlib library (v.3.10.1). Source code and data for all custom statistical analyses is available on GitHub (https://github.com/trongthucnguyen/DeepSeaQuence_biofilms).

For manuscripts utilizing custom algorithms or software that are central to the research but not yet described in published literature, software must be made available to editors and reviewers. We strongly encourage code deposition in a community repository (e.g. GitHub). See the Nature Portfolio guidelines for submitting code & software for further information.

Data

Policy information about availability of data

All manuscripts must include a data availability statement. This statement should provide the following information, where applicable:

- Accession codes, unique identifiers, or web links for publicly available datasets
- A description of any restrictions on data availability
- For clinical datasets or third party data, please ensure that the statement adheres to our policy

Data availability
Metadata of all samples and metagenomes in the study is deposited at NCBI under BioProjects listed in Table 1. Sequence data of individual MAGs has been deposited under the respective BioSample accession numbers listed in Supplementary Table 3, including genome accession numbers, and all raw sequencing read data has been deposited to the Sequence Read Archive (SRA) with the following accession numbers: SRR36181650 (M24), SRR36183670 (M25), SRR36184633 (M26), SRR36184876 (M33), SRR36184925 (M36), SRR36185683 (M37), SRR36199626 (M38), SRR36199942 (M39) and SRR36203984 (M42). Nanopore cDNA raw reads have been deposited to SRA under accession numbers: SRR36202719 (c24), SRR36203181 (c25), SRR36217904 (c33), SRR36218254 (c36), SRR36218304 (c37), SRR36218718 (c38), SRR36218944 (c42) . A FigShare project (https://doi.org/10.6084/m9.figshare.c.8159183) has been established for the study which includes GenBank and index files of all identified BGCs, in addition to the network file generated from BiG-SCAPE (https://doi.org/10.6084/m9.figshare.30762029), a protein sequence file of the hcnAB and hcnC from the Heimdallarchaeion Aegir_M39_B184 (https://doi.org/10.6084/m9.figshare.30788630), and a figure of the superimposed AlphaFold3 models of the hcnC from the Heimdallarchaeion Aegir_M39_B184 and Pseudomonas fluorescence PS619 (https://doi.org/10.6084/m9.figshare.30788579), in addition to a clinker result file (https://doi.org/10.6084/m9.figshare.31948944).

Code availability
The source code and data for all statistical analyses is available on GitHub (https://github.com/trongthucnguyen/DeepSeaQuence_biofilms).

Research involving human participants, their data, or biological material

Policy information about studies with human participants or human data. See also policy information about sex, gender (identity/presentation), and sexual orientation and race, ethnicity and racism.

Reporting on sex and gender	NA
Reporting on race, ethnicity, or other socially relevant groupings	NA
Population characteristics	NA
Recruitment	NA
Ethics oversight	NA

Note that full information on the approval of the study protocol must also be provided in the manuscript.

Field-specific reporting

Please select the one below that is the best fit for your research. If you are not sure, read the appropriate sections before making your selection.

☐ Life sciences ☐ Behavioural & social sciences ☒ Ecological, evolutionary & environmental sciences

For a reference copy of the document with all sections, see nature.com/documents/nr-reporting-summary-flat.pdf

Ecological, evolutionary & environmental sciences study design

All studies must disclose on these points even when the disclosure is negative.

Study description	In this study, we explore the biodiversity and biosynthetic potential of biofilm microbial communities from the Loki's Castle, Aegir's Vent Field, and Seven Sisters vent field along the Arctic Mid-Ocean Ridges. By integrating Nanopore long-read metatranscriptomic data with biosynthetic gene cluster (BGC) predictions from metagenome-assembled genomes, we assess the transcriptional activity and site-specific expression profiles of BGCs across distinct vent ecosystems.

Research sample	Biofilms growing on the outside of venting hydrothermal features. Biofilm samples were collected at three hydrothermal locations, both from shallow water and deep-sea, along the AMOR. In the current study 9 microbial biofilms were collected, one from the shallow water Seven Sisters vent field at 130 mbsl, one from the diffuse venting at the Loki's Castle vent field (Barite field) situated at 2390 mbsl , and seven samples from the Aegir vent field at 2200 mbsl
Sampling strategy	The accessibility and cost of performing deep-sea sampling did not allow for multiple biofilm samples from each site, hence 9 individual biofilms from 9 unique sampling sites were used in the study. Samples were collected using the Ægir6000 ROV on board the R/V G.O. SARS, equipped with a biosyringe (a hydraulic sampling cylinder) connected to the ROV as part of the research program at Centre for Deep-Sea Research. Sampling of biofilms was coordinated by authors Stokke and Steen, in addition to author Nguyen in 2022. Previous experience working with such biofilms concerning biomass was used to estimate the sequencing amount needed to cover the biodiversity of the samples, hence multiplexing using a150bp paired-end Illumina libraries on a NovaSeq S4 flow cell was used. RNA from 7 of the 9 biofilms were successfully extracted and sequenced as cDNA using an in-house MinION Nanopore sequencer.
Data collection	Laboratory data collection was carried out at University of Bergen by TTN, IHS and RS.
Timing and spatial scale	The sampling was performed during 4 research expeditions to the Arctic Mid-Ocean Ridges the Summer months of June/July in 2017, 2020, 2021 and 2022. To ensure a quick and timely treatment of the samples at sea, samples retrieved onboard R/V G.O. SARS were centrifuged immediately arriving on deck and the supernatant were removed before frozen in liquid nitrogen and stored at -80°C until processing.
Data exclusions	Two RNA samples from GS20-ROV9-BS2 and GS21-106-ROV435A-BS were excluded from the study due to low RNA output. In addition, ribosomal reads filtered during the metatranscriptomic workflow were removed to retain putative mRNA reads for further analaysis.
Reproducibility	To ensure the reproducibility of the metatranscriptomic pipeline used in the study, the full protocol is made available to protocols.io: http://doi.org/10.17504/protocols.io.dm6gpzz9plzp/v1.
Randomization	The advanced nature and cost of deep-sea sampling imapct the ability to perform large scale sampling, reducing the number of replicates. Hence, randomization was not used/applicable for this study.
Blinding	This is not relevant to the current study.

Did the study involve field work? ☒ Yes ☐ No

Field work, collection and transport

Field conditions	The site of sampling was both shallow- and deep-sea locations along the Arctic Mid-Ocean Ridges with defined geochemical and geological setting. The sampling was conducted in open sea using a ROV onboard the research wessel G.O SARS in June/July of 2017, 2020, 2021 and 2022.
Location	Sampling was conducted at three hydrothermal locations: Loki's Castle vent field (73.56 N 8.16 E, 2390 mbsl), Aegir 's vent field (72.32 N 1.51 E, 2300 mbsl), and Seven Sisters vent field (71.14 N 12.79 W, 165 mbsl)
Access & import/export	Access to the hydrothermal habitats along AMOR was achieved as part of the sampling campaigns at the Centre for Deep-Sea Research at the University of Bergen, in full compliance with Norwegian authorities. The sampling sites along AMOR are all within the Norwegian Economical Exclusive Zone (EEZ).
Disturbance	Sampling in these pristine and vulnerable deep-sea habitats was carefully designed to minimize ecological disturbance. This was achieved by deploying a remotely operated vehicle (ROV) equipped with precision scientific instruments, allowing for targeted, non-invasive exploration.

Reporting for specific materials, systems and methods

We require information from authors about some types of materials, experimental systems and methods used in many studies. Here, indicate whether each material, system or method listed is relevant to your study. If you are not sure if a list item applies to your research, read the appropriate section before selecting a response.

Materials & experimental systems

n/a	Involved in the study
☒	☐ Antibodies
☒	☐ Eukaryotic cell lines
☒	☐ Palaeontology and archaeology
☒	☐ Animals and other organisms
☒	☐ Clinical data
☒	☐ Dual use research of concern
☒	☐ Plants

Methods

n/a	Involved in the study
☒	☐ ChIP-seq
☒	☐ Flow cytometry
☒	☐ MRI-based neuroimaging

Plants

Seed stocks	*Report on the source of all seed stocks or other plant material used. If applicable, state the seed stock centre and catalogue number. If plant specimens were collected from the field, describe the collection location, date and sampling procedures.*
Novel plant genotypes	*Describe the methods by which all novel plant genotypes were produced. This includes those generated by transgenic approaches, gene editing, chemical/radiation-based mutagenesis and hybridization. For transgenic lines, describe the transformation method, the number of independent lines analyzed and the generation upon which experiments were performed. For gene-edited lines, describe the editor used, the endogenous sequence targeted for editing, the targeting guide RNA sequence (if applicable) and how the editor was applied.*
Authentication	*Describe any authentication procedures for each seed stock used or novel genotype generated. Describe any experiments used to assess the effect of a mutation and, where applicable, how potential secondary effects (e.g. second site T-DNA insertions, mosiacism, off-target gene editing) were examined.*
