# Supplementary material for: Diverse biosynthetic pathways in Arctic hydrothermal biofilms
Source: Nat Commun. 2026 Sep 3;17:10469. doi: 10.1038/s41467-026-77384-5 (PMC13635244; doi:10.1038/s41467-026-77384-5)
Supplement: Supplementary file 2 [file 41467_2026_77384_MOESM12_ESM.pdf]

nature portfolio

Peer Review File

Diverse Biosynthetic Pathways in Arctic hydrothermal biofilms

Corresponding Author: Dr Runar Stokke

This file contains all reviewer reports in order by version, followed by all author rebuttals in order by version.

Attachments originally included by the reviewers as part of their assessment can be found at the end of this file.

Version 0:

Reviewer comments:

Reviewer #1

(Remarks to the Author)
This manuscript uses MAGs and long read nanopore RNA sequencing from 9 samples collected from 3 vent fields in the Arctic, to explore the diversity of biosynthetic gene clusters that encode for secondary metabolites. They show that the ribosomally synthesized and post translationally modified peptides are the more prevalent and also the most prevalently expressed across the samples they studied. They also show that some archaea have hydrogen cyanide synthesis pathways.
The strength in this paper is that they authors explore these BGCs specifically. And they show they are transcribed. However, the manuscript shows a severe lack of understanding of the ecology of deep-sea hydrothermal vent microbiomes. There is a lot of interesting work done even using clone libraries and amplicon sequencing of the patterns of microbial diversity from global deep-sea hydrothermal systems. Further, although authors report numerous MAGs from the Sulfolobales, they clearly do not have a good understanding of the revised taxonomy of this group, as these are not restricted to acidic environments as classified now by GTDB. And since the authors use this for classification, they may want to review this taxonomy.
The authors also state (line 265) that there is intense competition. Not sure where this has been shown scientifically, but of course, any biofim there is competition.
The authors state that (line 56) biofilms remain largely unexplored. The bacterial mats the hydrothermal deposits etc. are all biofilms. Some of the samples the authors studied ‘look' like hydrothermal deposits.
If these are a ‘unique' and remarkable' reservoir of unique bioactive compounds, the authors do not provide compelling evidence to this point. They do not compare they data analytically to other studies of MAGs from deep-sea vents. They do state others have shown such novel secondary metabolites, many with antimicrobial activities' (line 55) so how does this study compare? Do the authors see any of the lysozyme antimicrobials reported by Metcalf et al. from a deep-sea vent organism?
Minor issues.
Some references are cited twice. E.g. # 7 and 14 are the same references
There is no consistency in the figures with regard to sites. It would be great if the sites are always represented in the same order
Line 423. It appears this sectin is in the wrong place?
With MAGs, its better to refer to genes as being undetected, rather than absent. Ie. They just were not sequenced, so not detected. Maybe they are absent, but with incomplete genomes its misleading to consider genes absent.
Line 379. If authors are using the Bowers et al ref as they state, then their nearly complete genomes are actually the high quality ‘ MAGs'.

Be consistent with acronyms. Not sure AVF was ever defined.

(Remarks on code availability)
The code worked flawlessly

Reviewer #2

(Remarks to the Author)
The manuscript “Remarkable biosynthetic capacity of Arctic hydrothermal biofilms” reports a metagenomic and metatranscriptomic analysis of microbial biofilms from Arctic hydrothermal vents. The authors identify over 1,000 bacterial and archaeal MAGs encoding nearly 3,000 biosynthetic gene clusters (BGCs), highlighting RiPPs as dominant and suggesting novel findings such as a MAG with 107 BGCs and the first evidence of a hydrogen cyanide biosynthetic pathway in Archaea.

The paper presents exciting and potentially groundbreaking discoveries about the biosynthetic diversity of hydrothermal vent microbiomes. However, the lack of publicly available data and insufficient methodological transparency make it impossible to verify the major claims. Several significant concerns arise regarding data reproducibility, potential MAG contamination, and the robustness of key findings.

Data Availability and Reproducibility
The two central claims of the manuscript—(i) the discovery of a MAG with exceptionally high biosynthetic potential and (ii) the presence of an HCN pathway in Archaea—are highly significant and challenge long-standing assumptions. However, the absence of accessible raw data prevents validation. The authors mention deposition of raw data under a BioProject, but it appears to be private. No assemblies, MAGs, or BGC sequences are deposited, and the authors only offer to share data upon request. Public availability is essential for reproducibility.

MAG with 107 BGCs (Lines 171–175)
Reporting 107 BGCs in a 6.7 Mbp MAG is extraordinary. The highest number of BGCs in antiSMASH-DB is 55, in a 12 Mbp genome. This raises concerns about contamination or binning artifacts, possibly from other Pseudomonas spp., which dominate the dataset. Although CheckM reports low contamination, its reliance on single-copy marker genes means it may miss contamination in BGC-rich regions. Alternatively, fragmented BGCs might have been counted multiple times, inflating the total.

HCN Pathway in Archaea (Lines 237–245)
The authors claim the first report of an archaeal HCN pathway, previously known from Pseudomonas. This could also result from contamination, as the MAG’s taxonomic assignment is based on the overall bin rather than the contig harboring the pathway. Figure 4’s antiSMASH comparison does show a related cluster in another archaeon, which supports the possibility but warrants further verification. It remains unclear whether the reported two-gene operon is incomplete (missing hcnC). Similar caution applies to the reported aminoglycoside pathway (Lines 256–262).

Interpretation of MAG Abundance (Lines 100, 118–136)
The authors equate the number of MAGs assigned to a taxon with its abundance in the community. This is not necessarily correct; the number of recovered MAGs depends on assembly and binning success rather than true abundance.

Methodological Concerns

Genome quality thresholds (Lines 377–382): The authors apply non-standard definitions diverging from MIMAG; what they term “near-complete” corresponds to MIMAG HQ, and rRNA/tRNA presence is not considered.

Dereplication (Lines 383–388): An ANI threshold of 98 % is unusually stringent. While acceptable, comparisons to other studies must note this difference.

antiSMASH Version (Line 409): Inconsistent reporting (v6 vs v7).

BigMAP Analysis (Lines 427–430): Bowtie is not suitable for long-read data due to its error profiles and read length limitations; it may bias RPKM/TPM normalization. A long-read-aware alignment and normalization to housekeeping genes would yield more reliable abundance estimates. Differences in RNA depletion across samples further complicate comparisons.

Additional Comments

Abstract (Line 20): “Antimicrobial potential” is mentioned but never discussed or supported in the manuscript.

Introduction (Lines 44–47): Missing citation.

Results (Lines 202–215): Not all reported metabolites are necessarily antimicrobial; the wording should be refined.

Results (Lines 221–224): Reporting raw BGC counts without clustering into GCFs (and distinguishing from MIBiG entries) limits interpretability.

Discussion (Lines 281–282): The cited study reports similar numbers but under different size thresholds (>10 kbp); comparisons should be made carefully.

In summary, this study tackles important and potentially paradigm-shifting questions but currently lacks sufficient data

transparency and methodological rigor to support its conclusions. Public release of raw data, MAG assemblies, and BGC sequences—as well as additional validation of key MAGs—is essential before the manuscript can be adequately evaluated.

(Remarks on code availability)

Version 1:

Reviewer comments:

Reviewer #3

(Remarks to the Author)
A few minor comments on the revised manuscript.
- Line 20-23: Is it non-ribosomal peptide synthetases that show antimicrobial activity? The way it is written it implies it is the synthetases are tested for microbial activity, change to reflect that they are observed to be expressed.
- line 79: no reference or spelling out MIBiG at the first occurrence.
- Line 103: '>2000 bp' needs to be put in context 'minimum contig size in MAGs is >200bp' or similar.
-Line 120: Add in that between 20 and 75.3% of the community was presented by the ~1140 MAGs in this study to give the readers a sense of what % MAGs the BGCs represent.
- Line 147: is the Dice-Sorensen Coefficient? I didn't think there was a Sorensen p-value. also, not obvious in statistical tests in method section.
- Line 189: 'adaptation' to what?
- Line 244: was or were?
- Line 262: Prometheoarchaeum syntrophicum should be unitalicized, Candidatus is italicised.
- Lines 397-402: Need to include a 'Contig Number' column for each MAG, this gives the reader a sense of the quality more so than N50.
- Lines 406-407: Do the >90%, <5% medium quality genomes have 5,16,23S rRNA genes,>18 tRNAs like the high-quality genomes? it needs to be at least stated as to their criteria, if not consistent between the two categories.

(Remarks on code availability)
I assume that the initial reviewers did this.

Reviewer #4

(Remarks to the Author)

(Remarks on code availability)
The code is available and includes both a README and a requirements file for installation and use.

Reviewer #5

(Remarks to the Author)
NCOMMS-25-73582A

Remarkable biosynthetic capacity of Arctic hydrothermal biofilms

The manuscript is an interesting and important metagenomic assessment of biosynthetic capacity across 9 samples from 3 vent fields. Notably, the authors have also performed ONT long-read RNA analysis alongside the eDNA sequencing, which adds a functional component to the study and is one of the manuscript’s main strengths, alongside the interesting and unique environment being studied. My role was primarily to check that the previous reviewer's comments were addressed sufficiently. After reading the revised manuscript and Reviewer #2’s comments, I still have concerns about limitations of the data and the strength of some claims. In particular, there is insufficient discussion of the limitations of assessing biosynthetic capability when almost 90% of antiSMASH regions are fragmented, which adds significant caveats to the findings presented. This especially affects (i) the reported number of gene clusters (inflated due to fragmentation) and (ii) the dominance of RiPPs (the easiest BGC type to detect on short contigs). Overall, I think the manuscript is well written and a notable contribution on an interesting microbial ecosystem, but this limitation needs to be addressed more clearly.

Major concerns:

A) Fragmented MAGS with short contigs and misinterpretation

There is an acknowledgement that only ~12% of BGCs are complete, but many results are still reported as if fragmentation has minimal impact. The implications are significant for the interpretation of results:

1) Number of BGCs: Antismash reports ‘regions’ of the genome that hit specific pHMM rules. These ‘regions’ are not definitely biosynthetic gene clusters. As evidenced by Barite_M33_B89 many of these ‘BGCs’ are in fact just fragments of NRPS genes that happen to contain an A, PCP and C domain: https://antismash.secondarymetabolites.org/upload/bacteria-

2086baaa-5f42-4faa-a878-3b3157275e17/index.html#r4c1. Given the typical size of NRPS BGCs, what would have been a single NRPS cluster spanning perhaps 120–200 kb can appear as 10–15 “BGCs” in fragmented data (e.g. B89 has 47 regions <5 kb). This is an artefact of the fragmented sequencing/assembly, not the biological reality. Reporting that Barite_M33_B89 “has 107 BGCs” is therefore misleading: antiSMASH detected 107 regions/contigs containing core biosynthetic domains.

2) Type of BGCs: fragmentation biases what you are more likely to detect. For example, NRPS-like regions can be over-called because only partial NRPS domain content is recovered before a contig break. Moreover, RiPPs (often among the shortest genes that trigger antiSMASH rules) will be much easier to detect in fragmented data than more complex clusters such as PKS–NRPS hybrids.

3) Clustering into Gene cluster families (GCF): highly fragmented regions often do not contain enough shared sequence/domain architecture to cluster effectively. If the data were complete, fragments from the same underlying locus might be expected to form a coherent GCF (or match to a known cluster), but fragmentation can instead inflate singletons and reduce interpretability.

It is not that this analysis cannot be done on these data, but it is important to distinguish what is biological versus what is driven by fragmentation. For instance Barite_M33_B89 (candidate genus JABSOG01) is a very interesting taxon from a biosynthesis prespective but the statement that it “encoded 107 BGCs… and exhibited the highest number of expressed clusters (n = 75)” isn’t correct from a biological perspective. A more accurate interpretation is that it contains a high number of antiSMASH regions with expression, many of which are likely fragments of fewer underlying loci. The authors have an interesting opportunity to use the long-read transcriptomics data to scaffold contigs and improve assembly/recover fuller BGCs from this strain (clusters are often transcribed as operons). I have not seen this done in this context before and it could be a very novel use of the data generated, where Barite_M33_B89 would be an excellent test case.

My primary concern here is the lack of acknowledgement in the manuscript that fragmented BGCs limit what can be concluded about the biological system. This limitation should be addressed much better and should be taken into account in the interpretation of results.

B) Claims around biosynthesis in archaea

I think contamination or a binning issue is unlikely and the authors have gone to lengths to address this, but while HCN- and aminoglycoside-related genes were detected, further analysis and biosynthetic rationale are required to support these claims (or the claims should be more cautious/suggestive).

Given the presence of other HCN pathways, this is plausible, but I suggest keeping a cautious tone: additional evidence would be needed to validate these bioinformatic indications of HCN production in archaea.

For the reported aminoglycoside/2-deoxystreptamine capacity (Aegir_M39_B193), I’m not sure the current evidence supports this, or this claim requires further analysis. In the antiSMASH region, the “2dos” hit is driven by a gene annotated as aroB / 3-dehydroquinate synthase sitting in a clear shikimate (aromatic amino acid biosynthesis) locus (neighbouring aroA/aroK/aroE/aroD/aroB), which strongly points to primary metabolism rather than an aminoglycoside BGC. The other rule requirement appears to be satisfied by a nearby aminotransferase (here annotated as arnB), which is not uncommon and makes a rule-based false positive likely. Given how extraordinary aminoglycoside biosynthesis would be in archaea, the manuscript should substantially tone down this claim or provide additional analysis with a clearer biochemical/biosynthetic rationale for this BGC - if this can be done convincingly, this finding is exceptional.

Other points

Discussion
Please ensure there is sufficient discussion about the limitations of the data

Methods
Line 390: how much sample was used for each extraction? What was the weight used? Was it the same across all samples? There is very limited description of this part of the data generation.

Reviewer #1

"However, the manuscript shows a severe lack of understanding of the ecology of deep-sea hydrothermal vent microbiomes. There is a lot of interesting work done even using clone libraries and amplicon sequencing of the patterns of microbial diversity from global deep-sea hydrothermal systems."

Addressed well with additional references

"Further, although authors report numerous MAGs from the Sulfolobales, they clearly do not have a good understanding of the revised taxonomy of this group, as these are not restricted to acidic environments as classified now by GTDB. And since the authors use this for classification, they may want to review this taxonomy."

Addressed and GTDB version updated

"The authors also state (line 265) that there is intense competition. Not sure where this has been shown scientifically, but of course, any biofilm there is competition."

Addressed

"The authors state that (line 56) biofilms remain largely unexplored. The bacterial mats the hydrothermal deposits etc. are all biofilms. Some of the samples the authors studied 'look' like hydrothermal deposits"

Addressed

"If these are a 'unique' and remarkable' reservoir of unique bioactive compounds, the authors do not provide compelling evidence to this point. They do not compare they data analytically to other studies of MAGs from deep-sea vents. They do state others have shown such novel secondary metabolites, many with antimicrobial activities' (line 55) so how does this study compare? Do the authors see any of the lysozyme antimicrobials reported by Metcalf et al. from a deep-sea vent organism?"

I am in agreement with the authours lysozymes are not relevant here. However as laid out above I believe both the high proportion of novel BGCs and the diversity of BGC products detected are both very difficult to assess with so a high proportion of fragmented BGCs

Minor comments

All minor issues resolved – no issues

Reviewer #2

"Data Availability and Reproducibility
The two central claims of the manuscript—(i) the discovery of a MAG with exceptionally high biosynthetic potential and (ii) the presence of an HCN pathway in Archaea—are highly significant and challenge long-standing assumptions. However, the absence of accessible raw data prevents validation. The authors mention deposition of raw data under a BioProject, but it appears to be private. No assemblies, MAGs, or BGC sequences are deposited, and the authors only offer to share data upon request. Public availability is essential for reproducibility."

The authors have added bioproject, SRA and I can access the figshare URL.

"MAG with 107 BGCs (Lines 171–175): Reporting 107 BGCs in a 6.7 Mbp MAG is extraordinary. The highest number of BGCs in antiSMASH-DB is 55, in a 12 Mbp genome. This raises concerns about contamination or binning artifacts, possibly from other Pseudomonas spp., which dominate the dataset. Although CheckM reports low contamination, its reliance on single-copy marker genes means it may miss contamination in BGC-rich regions. Alternatively, fragmented BGCs might have been counted multiple times, inflating the total."

This is from Barite_M33_B89 and I agree this is an extraordinary claim which I think would require more robust evidence than is currently available. I agree with the authors assessment this is unlikely to be a contamination issue. The authors acknowledge that all regions detected are on contig edges, so this could inflate the number but this fact needs to be made explicity clear in the manuscript to avoid misunderstanding.

The authors refer to three papers to support unusually high BGCs:
• Liu et al. (2022) – this is not comparable here as they use DeepBGC not antiSMASH – antiSMASH-db uses all RefSeq so is exactly comparable, depending on which version of antiSMASH was used
• Zhang et al. (2024) – This MAG is highly fragmented resulting in the same issue
• Eripogu et al. (2025) – This is again due to a fragmented assembly, complete assemblies show this species has around 52 (NZ_CP046173.1)

"To directly address the reviewer's concern that fragmented BGCs may have been counted multiple times, we performed an additional analysis by clustering the 107 predicted BGCs using BiG-SCAPE with the most stringent similarity cutoff (0.1). If multiple fragments originated from the same underlying BGC, they would cluster into the same GCF. Instead, all BGCs formed singleton GCFs, indicating that antiSMASH did not redundantly split a small number of pathways into many fragments. This supports that the predictions represent distinct BGCs rather than inflation due to algorithmic duplication. Taken together, these analyses suggest that the high BGC count reflects extensive fragmentation in this MAG rather than contamination or artificial inflation."

This unfortunately, is a complete misunderstanding of how BiG-SCAPE works. BiG-SCAPE clusters predicted BGCs based on shared domain content, pairwise similarity metrics and architecture, and it is not designed to infer whether multiple partial predictions originate from the same fragmented pathway. Fragmented BGCs from a single underlying locus are absolutely not expected to cluster, at any cutoff, because each fragment lacks similarity to each other. Therefore, singleton GCFs actually support fragmented driven inflation as this is what you would expect.

To double check I reviewed the regions in this MAG. The antismash visualisation can be viewed here:

https://antismash.secondarymetabolites.org/upload/bacteria-2086baaa-5f42-4faa-a878-3b3157275e17/index.html#r16c1

Very clearly this is an overwhelming fragmented genome with zero incomplete clusters. The issue with this analysis is it increases the number of clusters but also changes the label. For example, many more clusters will be 'NRPS-like' simply because the gene is fragmented and missing the C domain. This doesn't tell us anything about the 'presence' of NRPS-like clusters in the hydrothermal vent metagenome, it just tells us our sequencing data is no deep enough and the resulting assembly is insufficient to answer the question or at least can't be extrapolated beyond what we see.

More accurate terminology would be along the lines of: antiSMASH identified 107 regions across this MAG that had core biosynthetic domains, due to fragmented nature of these regions we're unable to tell how many BGCs are present in this strain, but this suggests this taxa warrants further investigation from a biosynthetic stand point.

"HCN Pathway in Archaea (Lines 237–245): The authors claim the first report of an archaeal HCN pathway, previously known from Pseudomonas. This could also result from contamination, as the MAG's taxonomic assignment is based on the overall bin rather than the contig harboring the pathway. Figure 4's antiSMASH comparison does show a related cluster in another archaeon, which supports the possibility but warrants further verification. It remains unclear whether the reported two-gene operon is incomplete (missing hcnC). Similar caution applies to the reported aminoglycoside pathway (Lines 256–262)."

The addition of MAGpurify2 is a welcome step to address contamination concerns. Please refer to my review regarding my other concerns

"Interpretation of MAG Abundance (Lines 100, 118–136) The authors equate the number of MAGs assigned to a taxon with its abundance in the community. This is not necessarily correct; the number of recovered MAGs depends on assembly and binning success rather than true abundance."

Addressed

"Methodological Concerns
Genome quality thresholds (Lines 377–382): The authors apply non-standard definitions diverging from MIMAG; what they term "near-complete" corresponds to MIMAG HQ, and rRNA/tRNA presence is not considered."

Addressed – MIMAG standards now adhered to

"Dereplication (Lines 383–388): An ANI threshold of 98 % is unusually stringent. While acceptable, comparisons to other studies must note this difference."

Addressed

"antiSMASH Version (Line 409): Inconsistent reporting (v6 vs v7)."

Addressed

"BigMAP Analysis (Lines 427–430): Bowtie is not suitable for long-read data due to its error profiles and read length limitations; it may bias RPKM/TPM normalization. A long-read-aware alignment and normalization to housekeeping genes would yield more reliable abundance estimates. Differences in RNA depletion across samples further complicate comparisons."

Addressed fully by addition of minimap to BiG-MAP analysis and updated text

Additional comments:

"Abstract (Line 20): "Antimicrobial potential" is mentioned but never discussed or supported in the manuscript."

The authors refer to lines 309–315 but need to be careful with their terminology. They state that "RiPP BGCs in our dataset encode a diverse array of antimicrobial peptides, many of which have previously demonstrated in vitro antimicrobial activity." This is not supported unless specific BGCs can be linked to known antimicrobial thiopeptides or sactipeptides with the required biosynthetic genes. Thiopeptides and sactipeptides are biosynthetic classes, and many members are not antimicrobial (e.g. https://doi.org/10.1038/s41467-020-16145-4). While it is reasonable to suggest that some RiPPs expressed here may be antimicrobial, the current wording overstates the evidence; claims of prior in vitro antimicrobial activity require identification of specific, characterized compounds or closely related BGCs – it is likely antimicrobial RiPPs are in the dataset but they need to be named specifically if so.

"Results (Lines 221–224): Reporting raw BGC counts without clustering into GCFs (and distinguishing from MIBiG entries) limits interpretability."

Usually BiG-SCAPE is run with the —mibig flag. This incorperates mibig gene clusters only if there is similarity to clusters in the dataset and I think is easier to understand. Again my concern is there is no mention of the fact that highly fragmented

BGCs will not cluster together in BiG-SCAPE analysis

"Author reply: We thank the reviewer for this comment and appreciate the opportunity to clarify this point. After revisiting the cited study, we note that the authors reported 70,011 BGCs from marine cultured genomes, MAGs, and SAGs, with BGC lengths ranging from 2.0 to 240.7 kb. In our dataset, the shortest BGCs are also approximately ~2 kb, which is consistent with the length range reported in the cited study. Additionally, the average number of BGCs per MAG in the cited study is 1.87 (and 2.06 when including cultured genomes and SAGs), while our dataset contains an average of 2.60 BGCs per MAG. Therefore, our reported values fall within a comparable range. We sincerely appreciate the reviewer's attention to this detail, which has helped us improve the clarity of our discussion."

I think again highly fragmented data will increase the number of gene clusters found by antismash. Inclusion of some assembly statistics E.g. average contig size, distribution of contig sizes from the hydrothermal data would give the readers a bit more context in terms of the sequencing data

(Remarks on code availability)

Version 2:

Reviewer comments:

Reviewer #3

(Remarks to the Author)
I have no further comments about the manuscript.

(Remarks on code availability)
I have no further comments about the manuscript.

Reviewer #4

(Remarks to the Author)
All previous comments have been addressed. I have one minor comment on the updated version of the manuscript: The statement "Only 11.97% (n = 355) of BGCs were complete" (lines 159–160) could be misinterpreted as antiSMASH does not define absolute BGC boundaries. It would be more accurate to state that these BGCs are not located at contig edges and are therefore less likely to be truncated, rather than definitively complete.

(Remarks on code availability)

Reviewer #5

(Remarks to the Author)
Thank you for addressing feedback. The manuscript is far more robust now.

(Remarks on code availability)

Open Access This Peer Review File is licensed under a Creative Commons Attribution 4.0 International License, which permits use, sharing, adaptation, distribution and reproduction in any medium or format, as long as you give appropriate credit to the original author(s) and the source, provide a link to the Creative Commons license, and indicate if changes were made.
In cases where reviewers are anonymous, credit should be given to 'Anonymous Referee' and the source.
The images or other third party material in this Peer Review File are included in the article's Creative Commons license, unless indicated otherwise in a credit line to the material. If material is not included in the article's Creative Commons license and your intended use is not permitted by statutory regulation or exceeds the permitted use, you will need to obtain permission directly from the copyright holder.
To view a copy of this license, visit https://creativecommons.org/licenses/by/4.0/

Author's reply to reviewer comments for manuscript: *Remarkable biosynthetic capacity of Arctic hydrothermal biofilms* by Nguyen et al.

__Authors:__ Thank you for a comprehensive and constructive review of our manuscript and the opportunity to submit a revised version. We have now performed a thorough revision and hopefully met all concerns raised by the reviewers. As stated, the most crucial is the availability of MAG sequence information and raw data. All MAG sequences (with individual BioSamples) and raw metagenomic sequencing data from all samples (SRA), including the metatranscriptomic data (SRA) is now submitted to NCBI (see more details in author reply section below). These data have been set to be released 1st of March 2026, or upon publication of the manuscript. If needed, we can provide copies of the submission spreadsheets.

Reviewer #1 (Remarks to the Author):

This manuscript uses MAGs and long read nanopore RNA sequencing from 9 samples collected from 3 vent fields in the Arctic, to explore the diversity of biosynthetic gene clusters that encode for secondary metabolites. They show that the ribosomally synthesized and post translationally modified peptides are the more prevalent and also the most prevalently expressed across the samples they studied. They also show that some archaea have hydrogen cyanide synthesis pathways. The strength in this paper is that they authors explore these BGCs specifically. And they show they are transcribed.

> *However, the manuscript shows a severe lack of understanding of the ecology of deep-sea hydrothermal vent microbiomes. There is a lot of interesting work done even using clone libraries and amplicon sequencing of the patterns of microbial diversity from global deep-sea hydrothermal systems.*

__Author reply__**:** We acknowledge the scarce description of the work done on taxonomic and functional diversity in deep-sea hydrothermal vent and sediment microbiomes. Line 48-52 have been rewritten and references added to accommodate this and to emphasize the discoveries based on single-cell and genome-resolved metagenomics:
"Deep-sea hydrothermal vent ecosystems represent one of the most extreme and dynamic habitats on Earth, where primary production is predominantly fueled by chemosynthesis rather than photosynthesis (Jannasch, H. W. & Taylor 1984). Since the first discovery in the late 1970s (Corliss et al.1979), a vast microbial diversity has been discovered on a global scale (Crépeau et al., 2011, Ding et al., 2017, Dick G., 2019). The use of single-cell and genome-resolved metagenomics has been a driver for microbial discoveries in hydrothermal and subseafloor environments (Lloyd et al., 2013, Stokke *et al.*, 2015, Li *et al.*, 2016, Meier *et al.*, 2016, Anderson *et al.*, 2017, Dombrowski *et al.*, 2017, Dombrowski *et al.*, 2018, Fortunato *et al.*, 2018, Galambos *et al.*, 2019, McGonigle *et al.*, 2020, Zhou et al., 2022, Speth et al., 2022). Coupled with improved analytical and computational tools this has led to the discovery of novel metabolisms, novel microbial lineages as well as well-known metabolisms in new lineages (Anantharaman *et al.*, 2016, Seitz *et al.*, 2016, Zaremba-Niedzwiedzka *et al.*, 2017, Carr *et al.*, 2019) ."

Further, although authors report numerous MAGs from the Sulfolobales, they clearly do not have a good understanding of the revised taxonomy of this group, as these are ***not restricted to acidic environments as classified now by GTDB****. And since the authors use this for classification, they may want to review this taxonomy.*

Author reply: We appreciate the concern regarding the Sulfolobales taxonomy and environmental restrictions. Naturally, we acknowledge that these Archaea are not restricted to acidic environments, however the reference was pointing to similar extreme environments as investigated in the current manuscript. The following changes made:

Line 132 -133: changed "…. known for thriving in high-temperature, acid environments" to "…. known for thriving in high-temperature, and acid and alkaline environments", and added the reference of Qi et al. 2024 (*Nat Commun*. 2024 May 14;15:4066. doi: 10.1038/s41467-024-48498-5).
In addition, we have adapted the latest GTDB taxonomy implementing GTDB-tk v.2.5.2 and GTDB version R226. Changes made in methods section line 388 to "(GTDB-Tk, v.2.5.2 – GTDB R226 taxonomy).

The authors also state (line 265) that there is intense competition. Not sure where this has been shown scientifically, but of course, any biofilm there is competition.

Author reply: We acknowledge the loosely described feature of the competitive nature of biofilms in general and have modified the text accordingly and added relevant literature. Line 264-265 rewritten as follows: "Microbial biofilms from extreme environments, shaped by extreme environmental pressures and adapted through cooperative and competitive interactions Pandey et al. 2025, Bhat and Roach 2025 and reference therein), represent promising reservoirs of novel bioactive compounds."

The authors state that (line 56) biofilms remain largely unexplored. The bacterial mats the hydrothermal deposits etc. are all biofilms. Some of the samples the authors studied 'look' like hydrothermal deposits.

Author reply: The statement that deep-sea hydrothermal biofilms are largely unexplored, refers to the analysis of BGCs, thus written "unexplored in this context" pointing to the sentence above. All of the biofilms in the study were collected using a hydraulic biosyringe and although perhaps not completely visible on the images, these were biofilms growing on the outside of the venting structures.

If these are a 'unique' and remarkable' reservoir of unique bioactive compounds, the authors do not provide compelling evidence to this point. They do not compare they data analytically to other studies of MAGs from deep-sea vents. They do state others have shown such novel secondary metabolites, many with antimicrobial activities' (line 55) so how does this study compare? Do the authors see any of the lysozyme antimicrobials reported by Metcalf et al. from a deep-sea vent organism?

Author reply: We thank the reviewer for this constructive comment. Our study is designed as an experimental and computational investigation focused specifically on BGCs in

hydrothermal vent biofilms. The central objective of the manuscript is to identify and characterize BGCs using established genome-mining workflows, primarily antiSMASH and BiG-SCAPE, and to assess novelty by comparison to the MIBiG reference database.
Because our methods are BGC-focused, they are optimized to detect secondary metabolite biosynthetic pathways (e.g., NRPS, RiPPs, terpenes). In contrast, lysozymes such as those described by Metcalf et al. are enzymatic antimicrobial proteins rather than BGC-encoded secondary metabolites. These compounds are therefore not detectable using antiSMASH and would require a fundamentally different analytical framework. For this reason, the lysozyme-type antimicrobials are beyond the scope of our current study.
Regarding comparison with other deep-sea vent MAG studies, our conclusions about unique or remarkable potential derive from:

- The high proportion of novel BGCs with no close matches in MIBiG;
- The taxonomic composition of our dataset, which includes archaeal lineages underrepresented in database
- The diversity of BGC predicted products detected.

However, we agree that cross-study comparisons represent an important future direction. To address the reviewer's suggestion, we have added text in the Discussion acknowledging this opinion.

Minor issues.

Some references are cited twice. E.g. # 7 and 14 are the same references

Author reply: We thank the reviewer for pointing this out. We have carefully checked the entire reference list and removed all duplicated entries, including the duplicate noted (#7 and #14). We have also updated the reference list to ensure consistency and added additional relevant references associated with our revisions.

There is no consistency in the figures with regard to sites. It would be great if the sites are always represented in the same order

Author reply: We agree that the order of samples should be consistent in the figures throughout the manuscript. We have revised figures and Table 1, ordering the samples based on Metagenomic ID.

Line 423. It appears this section is in the wrong place?

Author reply: We appreciate the reviewer for pointing this out. The two sections: Biosynthetic gene clusters: screening and expression profiling (from lines 423–430) and Abundance estimation of BGCs from metagenomes and metatranscriptomes (Lines 434–443) contained similar methodological descriptions. To improve readability and reduce repetition, we merged the abundance-estimation details into the preceding section, presenting the workflow in a more cohesive manner. This restructuring clarifies the progression of our analysis while maintaining all relevant technical information.

With MAGs, it's better to refer to genes as being undetected, rather than absent. Ie.

They just were not sequenced, so not detected. Maybe they are absent, but with incomplete genomes its misleading to consider genes absent.

Author reply: We completely agree with the reviewer comment as absent in any -omics study in general is not equivalent with not present in the sample. Line 326-329 stating "mostly in M39 and absent in M37" has been modified as a result of the new transcriptomic mapping using minimap2 to: "Their known roles in microbial signaling and defense, coupled with transcriptomic evidence from 162 BGCs from all screened metatranscriptomes, highlight their functional relevance."

Line 379. If authors are using the Bowers et al ref as they state, then their nearly complete genomes are actually the high quality ' MAGs'.

Author reply: Please see reply to reviewer 2 comments. Non-standard definitions have been corrected to MIMAG standard definitions.

Be consistent with acronyms. Not sure AVF was ever defined.

Author reply: We thank the reviewer for noticing this oversight and apologize for the inconsistency in the acronym. We have replaced AVF with the full term Aegir's Vent Field (line 366) throughout the manuscript to ensure clarity and consistency.

Reviewer #2 (Remarks to the Author):

The manuscript "Remarkable biosynthetic capacity of Arctic hydrothermal biofilms" reports a metagenomic and metatranscriptomic analysis of microbial biofilms from Arctic hydrothermal vents. The authors identify over 1,000 bacterial and archaeal MAGs encoding nearly 3,000 biosynthetic gene clusters (BGCs), highlighting RiPPs as dominant and suggesting novel findings such as a MAG with 107 BGCs and the first evidence of a hydrogen cyanide biosynthetic pathway in Archaea. The paper presents exciting and potentially groundbreaking discoveries about the biosynthetic diversity of hydrothermal vent microbiomes. However, the lack of publicly available data and insufficient methodological transparency make it impossible to verify the major claims. Several significant concerns arise regarding data reproducibility, potential MAG contamination, and the robustness of key findings**.**

Data Availability and Reproducibility

The two central claims of the manuscript—(i) the discovery of a MAG with exceptionally high biosynthetic potential and (ii) the presence of an HCN pathway in Archaea—are highly significant and challenge long-standing assumptions. However, the absence of accessible raw data prevents validation. The authors mention deposition of raw data under a BioProject, but it appears to be private. No assemblies, MAGs, or BGC

sequences are deposited, and the authors only offer to share data upon request. Public availability is essential for reproducibility.

Author reply: We appreciate the concern on data availability and reproducibility and have made an effort to have all data connected to the study publically available. For reasons out of our control, BioSample data has been delayed, however we have now been able to upload all sequence data of MAGs. Raw read files, both for the metagenomes (illumina) and the cDNA (Nanopore) are uploaded to the SRA. The Data Availability section has been updated accordingly. In addition, a FigShare project for the manuscript is created which includes all identified BGCs in the study as GenBank files, network file from BiG-SCAPE, AA sequences of hcnA/B and hcnC from the Heimdallarchaeion Aegir_M39_B184 MAG including a superimposed structural image of the hcnC AlphaFold3 models from Aegir_M39_B184 and *Pseudomonas fluorescence*. The open access figshare DOI added to the manuscript will be made publicly open upon publication of the manuscript. To access for review, please use: https://figshare.com/s/2b40ca10c8dbe70b8c5e

MAG with 107 BGCs (Lines 171–175): Reporting 107 BGCs in a 6.7 Mbp MAG is extraordinary. The highest number of BGCs in antiSMASH-DB is 55, in a 12 Mbp genome. This raises concerns about contamination or binning artifacts, possibly from other Pseudomonas spp., which dominate the dataset. Although CheckM reports low contamination, its reliance on single-copy marker genes means it may miss contamination in BGC-rich regions. Alternatively, fragmented BGCs might have been counted multiple times, inflating the total.

Author reply:

We agree with the reviewer that 107 predicted BGCs in a 6.7 Mbp MAG is unusually high compared with typical values reported in isolate genomes. However, all 107 BGCs in this MAG are fragmented: the average BGC length is 7.82 kb and the average gene count is 2.98. These values indicate that the predictions likely represent numerous short fragmented BGCs, which makes a high count plausible given the MAG size.

We applied the same quality-controlled workflow to all MAGs in the study: only MAGs that passed our completeness and contamination thresholds were retained, annotated with Prokka, and screened with antiSMASH using identical parameters. While contamination is always a concern in metagenomic binning, we took care to minimize it throughout the assembly and refinement process and maintained consistency across all samples. Even though CheckM cannot detect contamination in non-marker regions, the absence of duplicated marker genes and the coherent taxonomy of the bin support that gross contamination is low. The possibility of unusually BGC-rich genomes should also be considered. As noted in Liu et al. (2022) (https://doi.org/10.1016/j.jmb.2022.167597), bacterial reference genomes can contain from 0 up to 243 BGCs, demonstrating that extremely BGC-dense genomes do exist, even though they are not common in antiSMASH-DB. Furthermore, a study of agricultural soil microbiomes identified a MAG belonging to Acidobacteriota that harbors 65 BGCs (Zhang et al., 2024, mSystems, https://doi.org/10.1128/msystems.01263-23). These findings show that high BGC counts are not unprecedented. Eripogu *et al.* (2025) (DOI: 10.1128/mbio.00947-25)

identified 104 biosynthetic gene clusters in the *Nocardia* strain NBRC 100888 (genome size: 8.63 Mbp). Although such high BGC counts are rare, this example illustrates that bacterial genomes can, in some cases, contain over 100 BGCs.
To directly address the reviewer's concern that fragmented BGCs may have been counted multiple times, we performed an additional analysis by clustering the 107 predicted BGCs using BiG-SCAPE with the most stringent similarity cutoff (0.1). If multiple fragments originated from the same underlying BGC, they would cluster into the same GCF. Instead, all BGCs formed singleton GCFs, indicating that antiSMASH did not redundantly split a small number of pathways into many fragments. This supports that the predictions represent distinct BGCs rather than inflation due to algorithmic duplication.
Taken together, these analyses suggest that the high BGC count reflects extensive fragmentation in this MAG rather than contamination or artificial inflation.

HCN Pathway in Archaea (Lines 237–245): The authors claim the first report of an archaeal HCN pathway, previously known from Pseudomonas. This could also result from contamination, as the MAG's taxonomic assignment is based on the overall bin rather than the contig harboring the pathway. Figure 4's antiSMASH comparison does show a related cluster in another archaeon, which supports the possibility but warrants further verification. It remains unclear whether the reported two-gene operon is incomplete (missing hcnC). Similar caution applies to the reported aminoglycoside pathway (Lines 256–262).

Author reply: To assure that the observed pathways were preserved on contigs of key MAGs, additional verification was conducted using MAGpurify2. Both for the HCN (Aegir_M39_B184) and the aminoglycoside pathway (Aegir_M39_B193), the genes were retained on contigs with low contaminant probability score (0.05) classified to the same taxa as the overall MAGs, class *Heimdallarchaeia* within the phylum *Asgardarchaeota* and class *Thermoproteia* within the phylum *Thermoproteota,* respectively. Hence, combined with the other quality assessments of the MAGs, these contigs are likely to be part of the overall described MAGs. A description of the MAG purification is added to the methods section and the section titled Previously unrecognized biosynthetic capacities in Archaea have been revised accordingly (line 236-262). In this section a clarification has been made to also show the presence of hcnC in Aegir_M39_B184, i.e a two gene, rather than three genes (as in Pseudomonas), for a complete hydrogen-cyanide synthase. A superimposed figure of the AlphaFold3 models of hcnC from Aegir_M39_B184 and Pseudomonas is added to the FigShare project and linked in the manuscript.

Interpretation of MAG Abundance (Lines 100, 118–136)
The authors equate the number of MAGs assigned to a taxon with its abundance in the community. This is not necessarily correct; the number of recovered MAGs depends on assembly and binning success rather than true abundance.

Author reply: We appreciate that this was pointed out and we agree completely. To clarify, we have modified this paragraph to highlight that the relative abundance was achieved by mapping to de-replicated MAGs using the coupled paired-read files from each biofilm sample. Various factors as mentioned, such as assembly and binning, would affect this estimate, although we are looking at the mapped fraction and not true abundance, acknowledging the un-mapped fraction of the dataset. Hence, the abundance of a particular taxon derived from this estimate.

Line 118 modified: "... mapped reads to de-replicated MAGs using CoverM,"
Line 383-386 modified to: "Relative abundance of de-replicated MAGs in each biofilm was estimated by mapping filtered paired-en reads from each biofilm sample to MAGs using CoverM v0.6.1(https://github.com/wwood/CoverM)."

Methodological Concerns

Genome quality thresholds (Lines 377–382): The authors apply non-standard definitions diverging from MIMAG; what they term "near-complete" corresponds to MIMAG HQ, and rRNA/tRNA presence is not considered.

Author reply: We acknowledge the use of non-standard quality definitions and have corrected according to MIMAG standards, also including rRNA and tRNA presence.

Lines 377-382 as follow: "The quality of each MAG was estimated with CheckM1 (v1.0.12) and CheckM2 (1.1.0) in addition to the presence/absence of rRNA and tRNA determined by barrnap v0.9 (+REF) and tRNAscan v2.0.12 (+REF), respectively. Low-quality genomes following the MIMAG standard [63] (completeness <50% and contamination > 10%) were removed from downstream analysis, with the exception of 25 MAGs first estimated to be medium-quality by checkM1. High-quality MAGs are defined with completeness ≥90% and contamination ≤5%, and includes at least one copy of 16S, 23S and 5S rRNA genes and ≥ 18 unique tRNAs. Medium-quality MAGs are defined with completeness ≥90% and contamination ≤10%."

Dereplication (Lines 383–388): An ANI threshold of 98 % is unusually stringent. While acceptable, comparisons to other studies must note this difference.

Author reply: A threshold of 98% is not necessarily unusually stringent, depending on scope and larger initiatives such as the Earth Hologenome Initiative, have been using this value arguing for an optimal threshold to keep genome diversity while minimizing cross-mapping issues. The value is also stated as valid in the dRep github pages. Since dRep uses a default minimum coverage threshold of 10%, a 98% ANI cutoff would be valid to retain genome diversity in the current study, balancing redundancy removal and mis-mapping issues.

Line 384-385 modified to: "Although stringent, an ANI value of 98% was selected to balance genome diversity, while minimizing cross-mapping issues and avoiding redundancy > 98% (Delmont et al. 2018).

antiSMASH Version (Line 409): Inconsistent reporting (v6 vs v7).

Author reply: We thank the reviewer for pointing out this inconsistency and apologize for the confusion caused by mentioning both versions of antiSMASH. The reference to antiSMASH version 6.0 in line 159 was intended only to clarify that the bacteriocin class was reclassified as RiPP-like since that release, not to indicate that this version was used for any data analysis. We have revised the sentence to read: "RiPP-like clusters, reclassified from bacteriocins in antiSMASH since the release of version 6.0 …"
We confirm that antiSMASH v7.0 was the only version used for all analyses presented in this manuscript.

BigMAP Analysis (Lines 427–430): Bowtie is not suitable for long-read data due to its error profiles and read length limitations; it may bias RPKM/TPM normalization. A long-read-aware alignment and normalization to housekeeping genes would yield more reliable abundance estimates.
Differences in RNA depletion across samples further complicate comparisons.

Author reply: We thank the reviewer for this insightful and constructive comment. We fully agree that Bowtie2 is not optimal for long-read data due to its sensitivity to error profiles and read length limitations. As Bowtie2 is the default aligner in the BiG-MAP pipeline, we initially used it to evaluate the performance of the standard workflow on our dataset. Following the reviewer's valuable suggestion, we have now re-mapped all metatranscriptomic reads using Minimap2 (v2.26, r1175) with the -x map-ont preset to ensure more accurate alignment of Nanopore reads.
To implement this improvement, we removed the default setting of Bowtie2 in the BiG-MAP.map code, replaced it with Minimap2 and adjusted the TPM normalisation accordingly. This re-analysis resulted in quantitative differences in gene expression estimates but did not alter the overall expression trends or main conclusions of the study.
The revised results, including updated figures and tables, have been incorporated throughout the manuscript. The specific changes resulting from this updated mapping approach are summarised below.

Results section (lines 165–166, 169–171, 174, 176, 182, 185, 198-200, 203-205): Updated expression values and corresponding descriptions to reflect the re-analysis using Minimap2.
Results section (line 183): Added the sentence *"Similarly, the Other class showed significantly higher expression compared to NRP (p = 0.0127) and Polyketide (p = 0.0179)."* to reflect the updated analysis.
Results section (lines 183–186): Deleted this section because the results changed following re-analysis with Minimap2.
Results section (line 192): Added six additional phyla to match the updated results from the revised analysis.

Discussion (lines 299, 301–304, 307, 311–312, 315, 320–323, 327–329): Updated taxonomic information to reflect the new classification and revised transcriptional level descriptions based on the updated mapping results.
Methods section (lines 426–443): Updated the description of the mapping method to reflect the modification of the BiG-MAP pipeline from the default Bowtie2 aligner to Minimap2 and revised the corresponding references accordingly.

Additional Comments

Abstract (Line 20): "Antimicrobial potential" is mentioned but never discussed or supported in the manuscript.

Author reply: We thank the reviewer for pointing this out. We have revised the Discussion to explicitly address the antimicrobial potential of AMOR BGCs. In the revised text, we added a section (lines 309-315) describing the antimicrobial peptides detected and actively expressed in the AMOR biofilms, including well-characterized groups such as thiopeptides and sactipeptides. These examples strengthen and clarify our discussion of the antimicrobial potential encoded by AMOR BGCs.

Introduction (Lines 44–47): Missing citation.

Author reply: We thank the reviewer for pointing this out. As suggested, we have added the relevant references to this section to strengthen the introduction and support the statements made.

Results (Lines 202–215): Not all reported metabolites are necessarily antimicrobial; the wording should be refined.

Author reply: We have revised the wording in this section to clarify that not all detected BGCs necessarily produce antimicrobial metabolites, as their functions can vary depending on environmental conditions, regulatory dynamics, and expression levels. Our intention is to highlight that hydrothermal biofilm BGCs represent a promising source for future exploration of antimicrobial compound diversity, rather than implying that all BGCs identified are antimicrobial in nature.

Results (Lines 221–224): Reporting raw BGC counts without clustering into GCFs (and distinguishing from MIBiG entries) limits interpretability.

Author reply: We appreciate the reviewer's comment. We were initially unsure which aspect of Lines 221–224 was considered unclear, as this section reports results based on GCF-level comparisons. Specifically, we examined the Gene Cluster Families (GCFs) generated from all BGCs originating from both the biofilm metagenomes and the MIBiG 3.1 database. The numbers reported in this section intentionally refer to GCFs rather than raw BGC counts. To

improve clarity and ensure the results are fully interpretable, we have added Supplementary Table 6, which lists all GCFs generated by BiG-SCAPE along with the corresponding BGCs assigned to each GCF. This table provides a direct link between individual BGCs and their GCF identifiers. We have also included the BiG-SCAPE cluster file, which contains the network structure of all GCFs. We have added Supplementary Figure 7, which presents the complete cluster analysis including all gene cluster families from both the biofilm BGCs and the MIBiG reference dataset. This figure clarifies and supports the statements made in this section. We believe these additions fully address the reviewer's concern and enhance transparency in the presentation of our clustering results.

Discussion (Lines 281–282): The cited study reports similar numbers but under different size thresholds (>10 kbp); comparisons should be made carefully.

Author reply: We thank the reviewer for this comment and appreciate the opportunity to clarify this point. After revisiting the cited study, we note that the authors reported 70,011 BGCs from marine cultured genomes, MAGs, and SAGs, with BGC lengths ranging from 2.0 to 240.7 kb. In our dataset, the shortest BGCs are also approximately ~2 kb, which is consistent with the length range reported in the cited study. Additionally, the average number of BGCs per MAG in the cited study is 1.87 (and 2.06 when including cultured genomes and SAGs), while our dataset contains an average of 2.60 BGCs per MAG. Therefore, our reported values fall within a comparable range. We sincerely appreciate the reviewer's attention to this detail, which has helped us improve the clarity of our discussion.

Other revisions:
Line 103-105, 108-109, 131, 154, 162, 315, 319-324, 388: Updated to reflect the new GTDB version and the corresponding revised taxonomic assignments.
Lines 139–140, 144, and 148: Updated the statistical results of the Bray–Curtis and Sørensen analyses following the revision of GTDB taxonomic assignments. Although the numerical values changed slightly, the overall trends remained consistent with the previous version. **Supplementary Figures 1 and 2** have been updated accordingly, as both are based on these analyses.
Line 153: Updated the number of BGCs encoded by the phylum Bacteroidota, as the new GTDB release split this group into Bacteroidota and Bacteroidota_A.
Line 219: removed the word 'novel'
Line 408: Updated the protocol link to a publicly accessible version, as the protocol has now been published online for broader accessibility.

REVIEWER COMMENTS

We would like to thank the reviewers for the detailed review and highly constructive feedback on our work further improving the quality of the manuscript. We sincerely appreciate the time and effort. We hope we have met all the concerns that were raised.

Reviewer #3 (Remarks to the Author):

A few minor comments on the revised manuscript.
- Line 20-23: Is it non-ribosomal peptide synthetases that show antimicrobial activity? The way it is written it implies it is the synthetases are tested for microbial activity, change to reflect that they are observed to be expressed.
Author reply: re-phrased to **"**RiPPs and non-ribosomal peptide synthetases (NRPs), known to encode metabolites with antimicrobial potential, are represented among the most expressed transcripts."

- line 79: no reference or spelling out MIBiG at the first occurrence.
Author reply: MIBiG reference added and section revised: "Comparative analysis with the Minimum Information about a Biosynthetic Gene Cluster database (MIBiG) ….."

- Line 103: '>2000 bp' needs to be put in context 'minimum contig size in MAGs is >200bp' or similar.
Author reply: size specified to: "(minimum contig size > 2000 bp)"

-Line 120: Add in that between 20 and 75.3% of the community was presented by the ~1140 MAGs in this study to give the readers a sense of what % MAGs the BGCs represent.
Author reply: We agree that this is a valid number to show in this context and have added the following: "Overall, between 20 and 75.3% of the sequenced microbial community was represented by the MAGs in the study". These values have also been added as summarised values in Supplementary Table 3.

- Line 147: is the Dice-Sorensen Coefficient? I didn't think there was a Sorensen p-value. also, not obvious in statistical tests in method section.
Author reply: Thank you for the clarification. We agree that the Dice–Sørensen coefficient is a descriptive similarity measure and does not produce a p-value. In our analysis, the reported p-values were obtained from an explicit permutation test (1000 permutations). For each pair of samples, we computed Sørensen similarity on presence/absence data, then generated a null distribution by randomly shuffling the presence/absence vector of one sample while keeping the other fixed, and recalculating the coefficient. We have revised the Methods and Results sections to describe this permutation-based significance assessment and to avoid implying that the Sørensen coefficient itself yields a p-value.

- Line 189: 'adaptation' to what?
Author reply: Suggested adaptations are already described in the subsequent section line 190 - 203

- Line 244: was or were?
Author reply: changed to “were”.

- Line 262: Prometheoarchaeum syntrophicum should be unitalicized, Candidatus is italicised.
Author reply: yes, we agree, Prometheoarchaeum syntrophicum is now unitalicized.

- Lines 397-402: Need to include a 'Contig Number' column for each MAG, this gives the reader a sense of the quality more so than N50.
Author reply: contig numbers added to Supplementary Table 3

- Lines 406-407: Do the >90%, <5% medium quality genomes have 5,16,23S rRNA genes,>18 tRNAs like the high-quality genomes? it needs to be at least stated as to their criteria, if not consistent between the two categories.
Author reply: Our study has followed the established MIMAG standard suggested by Bowers et al. (referenced) whereby medium-quality MAGs are not required to contain this information, however we have already included presence/absence of 5S, 16S, 23S and tRNA information in the Supplementary Table 3 for all MAGs in the study.

Reviewer #3 (Remarks on code availability):

I assume that the initial reviewers did this.

Reviewer #4 (Remarks on code availability):

The code is available and includes both a README and a requirements file for installation and use.

Reviewer #1
If these are a ‘unique’ and remarkable’ reservoir of unique bioactive compounds, the authors do not provide compelling evidence to this point. They do not compare they data analytically to other studies of MAGs from deep-sea vents. They do state others have shown such novel secondary metabolites, many with antimicrobial activities’ (line 55) so how does this study compare? Do the authors see any of the lysozyme antimicrobials reported by Metcalf et al. from a deep-sea vent organism?

Author reply: We thank the reviewer for this constructive comment. Our study is designed as an experimental and computational investigation focused specifically on BGCs in hydrothermal vent biofilms. The central objective of the manuscript is to identify and characterize BGCs using established genome-mining workflows, primarily antiSMASH and BiG-SCAPE, and to assess novelty by comparison to the MIBiG reference database. Because our methods are BGC-focused, they are optimized to detect secondary metabolite biosynthetic pathways (e.g., NRPS, RiPPs, terpenes). In contrast, lysozymes such as those described by Metcalf et al. are enzymatic antimicrobial proteins rather than BGC-encoded

secondary metabolites. These compounds are therefore not detectable using antiSMASH and would require a fundamentally different analytical framework. For this reason, the lysozyme-type antimicrobials are beyond the scope of our current study.
Regarding comparison with other deep-sea vent MAG studies, our conclusions about unique or remarkable potential derive from:

● The high proportion of novel BGCs with no close matches in MIBiG;
● The taxonomic composition of our dataset, which includes archaeal lineages underrepresented in database
● The diversity of BGC predicted products detected.

However, we agree that cross-study comparisons represent an important future direction. To address the reviewer's suggestion, we have added text in the Discussion acknowledging this opinion.

I agree that antiSMASH is not designed to identify lysozymes directly. However, I have some reservations regarding the interpretation of novelty based solely on the number of BGCs detected per MAG. In this context, I refer the authors to the discussion below regarding the application of antiSMASH to fragmented MAGs.

In addition, particularly when studying understudied taxa, it is good to keep in mind that antiSMASH may not detect all BGCs. Genomes with relatively few identified BGCs may still be of interest, for example they may have non-canonical or highly divergent clusters that are not recognized by current prediction tools. Relying exclusively on BGC counts as a measure of novelty may therefore overlook aspects of the broader chemical potential of these organisms.

Author reply: We agree that the application of antiSMASH to fragmented MAGs may lead to underestimation of BGC numbers, splitting of BGCs across contigs, or failure to detect highly divergent clusters. To reduce these potential biases, we restricted our analysis to medium- and high-quality MAGs and interpreted BGC counts cautiously. We have now clarified this limitation in the revised Discussion.

We also agree that, particularly for understudied and phylogenetically distinct taxa, antiSMASH may not capture the full biosynthetic repertoire. Non-canonical or highly divergent biosynthetic pathways may remain undetected by current rule-based prediction tools. In the revised manuscript, we have adjusted our wording to avoid equating BGC number directly with novelty. Instead, we frame BGC abundance as an initial indicator of biosynthetic potential within the constraints of current computational tools. To promote future reanalysis as prediction algorithms improve, all assemblies, annotations, and antiSMASH outputs have been made publicly available.

We emphasize that this study represents a first systematic exploration of biosynthetic potential in these Arctic hydrothermal vent communities. Ongoing work integrating additional omics approaches (e.g., metaproteomics) will further refine our understanding of their functional and chemical diversity.

Some references are cited twice. E.g. # 7 and 14 are the same references
Author reply: We thank the reviewer for pointing this out. We have carefully checked the entire reference list and removed all duplicated entries, including the duplicate noted (#7 and #14). We have also updated the reference list to ensure consistency and added additional relevant references associated with our revisions**.**
The reference to BiG-MAP is missing from the reference list.
Author reply: The reference to BiG-MAP is now added

Reviewer #2
Data Availability and Reproducibility
The two central claims of the manuscript—(i) the discovery of a MAG with exceptionally high biosynthetic potential and (ii) the presence of an HCN pathway in Archaea—are highly significant and challenge long-standing assumptions. However, the absence of accessible raw data prevents validation. The authors mention deposition of raw data under a BioProject, but it appears to be private. No assemblies, MAGs, or BGC sequences are deposited, and the authors only offer to share data upon request. Public availability is essential for reproducibility.
Author reply: We appreciate the concern on data availability and reproducibility and have made an effort to have all data connected to the study publically available. For reasons out of our control, BioSample data has been delayed, however we have now been able to upload all sequence data of MAGs. Raw read files, both for the metagenomes (illumina) and the cDNA (Nanopore) are uploaded to the SRA. The Data Availability section has been updated accordingly. In addition, a FigShare project for the manuscript is created which includes all identified BGCs in the study as GenBank files, network file from BiG-SCAPE, AA sequences of hcnA/B and hcnC from the Heimdallarchaeion Aegir_M39_B184 MAG including a superimposed structural image of the hcnC AlphaFold3 models from Aegir_M39_B184 and *Pseudomonas fluorescence*. The open access figshare DOI added to the manuscript will be made publicly open upon publication of the manuscript. To access for review, please use: https://figshare.com/s/2b40ca10c8dbe70b8c5e
I appreciate that the BGC .gbk files (amongst others) are now available. However, the MAGs themselves are not currently accessible via the provided BioSample IDs. As the authors note, this may be beyond their control and related to issues on the NCBI side. It does limit the ability to independently validate several of the claims. This is particularly relevant for results such as the prediction of more than 100 BGCs from a single MAG, which would benefit from validation starting from the underlying assemblies.
Author reply: see reply to reviewer 5.

MAG with 107 BGCs (Lines 171–175): Reporting 107 BGCs in a 6.7 Mbp MAG is extraordinary. The highest number of BGCs in antiSMASH-DB is 55, in a 12 Mbp genome. This raises concerns about contamination or binning artifacts, possibly from other

Pseudomonas spp., which dominate the dataset. Although CheckM reports low contamination, its reliance on single-copy marker genes means it may miss contamination in BGC-rich regions. Alternatively, fragmented BGCs might have been counted multiple times, inflating the total.

Author reply:

We agree with the reviewer that 107 predicted BGCs in a 6.7 Mbp MAG is unusually high compared with typical values reported in isolate genomes. However, all 107 BGCs in this MAG are fragmented: the average BGC length is 7.82 kb and the average gene count is 2.98. These values indicate that the predictions likely represent numerous short fragmented BGCs, which makes a high count plausible given the MAG size.

We applied the same quality-controlled workflow to all MAGs in the study: only MAGs that passed our completeness and contamination thresholds were retained, annotated with Prokka, and screened with antiSMASH using identical parameters. While contamination is always a concern in metagenomic binning, we took care to minimize it throughout the assembly and refinement process and maintained consistency across all samples. Even though CheckM cannot detect contamination in non-marker regions, the absence of duplicated marker genes and the coherent taxonomy of the bin support that gross contamination is low.

The possibility of unusually BGC-rich genomes should also be considered. As noted in Liu et al. (2022) (https://doi.org/10.1016/j.jmb.2022.167597), bacterial reference genomes can contain from 0 up to 243 BGCs, demonstrating that extremely BGC-dense genomes do exist, even though they are not common in antiSMASH-DB. Furthermore, a study of agricultural soil microbiomes identified a MAG belonging to Acidobacteriota that harbors 65 BGCs (Zhang et al., 2024, mSystems, https://doi.org/10.1128/msystems.01263-23). These findings show that high BGC counts are not unprecedented. Eripogu *et al.* (2025) (DOI: 10.1128/mbio.00947-25) identified 104 biosynthetic gene clusters in the *Nocardia* strain NBRC 100888 (genome size: 8.63 Mbp). Although such high BGC counts are rare, this example illustrates that bacterial genomes can, in some cases, contain over 100 BGCs.

To directly address the reviewer's concern that fragmented BGCs may have been counted multiple times, we performed an additional analysis by clustering the 107 predicted BGCs using BiG-SCAPE with the most stringent similarity cutoff (0.1). If multiple fragments originated from the same underlying BGC, they would cluster into the same GCF. Instead, all BGCs formed singleton GCFs, indicating that antiSMASH did not redundantly split a small number of pathways into many fragments. This supports that the predictions represent distinct BGCs rather than inflation due to algorithmic duplication.

Taken together, these analyses suggest that the high BGC count reflects extensive fragmentation in this MAG rather than contamination or artificial inflation.

When looking at a subset of the predicted "BGCs" from Barite_M33_B89 and found that seem to originate from a highly fragmented MAG. Several of the clusters I manually inspected consist of only a single, potentially fragmented, gene, which may further complicate downstream analyses, as clustering algorithms such as BiG-SCAPE often rely on genomic context and shared sequence features for effective grouping. For example, if the individual genes of a single gene cluster are distributed across multiple fragmented "BGCs" these fragments will not cluster together using BiG-SCAPE, regardless of stringency, as they lack sufficient shared sequence similarity. As a result, such fragments are likely to be classified as singletons (as mentioned), despite originating from the same underlying BGC. When, on top of that, genes are truncated and may contain only partial domains, coverage scores drop substantially, making successful clustering even less likely.
Without going into the full details of the other studies mentioned, one appears to rely on a fundamentally different, DL-based approach rather than antiSMASH's rule-based framework, which may reasonably explain the higher number of predicted clusters reported there. The remaining studies are also based on contig-level assemblies or MAGs, where similar fragmentation-related issues are likely to occur. In contrast, antiSMASH-db is constructed from complete, chromosome, and scaffold level assembled genomes. In this context, the current max of ~55 predicted BGCs in the db may represent a more realistic upper bound of detection.
While it remains possible that this MAG genuinely harbors an unusually high number of BGCs exceeding the 55 mentioned, I consider it likely that the reported count reflects artificial inflation of the scores due to assembly fragmentation. I therefore recommend revisiting the BGC predictions with explicit consideration of fragmentation and reporting separate counts for higher-confidence, more complete BGCs vs all predicted clusters. This would substantially strengthen the interpretation of the results.
Author reply: We agree that fragmented BGCs may inflate BGC counts and affect clustering approaches such as BiG-SCAPE. In particular, truncated genes and incomplete domain architectures may reduce similarity scores and lead to an increased number of singletons. To address this concern, we have now added a BGC completeness column to Supplementary Table 4. We have revised the Discussion to explicitly acknowledge the impact of MAG fragmentation on BGC prediction and clustering. We now clarify that some predicted BGCs, especially those located at contig edges, may represent partial clusters rather than complete operons, and that the reported BGC counts should therefore be interpreted with caution. Importantly, our conclusions are primarily based on overall diversity patterns and transcriptomic support, which remain robust despite these limitations. Future work integrating improved assemblies and completeness-aware filtering will further refine these estimates.

HCN Pathway in Archaea (Lines 237–245): The authors claim the first report of an archaeal HCN pathway, previously known from Pseudomonas. This could also result from contamination, as the MAG's taxonomic assignment is based on the overall bin rather than the contig harboring the pathway. Figure 4's antiSMASH comparison does show a related cluster in another archaeon, which supports the possibility but warrants further verification. It remains unclear whether the reported two-gene operon is incomplete (missing hcnC). Similar caution applies to the reported aminoglycoside pathway (Lines 256–262).
Author reply: To assure that the observed pathways were preserved on contigs of key MAGs, additional verification was conducted using MAGpurify2. Both for the HCN

(Aegir_M39_B184) and the aminoglycoside pathway (Aegir_M39_B193), the genes were retained on contigs with low contaminant probability score (0.05) classified to the same taxa as the overall MAGs, class *Heimdallarchaeia* within the phylum *Asgardarchaeota* and class *Thermoproteia* within the phylum *Thermoproteota,* respectively. Hence, combined with the other quality assessments of the MAGs, these contigs are likely to be part of the overall described MAGs. A description of the

MAG purification is added to the methods section and the section titled Previously unrecognized biosynthetic capacities in Archaea have been revised accordingly (line 236-262). In this section a clarification has been made to also show the presence of hcnC in Aegir_M39_B184, i.e a two gene, rather than three genes (as in Pseudomonas), for a complete hydrogen-cyanide synthase. A superimposed figure of the AlphaFold3 models of hcnC from Aegir_M39_B184 and Pseudomonas is added to the FigShare project and linked in the manuscript.

For Aegir_M39_B184, I am a bit uncertain about the robustness of the prediction, as the cluster is located at a contig edge and may therefore be incomplete. In the revised manuscript, the authors note that "In addition to high-scoring blastp hits to other Archaea (data not shown), this further supports the potential for archaeal cyanogenesis.". Showing a comparison of this gene cluster with homologous regions from other archaeal genomes would be very helpful and would further strengthen this conclusion. Could these data be included, either in the main text or as supplementary material?

Author reply: We have revised the section starting with line 260 to clarify and acknowledge the fact that the identified HCN genes are located at a contig edge. Additional data to support the presence of a complete archaeal HCN has been added by hits to highly similar sequences in *Candidatus* Heimdallarchaeaceae from the Guayamas Basin (Supplementary Table 8). The sequences from Guayamas Basin was also identified by antiSMASH as HCN (revised Supplementary figure 4) and show high structural homology to the proposed hcnAB from the Aegir_M39_B184 MAG (revised Supplementary figure 5). Importantly, the proposed HCN from some of these Guayamas MAGs are flanked by other genes, suggesting a complete cluster of hcnAB and hcnC (Supplementary figure 4).

For Aegir_M39_B193, I attempted to verify the result, however, antiSMASH v8 does not return any predicted BGCs in this region. Could the authors double-check this prediction, as it raises the possibility that changes between antiSMASH versions may affect BGC detection. Clarification on this point would be helpful, as I'm now considering that the original prediction may have been less robust and thus removed from antiSMASH v8.

Author reply: We appreciate the concern regarding this BGC and have thus repeated our analysis using the antiSMASH v8. However, we still get the same results as with v7, identified the 2dos BGC (2-deoxy-streptamine aminoglycoside) from this MAG. See reply to reviewer #5 below for detailed discussion on this BGC.

Dereplication (Lines 383–388): An ANI threshold of 98 % is unusually stringent. While acceptable, comparisons to other studies must note this difference.

Author reply: A threshold of 98% is not necessarily unusually stringent, depending on scope and larger initiatives such as the Earth Hologenome Initiative, have been using this value arguing for an optimal threshold to keep genome diversity while minimizing cross-mapping issues. The value is also stated as valid in the dRep github pages. Since dRep uses a default minimum coverage threshold of 10%, a 98% ANI cutoff would be valid to retain genome diversity in the current study, balancing redundancy removal and mis-mapping issues.
Line 384-385 modified to: "Although stringent, an ANI value of 98% was selected to balance genome diversity, while minimizing cross-mapping issues and avoiding redundancy > 98% (Delmont et al. 2018).

Small typo: paired-en reads -> paired-end reads

Author reply: Line 421 typo revised: paired-end reads

BigMAP Analysis (Lines 427–430): Bowtie is not suitable for long-read data due to its error profiles and read length limitations; it may bias RPKM/TPM normalization. A long-read-aware alignment and normalization to housekeeping genes would yield more reliable abundance estimates.
Differences in RNA depletion across samples further complicate comparisons.

Author reply: We thank the reviewer for this insightful and constructive comment. We fully agree that Bowtie2 is not optimal for long-read data due to its sensitivity to error profiles and read length limitations. As Bowtie2 is the default aligner in the BiG-MAP pipeline, we initially used it to evaluate the performance of the standard workflow on our dataset.
Following the reviewer's valuable suggestion, we have now re-mapped all metatranscriptomic reads using Minimap2 (v2.26, r1175) with the -x map-ont preset to ensure more accurate alignment of Nanopore reads.
To implement this improvement, we removed the default setting of Bowtie2 in the BiG-MAP.map code, replaced it with Minimap2 and adjusted the TPM normalisation accordingly. This re-analysis resulted in quantitative differences in gene expression estimates but did not alter the overall expression trends or main conclusions of the study.
The revised results, including updated figures and tables, have been incorporated throughout the manuscript. The specific changes resulting from this updated mapping approach are summarised below.
Results section (lines 165–166, 169–171, 174, 176, 182, 185, 198-200, 203-205): Updated expression values and corresponding descriptions to reflect the re-analysis using Minimap2.
Results section (line 183): Added the sentence *"Similarly, the Other class showed significantly higher expression compared to NRP (p = 0.0127) and Polyketide (p = 0.0179)."* to reflect the updated analysis.
Results section (lines 183–186): Deleted this section because the results changed following re-analysis with Minimap2.
Results section (line 192): Added six additional phyla to match the updated results from the revised analysis.
Discussion (lines 299, 301–304, 307, 311–312, 315, 320–323, 327–329): Updated taxonomic information to reflect the new classification and revised transcriptional level descriptions based on the updated mapping results.
Methods section (lines 426–443): Updated the description of the mapping method to reflect the modification of the BiG-MAP pipeline from the default Bowtie2 aligner to Minimap2 and revised the corresponding references accordingly.
Looks good to me. One suggestion would be to open an issue or feature request on the BiG-MAP GitHub page to recommend this update, or, if possible, to submit a pull request with the developed code so that the tool's developers can integrate this improvement into the main branch for future use.
Author reply: Following the reviewer's suggestion, we have opened an issue on the BiG-MAP GitHub repository to report the TPM normalization and to suggest support for long-read mapping using minimap2.

Additional Comments
Abstract (Line 20): "Antimicrobial potential" is mentioned but never discussed or supported in the manuscript.
Author reply: We thank the reviewer for pointing this out. We have revised the Discussion to explicitly address the antimicrobial potential of AMOR BGCs. In the revised text, we added a section (lines 309-315) describing the antimicrobial peptides detected and actively expressed in

the AMOR biofilms, including well-characterized groups such as thiopeptides and sactipeptides. These examples strengthen and clarify our discussion of the antimicrobial potential encoded by AMOR BGCs.
As far as I can tell, the newly added comparison is made to thiopeptide and sactipeptide BGCs reported in the literature in general, rather than to direct, one-to-one sequence similarity between the BGCs identified in this work and previously characterized clusters from literature. As this study does not include bioactivity activity assays, the current wording in the abstract ("show strong antimicrobial potential") may be misleading. I therefore recommend softening or rephrasing this statement to better reflect the scope of the presented evidence.
Author reply: We agree that this may be misleading and have revised line 20-22 in the abstract accordingly: "RiPPs and non-ribosomal peptide synthetases (NRPs), known to encode metabolites with antimicrobial potential, are represented among the most expressed transcripts".

Results (Lines 202–215): Not all reported metabolites are necessarily antimicrobial; the wording should be refined.
Author reply: We have revised the wording in this section to clarify that not all detected BGCs necessarily produce antimicrobial metabolites, as their functions can vary depending on environmental conditions, regulatory dynamics, and expression levels. Our intention is to highlight that hydrothermal biofilm BGCs represent a promising source for future exploration of antimicrobial compound diversity, rather than implying that all BGCs identified are antimicrobial in nature.
The refined wording still appears to imply that all identified BGCs have antimicrobial properties. For example, the statement "Hydrothermal biofilms harbored a diverse array of antimicrobial BGCs, many of which were actively transcribed" suggests a bioactivity/function that is not directly supported for all BGCs. While the authors provide a helpful and nuanced discussion a few lines later, it would be preferable to state explicitly from the outset that BGCs encode a wide range of compounds, of which only a subset may be bioactive, including antimicrobials. The authors could then note that RiPPs are more frequently associated with bioactivity, while still acknowledging that this does not exclude alternative or currently unknown functions. Adding words like "potential" to the claims will already help softening it. In addition, it is important to note that BGCs themselves are not antimicrobial, rather, the compounds potentially derived from these clusters may have antimicrobial activity. Similar wording appears in several places throughout the manuscript and would benefit from refinement to more accurately reflect this distinction.
Author reply: We have now toned down our original wording, which overstated the evidence by implicitly linking RiPP biosynthetic classes to antimicrobial activity without identification of specific compounds.
We have revised the Discussion to avoid generalizing that RiPP BGCs encode antimicrobial peptides. Instead, we now state that these BGCs represent RiPP with antimicrobial potential (lines 318-320), and that while some members of classes such as thiopeptides and sactipeptides are known to exhibit antimicrobial activity, this cannot be inferred without detailed characterization of individual clusters (lines 325-327).

Reviewer #5 (Remarks to the Author):

NCOMMS-25-73582A

Remarkable biosynthetic capacity of Arctic hydrothermal biofilms

The manuscript is an interesting and important metagenomic assessment of biosynthetic capacity across 9 samples from 3 vent fields. Notably, the authors have also performed ONT long-read RNA analysis alongside the eDNA sequencing, which adds a functional component to the study and is one of the manuscript's main strengths, alongside the interesting and unique environment being studied. My role was primarily to check that the previous reviewer's comments were addressed sufficiently. After reading the revised manuscript and Reviewer #2's comments, I still have concerns about limitations of the data and the strength of some claims. In particular, there is insufficient discussion of the limitations of assessing biosynthetic capability when almost 90% of antiSMASH regions are fragmented, which adds significant caveats to the findings presented. This especially affects (i) the reported number of gene clusters (inflated due to fragmentation) and (ii) the dominance of RiPPs (the easiest BGC type to detect on short contigs). Overall, I think the manuscript is well written and a notable contribution on an interesting microbial ecosystem, but this limitation needs to be addressed more clearly.

Major concerns:

A) Fragmented MAGS with short contigs and misinterpretation

There is an acknowledgement that only ~12% of BGCs are complete, but many results are still reported as if fragmentation has minimal impact. The implications are significant for the interpretation of results:

1) Number of BGCs: Antismash reports 'regions' of the genome that hit specific pHMM rules. These 'regions' are not definitely biosynthetic gene clusters. As evidenced by Barite_M33_B89 many of these 'BGCs' are in fact just fragments of NRPS genes that happen to contain an A, PCP and C domain: https://antismash.secondarymetabolites.org/upload/bacteria-2086baaa-5f42-4faa-a878-3b3157275e17/index.html#r4c1. Given the typical size of NRPS BGCs, what would have been a single NRPS cluster spanning perhaps 120–200 kb can appear as 10–15 "BGCs" in fragmented data (e.g. B89 has 47 regions <5 kb). This is an artefact of the fragmented sequencing/assembly, not the biological reality. Reporting that Barite_M33_B89 "has 107 BGCs" is therefore misleading: antiSMASH detected 107 regions/contigs containing core biosynthetic domains.

2) Type of BGCs: fragmentation biases what you are more likely to detect. For example, NRPS-like regions can be over-called because only partial NRPS domain content is recovered before a contig break. Moreover, RiPPs (often among the shortest genes that trigger antiSMASH rules) will be much easier to detect in fragmented data than more complex clusters such as PKS–NRPS hybrids

3) Clustering into Gene cluster families (GCF): highly fragmented regions often do not contain enough shared sequence/domain architecture to cluster effectively. If the data were complete, fragments from the same underlying locus might be expected to form a coherent GCF (or match to a known cluster), but fragmentation can instead inflate singletons and reduce interpretability.

It is not that this analysis cannot be done on these data, but it is important to distinguish what is biological versus what is driven by fragmentation. For instance Barite_M33_B89 (candidate genus JABSOG01) is a very interesting taxon from a biosynthesis prespective but the statement that it "encoded 107 BGCs… and exhibited the highest number of expressed clusters (n = 75)" isn't correct from a biological perspective. A more accurate interpretation is that it contains a high number of antiSMASH regions with expression, many of which are likely fragments of fewer underlying loci. The authors have an interesting opportunity to use the long-read transcriptomics data to scaffold contigs and improve assembly/recover fuller BGCs from this strain (clusters are often transcribed as operons). I have not seen this done in this context before and it could be a very novel use of the data generated, where Barite_M33_B89 would be an excellent test case.

My primary concern here is the lack of acknowledgement in the manuscript that fragmented BGCs limit what can be concluded about the biological system. This limitation should be addressed much better and should be taken into account in the interpretation of results.
Author reply: We thank the reviewer for this important comment. This point was also raised by Reviewer #4 (comment on reply to Reviewer #2) and has been addressed accordingly. Briefly, we have added a BGC completeness metric to Supplementary Table 4 and revised the Discussion to explicitly acknowledge the impact of fragmented BGCs on detection, clustering, and biological interpretation. Regarding long-read transcriptomics to improve assembly and recover more complete clusters, we agree and this has been considered, but at this stage the authors believe this needs to be included in a larger methods paper to assess implementing such data on the full metagenomic dataset.

B) Claims around biosynthesis in archaea

I think contamination or a binning issue is unlikely and the authors have gone to lengths to address this, but while HCN- and aminoglycoside-related genes were detected, further analysis and biosynthetic rationale are required to support these claims (or the claims should be more cautious/suggestive).

Given the presence of other HCN pathways, this is plausible, but I suggest keeping a cautious tone: additional evidence would be needed to validate these bioinformatic indications of HCN production in archaea.
Author reply: See also the additional information added to remarks by reviewer # 2, however we do acknowledge the fact that all -omics related discoveries are in need of biochemical verification, and thus we have modified the final section in the discussion, line 367-370, as follows ; "However, additional biochemical and/or cultivation efforts, integrating comparative genomics, transcriptomics, and metabolomics, are needed to verify the HCN pathway in archaea and to elucidate the functional significance of HCN production and its

impact on biofilm community dynamics.”

For the reported aminoglycoside/2-deoxystreptamine capacity (Aegir_M39_B193), I’m not sure the current evidence supports this, or this claim requires further analysis. In the antiSMASH region, the “2dos” hit is driven by a gene annotated as aroB / 3-dehydroquinate synthase sitting in a clear shikimate (aromatic amino acid biosynthesis) locus (neighbouring aroA/aroK/aroE/aroD/aroB), which strongly points to primary metabolism rather than an aminoglycoside BGC. The other rule requirement appears to be satisfied by a nearby aminotransferase (here annotated as arnB), which is not uncommon and makes a rule-based false positive likely. Given how extraordinary aminoglycoside biosynthesis would be in archaea, the manuscript should substantially tone down this claim or provide additional analysis with a clearer biochemical/biosynthetic rationale for this BGC - if this can be done convincingly, this finding is exceptional.
Author reply: We agree that the current evidence is not sufficient to conclude the 2-deoxystreptamine biosynthetic capacity in this archaeal genome. We have therefore toned down this claim in the revised manuscript.
However, we note that BLASTp analysis of the gene annotated by antiSMASH as *aroB* (3-dehydroquinate synthase) also returns significant hits to members of the DegT/DnrJ/EryC1/StrS aminotransferase family in NCBI, including homologues from other archaeal genomes. These enzymes belong to the sugar aminotransferase (SAT) family, which is classified in Pfam and is typically associated with the biosynthesis of aminoglycosides and other secondary metabolites. A search of the antiSMASH database also identified similar 2-deoxystreptamine BGC annotations in archaeal genomes derived from cultured isolates and complete genomes. For example, in the genome NC_016070.1 (*Thermoproteus tenax* Kra 1), a predicted 2dos BGC includes a DegT/DnrJ/EryC1/StrS family aminotransferase co-localized within the same operon as shikimate pathway genes, including shikimate kinase and shikimate dehydrogenase. This genomic organization suggests that such arrangements may be common across archaeal species.
To further assess this, we performed a comparative analysis between the Aegir_M39_B193 region and the T. tenax locus using clinker/CAGECAT, which revealed conservation of several gene groups across the two regions (clinker result file provided on FigShare: 10.6084/m9.figshare.31948944).
Nevertheless, we acknowledge that these observations remain suggestive rather than conclusive, and we have revised the manuscript accordingly to present this as a tentative finding requiring further biochemical validation.

Other points

Discussion
Please ensure there is sufficient discussion about the limitations of the data
Author reply: We have revised the Discussion to point out the limitations of our data, particularly the impact of MAG and BGC fragmentation on BGC detection, clustering, and biological interpretation. This point has also been addressed in response to similar comments from other reviewers.

Methods
Line 390: how much sample was used for each extraction? What was the weight used? Was it the same across all samples? There is very limited description of this part of the data generation.
Author reply: We thank the reviewer for highlighting the need for greater clarity regarding sample input for nucleic acid extraction.
We have now added a new column to Supplementary Table 1 and 2 specifying the concentration of DNA/RNA from each sample. Due to limited sample availability, variability in biofilm density, and differences in storage time prior to extraction, the input material was not identical across samples.

Reviewer 5 commenting replies to Reviewer #1

"However, the manuscript shows a severe lack of understanding of the ecology of deep-sea hydrothermal vent microbiomes. There is a lot of interesting work done even using clone libraries and amplicon sequencing of the patterns of microbial diversity from global deep-sea hydrothermal systems."

Addressed well with additional references

"Further, although authors report numerous MAGs from the Sulfolobales, they clearly do not have a good understanding of the revised taxonomy of this group, as these are not restricted to acidic environments as classified now by GTDB. And since the authors use this for classification, they may want to review this taxonomy."

Addressed and GTDB version updated

"The authors also state (line 265) that there is intense competition. Not sure where this has been shown scientifically, but of course, any biofilm there is competition."

Addressed

"The authors state that (line 56) biofilms remain largely unexplored. The bacterial mats the hydrothermal deposits etc. are all biofilms. Some of the samples the authors studied 'look' like hydrothermal deposits"

Addressed

"If these are a 'unique' and remarkable' reservoir of unique bioactive compounds, the authors do not provide compelling evidence to this point. They do not compare they data analytically to other studies of MAGs from deep-sea vents. They do state others have shown such novel secondary metabolites, many with antimicrobial activities' (line 55) so how does this study compare? Do the authors see any of the lysozyme antimicrobials reported by Metcalf et al. from a deep-sea vent organism?"

I am in agreement with the authours lysozymes are not relevant here. However as laid out above I believe both the high proportion of novel BGCs and the diversity of BGC products detected are both very difficult to assess with so a high proportion of fragmented BGCs

Author reply: We thank the reviewer for acknowledging that lysozymes are not relevant in this context. We agree that the high proportion of fragmented BGCs can affect the assessment of both the apparent novelty and the diversity of predicted BGC products. As noted above, we have now addressed this limitation in the Discussion and included a BGC completeness metric in the supplementary data. We further clarify that our conclusions are primarily based on overall diversity patterns and should be interpreted with caution in light of potential fragmentation.

Minor comments

All minor issues resolved – no issues

Reviewer 5 commenting replies to Reviewer #2

"Data Availability and Reproducibility
The two central claims of the manuscript—(i) the discovery of a MAG with exceptionally high biosynthetic potential and (ii) the presence of an HCN pathway in Archaea—are highly significant and challenge long-standing assumptions. However, the absence of accessible raw data prevents validation. The authors mention deposition of raw data under a BioProject, but it appears to be private. No assemblies, MAGs, or BGC sequences are deposited, and the authors only offer to share data upon request. Public availability is essential for reproducibility."

The authors have added bioproject, SRA and I can access the figshare URL.

"MAG with 107 BGCs (Lines 171–175): Reporting 107 BGCs in a 6.7 Mbp MAG is extraordinary. The highest number of BGCs in antiSMASH-DB is 55, in a 12 Mbp genome. This raises concerns about contamination or binning artifacts, possibly from other Pseudomonas spp., which dominate the dataset. Although CheckM reports low contamination, its reliance on single-copy marker genes means it may miss contamination in BGC-rich regions. Alternatively, fragmented BGCs might have been counted multiple times, inflating the total."

This is from Barite_M33_B89 and I agree this is an extraordinary claim which I think would require more robust evidence than is currently available. I agree with the authors assessment this is unlikely to be a contamination issue. The authors acknowledge that all regions detected are on contig edges, so this could inflate the number but this fact needs to be made explicity clear in the manuscript to avoid misunderstanding.

The authors refer to three papers to support unusually high BGCs:

- Liu et al. (2022) – this is not comparable here as they use DeepBGC not antiSMASH – antiSMASH-db uses all RefSeq so is exactly comparable, depending on which version of antiSMASH was used
- Zhang et al. (2024) – This MAG is highly fragmented resulting in the same issue

• Eripogu et al. (2025) – This is again due to a fragmented assembly, complete assemblies show this species has around 52 (NZ_CP046173.1)

"To directly address the reviewer's concern that fragmented BGCs may have been counted multiple times, we performed an additional analysis by clustering the 107 predicted BGCs using BiG-SCAPE with the most stringent similarity cutoff (0.1). If multiple fragments originated from the same underlying BGC, they would cluster into the same GCF. Instead, all BGCs formed singleton GCFs, indicating that antiSMASH did not redundantly split a small number of pathways into many fragments. This supports that the predictions represent distinct BGCs rather than inflation due to algorithmic duplication. Taken together, these analyses suggest that the high BGC count reflects extensive fragmentation in this MAG rather than contamination or artificial inflation."

This unfortunately, is a complete misunderstanding of how BiG-SCAPE works. BiG-SCAPE clusters predicted BGCs based on shared domain content, pairwise similarity metrics and architecture, and it is not designed to infer whether multiple partial predictions originate from the same fragmented pathway. Fragmented BGCs from a single underlying locus are absolutely not expected to cluster, at any cutoff, because each fragment lacks similarity to each other. Therefore, singleton GCFs actually support fragmented driven inflation as this is what you would expect.

To double check I reviewed the regions in this MAG. The antismash visualisation can be viewed here: https://antismash.secondarymetabolites.org/upload/bacteria-2086baaa-5f42-4faa-a878-3b3157275e17/index.html#r16c1

Very clearly this is an overwhelming fragmented genome with zero incomplete clusters. The issue with this analysis is it increases the number of clusters but also changes the label. For example, many more clusters will be 'NRPS-like' simply because the gene is fragmented and missing the C domain. This doesn't tell us anything about the 'presence' of NRPS-like clusters in the hydrothermal vent metagenome, it just tells us our sequencing data is no deep enough and the resulting assembly is insufficient to answer the question or at least can't be extrapolated beyond what we see.

More accurate terminology would be along the lines of: antiSMASH identified 107 regions across this MAG that had core biosynthetic domains, due to fragmented nature of these regions we're unable to tell how many BGCs are present in this strain, but this suggests this taxa warrants further investigation from a biosynthetic stand point.
Author reply: We acknowledge that the release of all sequence information has taken time, however, all data related to the current study should now be publicly available at NCBI. We have also added a column with the GenBank accession numbers of each MAG in Supplementary Table 3. We thank the reviewer and agree that the original wording overstated the interpretation. We have now revised the manuscript to adopt more accurate and cautious terminology.
In the Results (Lines 179–183), we now state that antiSMASH identified 107 BGC-like regions containing core biosynthetic domains in this MAG, rather than inferring complete BGCs, and we have softened the interpretation accordingly.

In the Discussion (Lines 293–295), we have also revised the text to ensure that the biosynthetic potential of these biofilms is presented more cautiously, avoiding overinterpretation.
These changes ensure that our conclusions reflect the limitations of the data while still highlighting the relevance of this MAG for future biosynthetic investigation.

"HCN Pathway in Archaea (Lines 237–245): The authors claim the first report of an archaeal HCN pathway, previously known from Pseudomonas. This could also result from contamination, as the MAG's taxonomic assignment is based on the overall bin rather than the contig harboring the pathway. Figure 4's antiSMASH comparison does show a related cluster in another archaeon, which supports the possibility but warrants further verification. It remains unclear whether the reported two-gene operon is incomplete (missing hcnC). Similar caution applies to the reported aminoglycoside pathway (Lines 256–262)."

The addition of MAGpurify2 is a welcome step to address contamination concerns. Please refer to my review regarding my other concerns

"Interpretation of MAG Abundance (Lines 100, 118–136) The authors equate the number of MAGs assigned to a taxon with its abundance in the community. This is not necessarily correct; the number of recovered MAGs depends on assembly and binning success rather than true abundance."

Addressed

"Methodological Concerns
Genome quality thresholds (Lines 377–382): The authors apply non-standard definitions diverging from MIMAG; what they term "near-complete" corresponds to MIMAG HQ, and rRNA/tRNA presence is not considered."

Addressed – MIMAG standards now adhered to

"Dereplication (Lines 383–388): An ANI threshold of 98 % is unusually stringent. While acceptable, comparisons to other studies must note this difference."

Addressed

"antiSMASH Version (Line 409): Inconsistent reporting (v6 vs v7)."

Addressed

"BigMAP Analysis (Lines 427–430): Bowtie is not suitable for long-read data due to its error profiles and read length limitations; it may bias RPKM/TPM normalization. A long-read-aware alignment and normalization to housekeeping genes would yield more reliable abundance estimates. Differences in RNA depletion across samples further complicate comparisons."

Addressed fully by addition of minimap to BiG-MAP analysis and updated text

Additional comments:

"Abstract (Line 20): “Antimicrobial potential” is mentioned but never discussed or supported in the manuscript."

The authors refer to lines 309–315 but need to be careful with their terminology. They state that “RiPP BGCs in our dataset encode a diverse array of antimicrobial peptides, many of which have previously demonstrated in vitro antimicrobial activity.” This is not supported unless specific BGCs can be linked to known antimicrobial thiopeptides or sactipeptides with the required biosynthetic genes. Thiopeptides and sactipeptides are biosynthetic classes, and many members are not antimicrobial (e.g. https://doi.org/10.1038/s41467-020-16145-4). While it is reasonable to suggest that some RiPPs expressed here may be antimicrobial, the current wording overstates the evidence; claims of prior in vitro antimicrobial activity require identification of specific, characterized compounds or closely related BGCs – it is likely antimicrobial RiPPs are in the dataset but they need to be named specifically if so.
Author reply: We have now toned down our original wording, which overstated the evidence by implicitly linking RiPP biosynthetic classes to antimicrobial activity without identification of specific compounds.

We have revised the Discussion to avoid generalizing that RiPP BGCs encode antimicrobial peptides. Instead, we now state that these BGCs represent RiPP with antimicrobial potential (lines 318-320), and that while some members of classes such as thiopeptides and sactipeptides are known to exhibit antimicrobial activity, this cannot be inferred without detailed characterization of individual clusters (lines 325-327).

"Results (Lines 221–224): Reporting raw BGC counts without clustering into GCFs (and distinguishing from MIBiG entries) limits interpretability."

Usually BiG-SCAPE is run with the -–mibig flag. This incorperates mibig gene clusters only if there is similarity to clusters in the dataset and I think is easier to understand. Again my concern is there is no mention of the fact that highly fragmented BGCs will not cluster together in BiG-SCAPE analysis

"Author reply: We thank the reviewer for this comment and appreciate the opportunity to clarify this point. After revisiting the cited study, we note that the authors reported 70,011 BGCs from marine cultured genomes, MAGs, and SAGs, with BGC lengths ranging from 2.0 to 240.7 kb. In our dataset, the shortest BGCs are also approximately ~2 kb, which is consistent with the length range reported in the cited study. Additionally, the average number of BGCs per MAG in the cited study is 1.87 (and 2.06 when including cultured genomes and SAGs), while our dataset contains an average of 2.60 BGCs per MAG. Therefore, our reported values fall within a comparable range. We sincerely appreciate the reviewer’s attention to this detail, which has helped us improve the clarity of our discussion."

I think again highly fragmented data will increase the number of gene clusters found by antismash. Inclusion of some assembly statistics E.g. average contig size, distribution of

contig sizes from the hydrothermal data would give the readers a bit more context in terms of the sequencing data

Author reply: We agree that inclusion of i.e number of contigs in each MAGs will help to understand the fragmentation and is now also included in the discussion. Also see comments to previous reviewer comments above. The contig numbers have been added to a revised version of the Supplementary Table 3. The fragmentation of MAGs in such studies are not surprising, however we agree that this should also be stated to give more context.

As suggested in the Author checklist, we have adjusted the title from «Remarkable biosynthetic capacity of Arctic hydrothermal biofilms» to «**Diverse Biosynthetic Pathways in Arctic Hydrothermal Biofilms**»

REVIEWERS' COMMENTS

Reviewer #3 (Remarks to the Author):

I have no further comments about the manuscript.

Reviewer #3 (Remarks on code availability):

I have no further comments about the manuscript.

Reviewer #4 (Remarks to the Author):

All previous comments have been addressed. I have one minor comment on the updated version of the manuscript: The statement "Only 11.97% (n = 355) of BGCs were complete" (lines 159–160) could be misinterpreted as antiSMASH does not define absolute BGC boundaries. It would be more accurate to state that these BGCs are not located at contig edges and are therefore less likely to be truncated, rather than definitively complete.

Author reply: We agree and have adjusted this sentence to take this into account: "Only 11.97% (n = 355) of BGCs were complete (i.e not located at contig edges, and thus less likely to be truncated), while the majority (n = 2,610) were fragmented».

Reviewer #5 (Remarks to the Author):

Thank you for addressing feedback. The manuscript is far more robust now.

This is a cross-review of a manuscript focusing on metagenomic and metatranscriptomic data derived from three vent fields in an Arctic setting. There are several points for which I would like to provide follow-up questions or remarks (in blue).

Reviewer #1

> *If these are a 'unique' and remarkable' reservoir of unique bioactive compounds, the authors do not provide compelling evidence to this point. They do not compare they data analytically to other studies of MAGs from deep-sea vents. They do state others have shown such novel secondary metabolites, many with antimicrobial activities' (line 55) so how does this study compare? Do the authors see any of the lysozyme antimicrobials reported by Metcalf et al. from a deep-sea vent organism?*

Author reply: We thank the reviewer for this constructive comment. Our study is designed as an experimental and computational investigation focused specifically on BGCs in hydrothermal vent biofilms. The central objective of the manuscript is to identify and characterize BGCs using established genome-mining workflows, primarily antiSMASH and BiG-SCAPE, and to assess novelty by comparison to the MIBiG reference database.
Because our methods are BGC-focused, they are optimized to detect secondary metabolite biosynthetic pathways (e.g., NRPS, RiPPs, terpenes). In contrast, lysozymes such as those described by Metcalf et al. are enzymatic antimicrobial proteins rather than BGC-encoded secondary metabolites. These compounds are therefore not detectable using antiSMASH and would require a fundamentally different analytical framework. For this reason, the lysozyme-type antimicrobials are beyond the scope of our current study.
Regarding comparison with other deep-sea vent MAG studies, our conclusions about unique or remarkable potential derive from:

- The high proportion of novel BGCs with no close matches in MIBiG;
- The taxonomic composition of our dataset, which includes archaeal lineages underrepresented in database
- The diversity of BGC predicted products detected.

However, we agree that cross-study comparisons represent an important future direction. To address the reviewer's suggestion, we have added text in the Discussion acknowledging this opinion.

I agree that antiSMASH is not designed to identify lysozymes directly. However, I have some reservations regarding the interpretation of novelty based solely on the number of BGCs detected per MAG. In this context, I refer the authors to the discussion below regarding the application of antiSMASH to fragmented MAGs.

In addition, particularly when studying understudied taxa, it is good to keep in mind that antiSMASH may not detect all BGCs. Genomes with relatively few identified BGCs may still be of interest, for example they may have non-canonical or highly divergent clusters that are not recognized by current prediction tools. Relying exclusively on BGC counts as a measure of novelty may therefore overlook aspects of the broader chemical potential of these organisms.

Some references are cited twice. E.g. # 7 and 14 are the same references

Author reply: We thank the reviewer for pointing this out. We have carefully checked the entire reference list and removed all duplicated entries, including the duplicate noted (#7 and #14). We have also updated the reference list to ensure consistency and added additional relevant references associated with our revisions**.**

The reference to BiG-MAP is missing from the reference list.

Reviewer #2

Data Availability and Reproducibility

The two central claims of the manuscript—(i) the discovery of a MAG with exceptionally high biosynthetic potential and (ii) the presence of an HCN pathway in Archaea—are highly significant and challenge long-standing assumptions. However, the absence of accessible raw data prevents validation. The authors mention deposition of raw data under a BioProject, but it appears to be private. No assemblies, MAGs, or BGC sequences are deposited, and the authors only offer to share data upon request. Public availability is essential for reproducibility.

Author reply: We appreciate the concern on data availability and reproducibility and have made an effort to have all data connected to the study publically available. For reasons out of our control, BioSample data has been delayed, however we have now been able to upload all sequence data of MAGs. Raw read files, both for the metagenomes (illumina) and the cDNA (Nanopore) are uploaded to the SRA. The Data Availability section has been updated accordingly. In addition, a FigShare project for the manuscript is created which includes all identified BGCs in the study as GenBank files, network file from BiG-SCAPE, AA sequences of hcnA/B and hcnC from the Heimdallarchaeion Aegir_M39_B184 MAG including a superimposed structural image of the hcnC AlphaFold3 models from Aegir_M39_B184 and *Pseudomonas fluorescence*. The open access figshare DOI added to the manuscript will be made publicly open upon publication of the manuscript. To access for review, please use: https://figshare.com/s/2b40ca10c8dbe70b8c5e

I appreciate that the BGC .gbk files (amongst others) are now available. However, the MAGs themselves are not currently accessible via the provided BioSample IDs. As the authors note, this may be beyond their control and related to issues on the NCBI side. It does limit the ability to independently validate several of the claims. This is particularly relevant for results such as the prediction of more than 100 BGCs from a single MAG, which would benefit from validation starting from the underlying assemblies.

MAG with 107 BGCs (Lines 171–175): Reporting 107 BGCs in a 6.7 Mbp MAG is extraordinary. The highest number of BGCs in antiSMASH-DB is 55, in a 12 Mbp genome. This raises concerns about contamination or binning artifacts, possibly from other

Pseudomonas spp., which dominate the dataset. Although CheckM reports low contamination, its reliance on single-copy marker genes means it may miss contamination in BGC-rich regions. Alternatively, fragmented BGCs might have been counted multiple times, inflating the total.

Author reply:

We agree with the reviewer that 107 predicted BGCs in a 6.7 Mbp MAG is unusually high compared with typical values reported in isolate genomes. However, all 107 BGCs in this MAG are fragmented: the average BGC length is 7.82 kb and the average gene count is 2.98. These values indicate that the predictions likely represent numerous short fragmented BGCs, which makes a high count plausible given the MAG size.

We applied the same quality-controlled workflow to all MAGs in the study: only MAGs that passed our completeness and contamination thresholds were retained, annotated with Prokka, and screened with antiSMASH using identical parameters. While contamination is always a concern in metagenomic binning, we took care to minimize it throughout the assembly and refinement process and maintained consistency across all samples. Even though CheckM cannot detect contamination in non-marker regions, the absence of duplicated marker genes and the coherent taxonomy of the bin support that gross contamination is low.

The possibility of unusually BGC-rich genomes should also be considered. As noted in Liu et al. (2022) (https://doi.org/10.1016/j.jmb.2022.167597), bacterial reference genomes can contain from 0 up to 243 BGCs, demonstrating that extremely BGC-dense genomes do exist, even though they are not common in antiSMASH-DB. Furthermore, a study of agricultural soil microbiomes identified a MAG belonging to Acidobacteriota that harbors 65 BGCs (Zhang et al., 2024, mSystems, https://doi.org/10.1128/msystems.01263-23). These findings show that high BGC counts are not unprecedented. Eripogu *et al.* (2025) (DOI: 10.1128/mbio.00947-25) identified 104 biosynthetic gene clusters in the *Nocardia* strain NBRC 100888 (genome size: 8.63 Mbp). Although such high BGC counts are rare, this example illustrates that bacterial genomes can, in some cases, contain over 100 BGCs.

To directly address the reviewer's concern that fragmented BGCs may have been counted multiple times, we performed an additional analysis by clustering the 107 predicted BGCs using BiG-SCAPE with the most stringent similarity cutoff (0.1). If multiple fragments originated from the same underlying BGC, they would cluster into the same GCF. Instead, all BGCs formed singleton GCFs, indicating that antiSMASH did not redundantly split a small number of pathways into many fragments. This supports that the predictions represent distinct BGCs rather than inflation due to algorithmic duplication.

Taken together, these analyses suggest that the high BGC count reflects extensive fragmentation in this MAG rather than contamination or artificial inflation.

When looking at a subset of the predicted "BGCs" from Barite_M33_B89 and found that seem to originate from a highly fragmented MAG. Several of the clusters I manually inspected consist of only a single, potentially fragmented, gene, which may further complicate downstream analyses, as clustering algorithms such as BiG-SCAPE often rely on genomic context and shared sequence features for effective grouping. For example, if the individual genes of a single gene cluster are distributed across multiple fragmented "BGCs" these fragments will not cluster together using BiG-SCAPE, regardless of stringency, as they lack sufficient shared sequence similarity. As a result, such fragments are likely to be classified as singletons (as mentioned), despite originating from the same underlying BGC. When, on top of that, genes are truncated and may contain only partial domains, coverage scores drop substantially, making successful clustering even less likely.

Without going into the full details of the other studies mentioned, one appears to rely on a fundamentally different, DL-based approach rather than antiSMASH's rule-based framework, which may reasonably explain the higher number of predicted clusters reported there. The remaining studies are also based on contig-level assemblies or MAGs, where similar fragmentation-related issues are likely to occur. In contrast, antiSMASH-db is constructed from complete, chromosome, and scaffold level assembled genomes. In this context, the current max of ~55 predicted BGCs in the db may represent a more realistic upper bound of detection.

While it remains possible that this MAG genuinely harbors an unusually high number of BGCs exceeding the 55 mentioned, I consider it likely that the reported count reflects artificial inflation of the scores due to assembly fragmentation. I therefore recommend revisiting the BGC predictions with explicit consideration of fragmentation and reporting separate counts for higher-confidence, more complete BGCs vs all predicted clusters. This would substantially strengthen the interpretation of the results.

> *HCN Pathway in Archaea (Lines 237–245): The authors claim the first report of an archaeal HCN pathway, previously known from Pseudomonas. This could also result from contamination, as the MAG's taxonomic assignment is based on the overall bin rather than the contig harboring the pathway. Figure 4's antiSMASH comparison does show a related cluster in another archaeon, which supports the possibility but warrants further verification. It remains unclear whether the reported two-gene operon is incomplete (missing hcnC). Similar caution applies to the reported aminoglycoside pathway (Lines 256–262).*

Author reply: To assure that the observed pathways were preserved on contigs of key MAGs, additional verification was conducted using MAGpurify2. Both for the HCN (Aegir_M39_B184) and the aminoglycoside pathway (Aegir_M39_B193), the genes were retained on contigs with low contaminant probability score (0.05) classified to the same taxa as the overall MAGs, class *Heimdallarchaeia* within the phylum *Asgardarchaeota* and class *Thermoproteia* within the phylum *Thermoproteota,* respectively. Hence, combined with the other quality assessments of the MAGs, these contigs are likely to be part of the overall described MAGs. A description of the

MAG purification is added to the methods section and the section titled Previously unrecognized biosynthetic capacities in Archaea have been revised accordingly (line 236-262). In this section a clarification has been made to also show the presence of hcnC in Aegir_M39_B184, i.e a two gene, rather than three genes (as in Pseudomonas), for a complete hydrogen-cyanide synthase. A superimposed figure of the AlphaFold3 models of hcnC from Aegir_M39_B184 and Pseudomonas is added to the FigShare project and linked in the manuscript.

For Aegir_M39_B184, I am a bit uncertain about the robustness of the prediction, as the cluster is located at a contig edge and may therefore be incomplete. In the revised manuscript, the authors note that "In addition to high-scoring blastp hits to other Archaea (data not shown), this further supports the potential for archaeal cyanogenesis.". Showing a comparison of this gene cluster with homologous regions from other archaeal genomes would be very helpful and would further strengthen this conclusion. Could these data be included, either in the main text or as supplementary material?

For Aegir_M39_B193, I attempted to verify the result, however, antiSMASH v8 does not return any predicted BGCs in this region. Could the authors double-check this prediction, as it raises the possibility that changes between antiSMASH versions may affect BGC detection. Clarification on this point would be helpful, as I'm now considering that the original prediction may have been less robust and thus removed from antiSMASH v8.

> *Dereplication (Lines 383–388): An ANI threshold of 98 % is unusually stringent. While acceptable, comparisons to other studies must note this difference.*

Author reply: A threshold of 98% is not necessarily unusually stringent, depending on scope and larger initiatives such as the Earth Hologenome Initiative, have been using this value arguing for an optimal threshold to keep genome diversity while minimizing cross-mapping issues. The value is also stated as valid in the dRep github pages. Since dRep uses a default minimum coverage threshold of 10%, a 98% ANI cutoff would be valid to retain genome diversity in the current study, balancing redundancy removal and mis-mapping issues.

Line 384-385 modified to: "Although stringent, an ANI value of 98% was selected to balance genome diversity, while minimizing cross-mapping issues and avoiding redundancy > 98% (Delmont et al. 2018).

Small typo: paired-en reads -> paired-end reads

> *BigMAP Analysis (Lines 427–430): Bowtie is not suitable for long-read data due to its error profiles and read length limitations; it may bias RPKM/TPM normalization. A long-read-aware alignment and normalization to housekeeping genes would yield more reliable abundance estimates.*
> *Differences in RNA depletion across samples further complicate comparisons.*

<u>Author reply</u>: We thank the reviewer for this insightful and constructive comment. We fully agree that Bowtie2 is not optimal for long-read data due to its sensitivity to error profiles and read length limitations. As Bowtie2 is the default aligner in the BiG-MAP pipeline, we initially used it to evaluate the performance of the standard workflow on our dataset. Following the reviewer's valuable suggestion, we have now re-mapped all metatranscriptomic reads using Minimap2 (v2.26, r1175) with the -x map-ont preset to ensure more accurate alignment of Nanopore reads. To implement this improvement, we removed the default setting of Bowtie2 in the BiG-MAP.map code, replaced it with Minimap2 and adjusted the TPM normalisation accordingly. This re-analysis resulted in quantitative differences in gene expression estimates but did not alter the overall expression trends or main conclusions of the study.
The revised results, including updated figures and tables, have been incorporated throughout the manuscript. The specific changes resulting from this updated mapping approach are summarised below.

Results section (lines 165–166, 169–171, 174, 176, 182, 185, 198-200, 203-205): Updated expression values and corresponding descriptions to reflect the re-analysis using Minimap2.
Results section (line 183): Added the sentence *"Similarly, the Other class showed significantly higher expression compared to NRP (p = 0.0127) and Polyketide (p = 0.0179)."* to reflect the updated analysis.
Results section (lines 183–186): Deleted this section because the results changed following re-analysis with Minimap2.
Results section (line 192): Added six additional phyla to match the updated results from the revised analysis.
Discussion (lines 299, 301–304, 307, 311–312, 315, 320–323, 327–329): Updated taxonomic information to reflect the new classification and revised transcriptional level descriptions based on the updated mapping results.
Methods section (lines 426–443): Updated the description of the mapping method to reflect the modification of the BiG-MAP pipeline from the default Bowtie2 aligner to Minimap2 and revised the corresponding references accordingly.

Looks good to me. One suggestion would be to open an issue or feature request on the BiG-MAP GitHub page to recommend this update, or, if possible, to submit a pull request with the developed code so that the tool's developers can integrate this improvement into the main branch for future use.

Additional Comments

Abstract (Line 20): "Antimicrobial potential" is mentioned but never discussed or supported in the manuscript.

<u>Author reply</u>: We thank the reviewer for pointing this out. We have revised the Discussion to explicitly address the antimicrobial potential of AMOR BGCs. In the revised text, we added a section (lines 309-315) describing the antimicrobial peptides detected and actively expressed in

the AMOR biofilms, including well-characterized groups such as thiopeptides and sactipeptides. These examples strengthen and clarify our discussion of the antimicrobial potential encoded by AMOR BGCs.

As far as I can tell, the newly added comparison is made to thiopeptide and sactipeptide BGCs reported in the literature in general, rather than to direct, one-to-one sequence similarity between the BGCs identified in this work and previously characterized clusters from literature. As this study does not include bioactivity activity assays, the current wording in the abstract ("show strong antimicrobial potential") may be misleading. I therefore recommend softening or rephrasing this statement to better reflect the scope of the presented evidence.

> *Results (Lines 202–215): Not all reported metabolites are necessarily antimicrobial; the wording should be refined.*

Author reply: We have revised the wording in this section to clarify that not all detected BGCs necessarily produce antimicrobial metabolites, as their functions can vary depending on environmental conditions, regulatory dynamics, and expression levels. Our intention is to highlight that hydrothermal biofilm BGCs represent a promising source for future exploration of antimicrobial compound diversity, rather than implying that all BGCs identified are antimicrobial in nature.

The refined wording still appears to imply that all identified BGCs have antimicrobial properties. For example, the statement "Hydrothermal biofilms harbored a diverse array of antimicrobial BGCs, many of which were actively transcribed" suggests a bioactivity/function that is not directly supported for all BGCs. While the authors provide a helpful and nuanced discussion a few lines later, it would be preferable to state explicitly from the outset that BGCs encode a wide range of compounds, of which only a subset may be bioactive, including antimicrobials. The authors could then note that RiPPs are more frequently associated with bioactivity, while still acknowledging that this does not exclude alternative or currently unknown functions. Adding words like "potential" to the claims will already help softening it.

In addition, it is important to note that BGCs themselves are not antimicrobial, rather, the compounds potentially derived from these clusters may have antimicrobial activity. Similar wording appears in several places throughout the manuscript and would benefit from refinement to more accurately reflect this distinction.
