# Supplementary material for: Diverse biosynthetic pathways in Arctic hydrothermal biofilms
Source: Nat Commun. 2026 Sep 3;17:10469. doi: 10.1038/s41467-026-77384-5 (PMC13635244; doi:10.1038/s41467-026-77384-5)
Supplement: Supplementary file 3 [file 41467_2026_77384_MOESM1_ESM.pdf]

Supplementary Figures

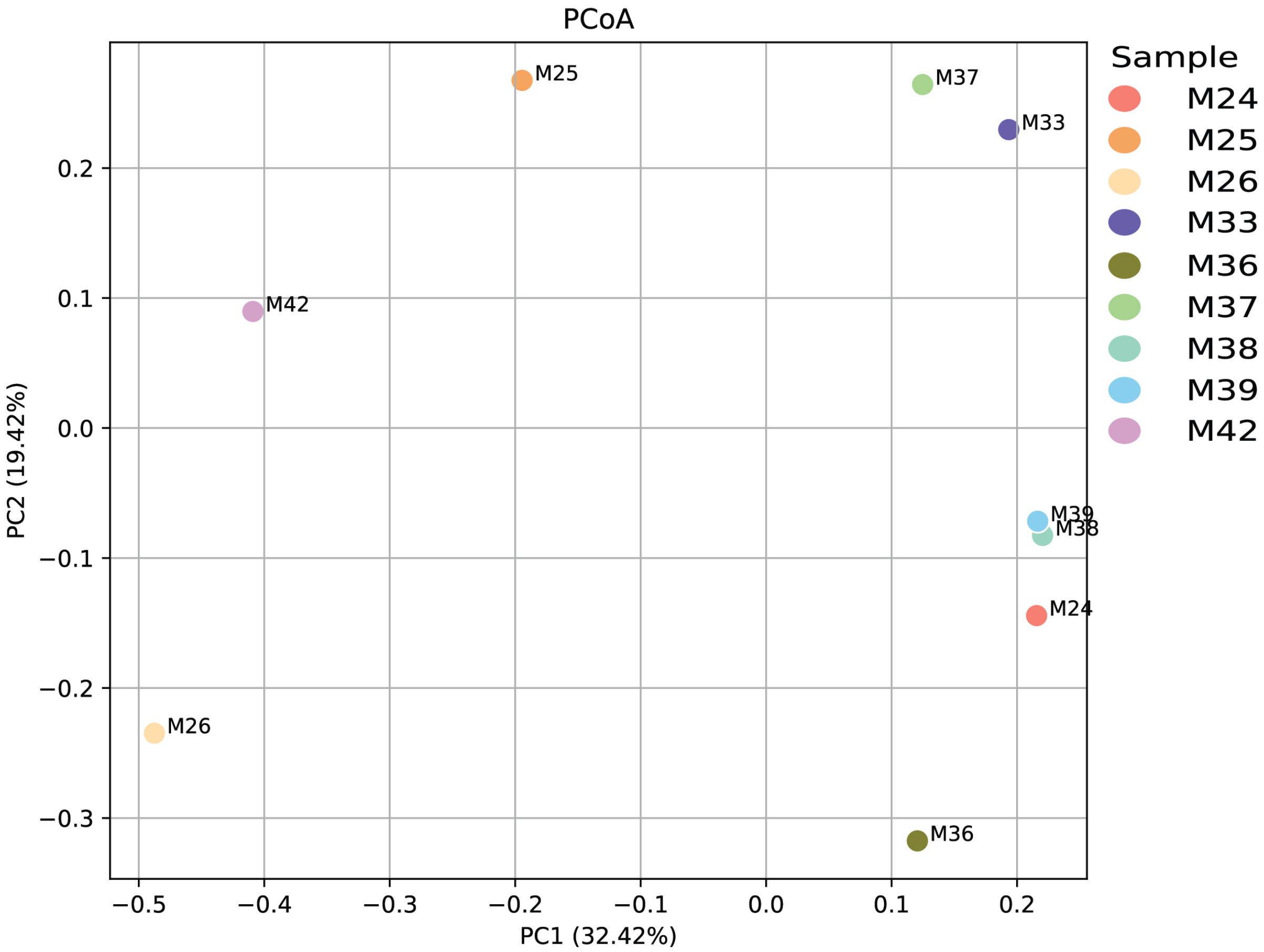

Supplementary Fig. 1: **Variation in microbial community composition across AMOR hydrothermal vent biofilms.** Principal Component Analysis (PCA) of microbial communities from AMOR deep-sea hydrothermal vent biofilms. PCA based on Bray-Curtis dissimilarity of MAGs relative abundances at the order level, with confidence interval at 95% and 1,000 bootstrap iterations.

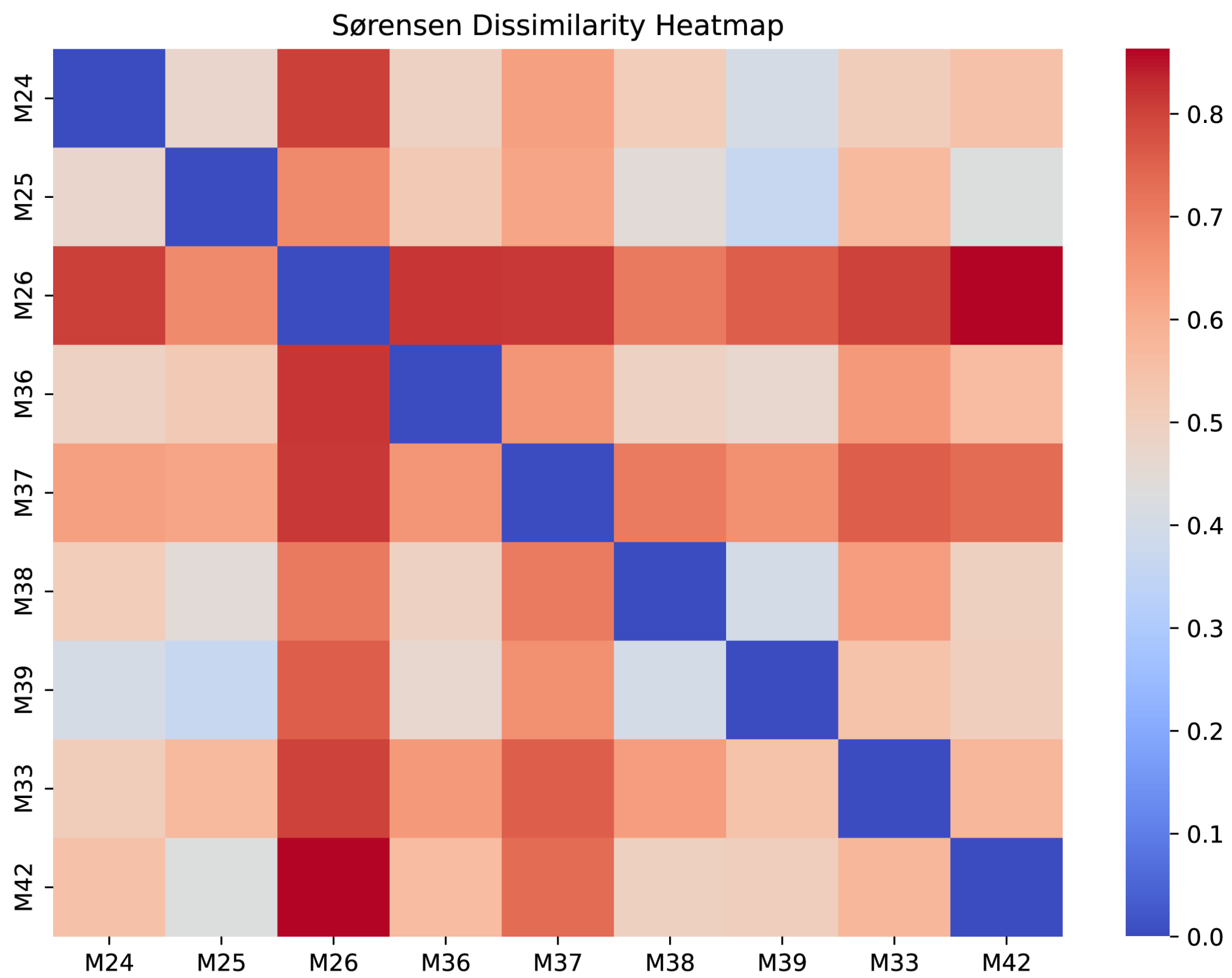

Supplementary Fig. 2: Taxonomic turnover among AMOR hydrothermal vent biofilms. Differences in microbial taxonomic composition among AMOR biofilms. Heatmap based on Sørensen dissimilarity calculated from the presence/absence of microbial orders across nine biofilms. The significance was assessed using a permutation test with 1,000 iterations.

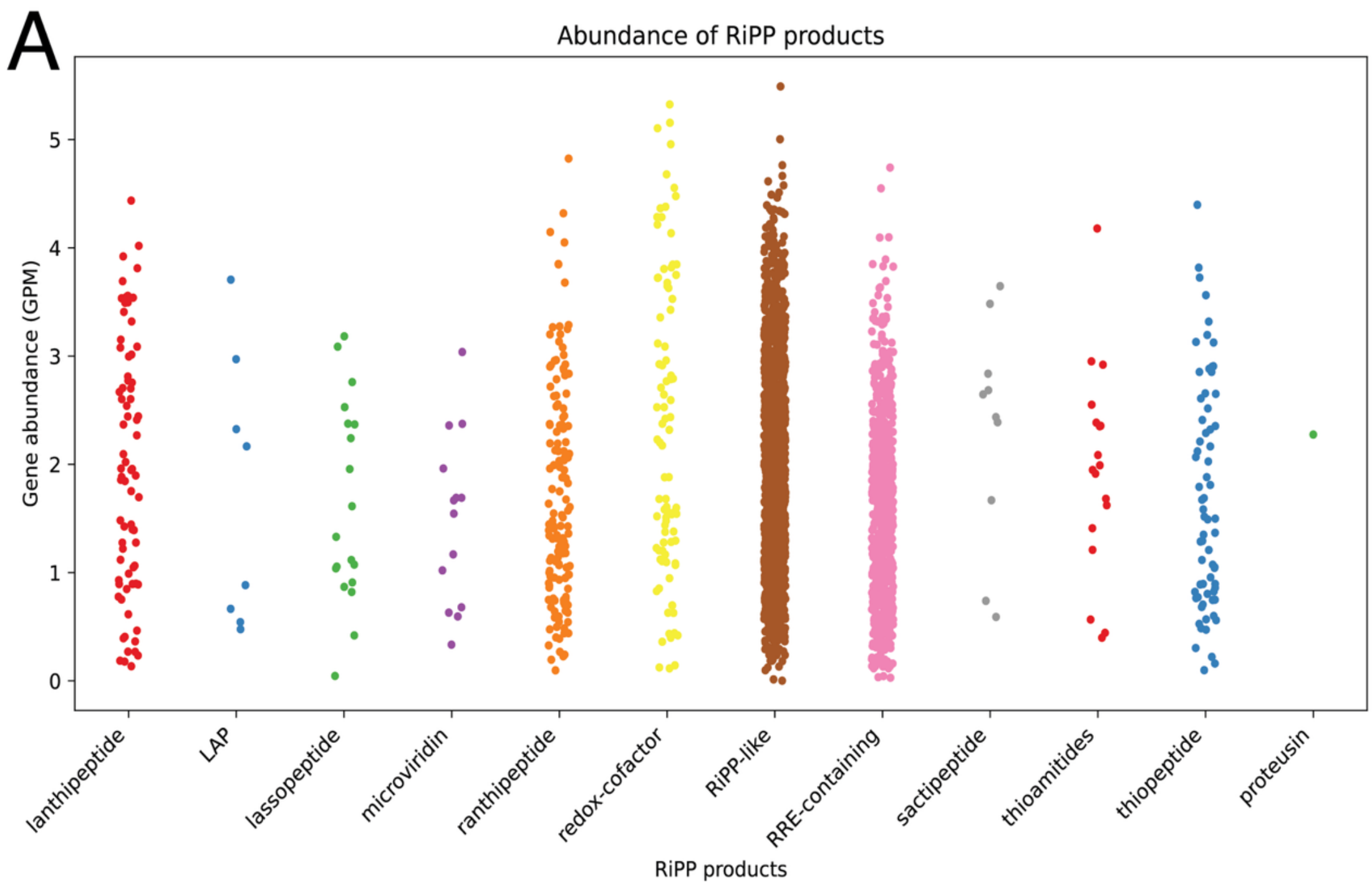

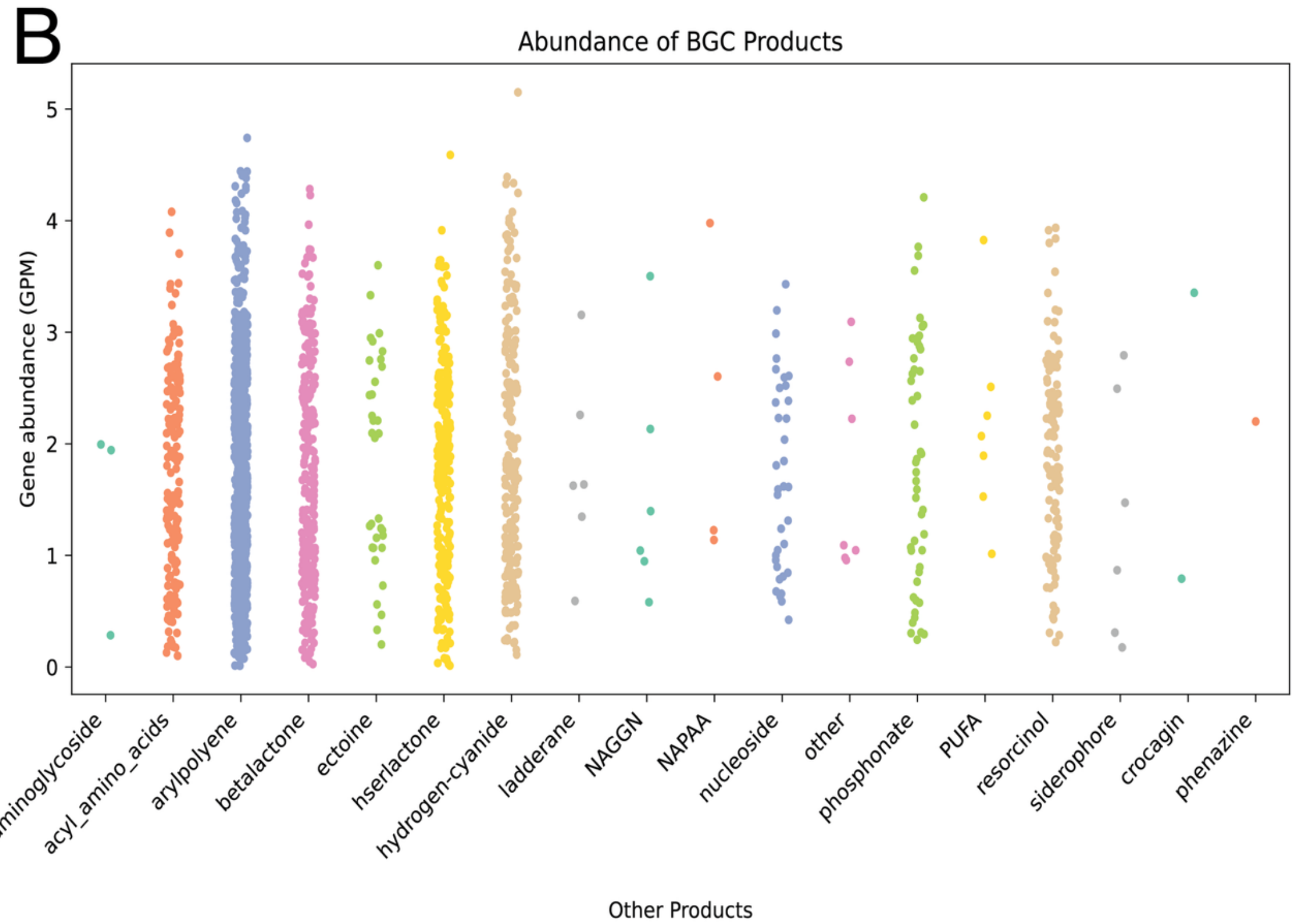

Supplementary Fig. 3: Distribution of RiPP and other BGC cluster classes across AMOR biofilms. Relative abundance of different BGC classes in AMOR biofilms showing (A) RiPPs and (B) Other. Abundance is measured in GPM, as estimated using BiG-

MAP.map, which maps predicted BGCs back to their corresponding metagenomes. Each data point represents an individual BGC.

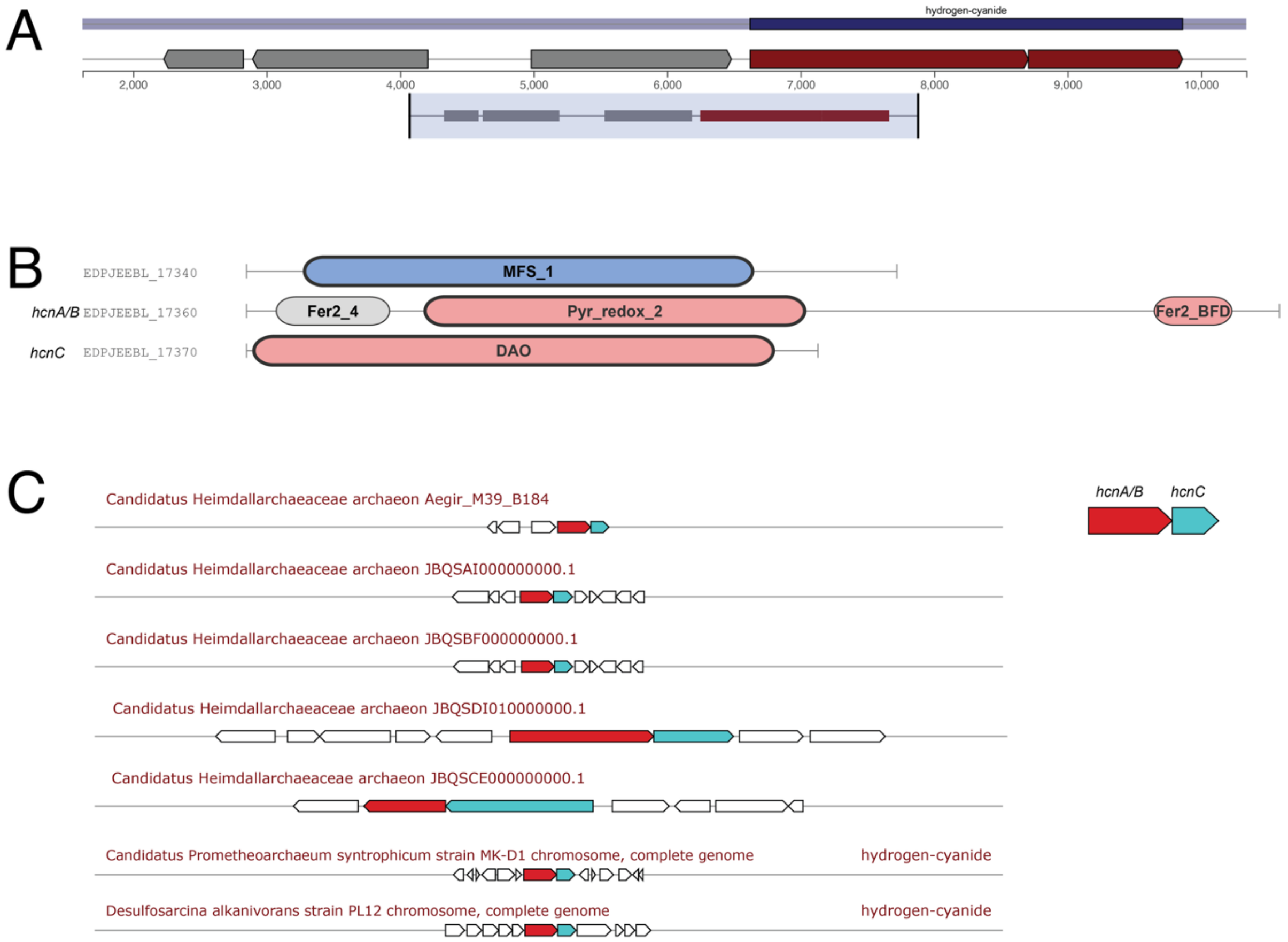

Supplementary Fig. 4: Putative hydrogen cyanide synthase BGC encoded by a Heimdallarchaea MAG. Hydrogen cyanide synthase (HCN) BGC in the archaeal MAG Aegir_M39_B184 (phylum *Asgardarchaeota*). (A) Coding region of the core biosynthetic genes, shown in comparison to the reference cluster, with pHMM detection rules applied. (B) Identified Pfam domains within the HCN BGC of the Heimdallarchaeion Aegir_M39_B184. (C) ClusterBlast results showing hits to similar known clusters from the antiSMASH database, including high-scoring blast hits towards other *Heimdallarchaeia*, as predicted by antiSMASH v.7.0.

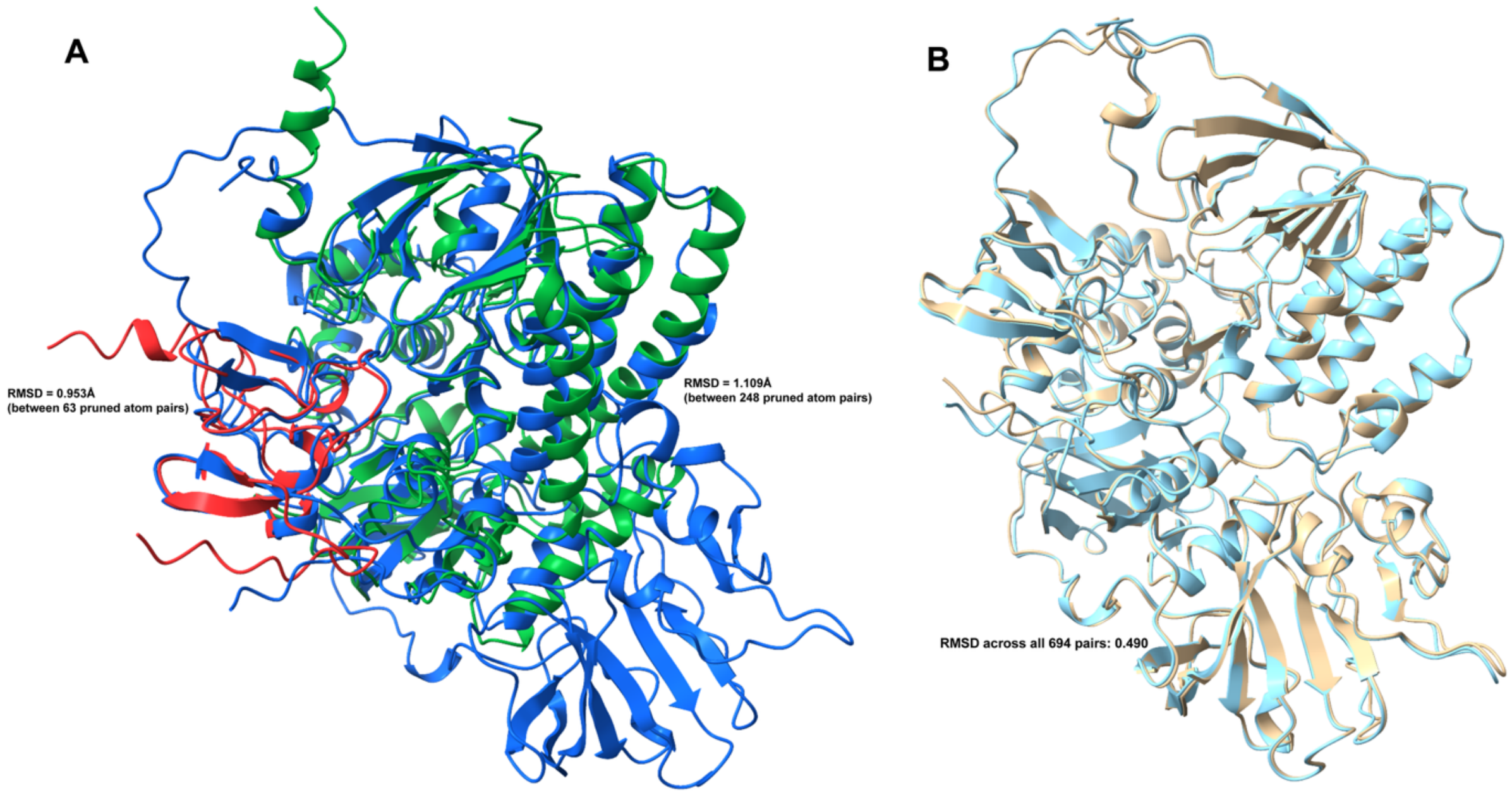

Supplementary Fig. 5: **Structural similarity of archaeal and bacterial hydrogen cyanide synthase proteins.** Structural comparison of *hcn* proteins showing **A**; a superimposed structural model of the proposed *hcnA*B from the *Candidatus* Heimdallarchaeaceae archaeon Aegir_M39_B184 (blue) together with the structural models of *hcn*A (GenBank VVN46760.1:red) and *hcn*B (GenBank VVN46770.1:green) from *Pseudomonas fluorescens* (GenBank CABVHD010000039.1), and **B**; a superimposed structural model of the proposed *hcn*AB from the *Candidatus* Heimdallarchaeaceae archaeon Aegir_M39_B184 (cyan) and the model of a FAD-dependent oxidoreductase identified as part of HCN in antiSMASH v.7.0 in a *Candidatus* Heimdallarchaeaceae archaeon from Guayamas Basin (light brown; MHA1303415.1). Structure predictions were generated using the AlphaFold Server[65] and are provided subject to the AlphaFold Server Output Terms of Use (https://alphafoldserver.com/output-terms) and visualized using ChimeraX v.1.11[117–119] (https://www.rbvi.ucsf.edu/chimerax). Note: The AlphaFold-derived image remains subject to those Terms and is excluded from any broader CC-BY reuse rights applicable to this article.

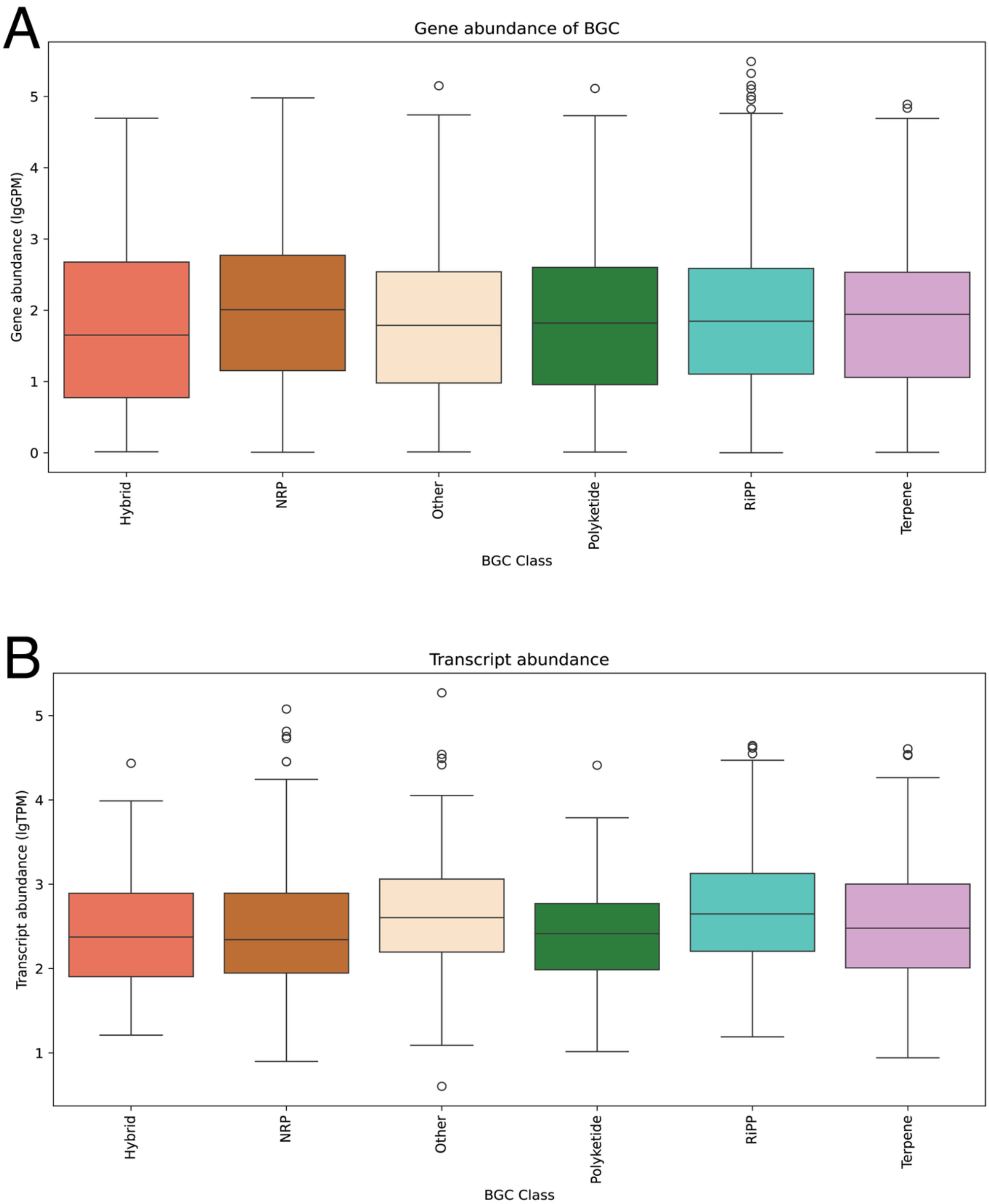

Supplementary Fig. 6: **Metagenomic abundance a metatranscriptomic expression of BGC classes.** Abundance and expression of BGC classes across AMOR biofilms showing (A) Gene abundance of BGC classes, expressed as GPM, and (B) Transcript abundance of BGC classes, expressed as TPM. Abundance was estimated using BiG-MAP. Box plots show the interquartile range (IQR), with whiskers extending to 1.5 IQR.

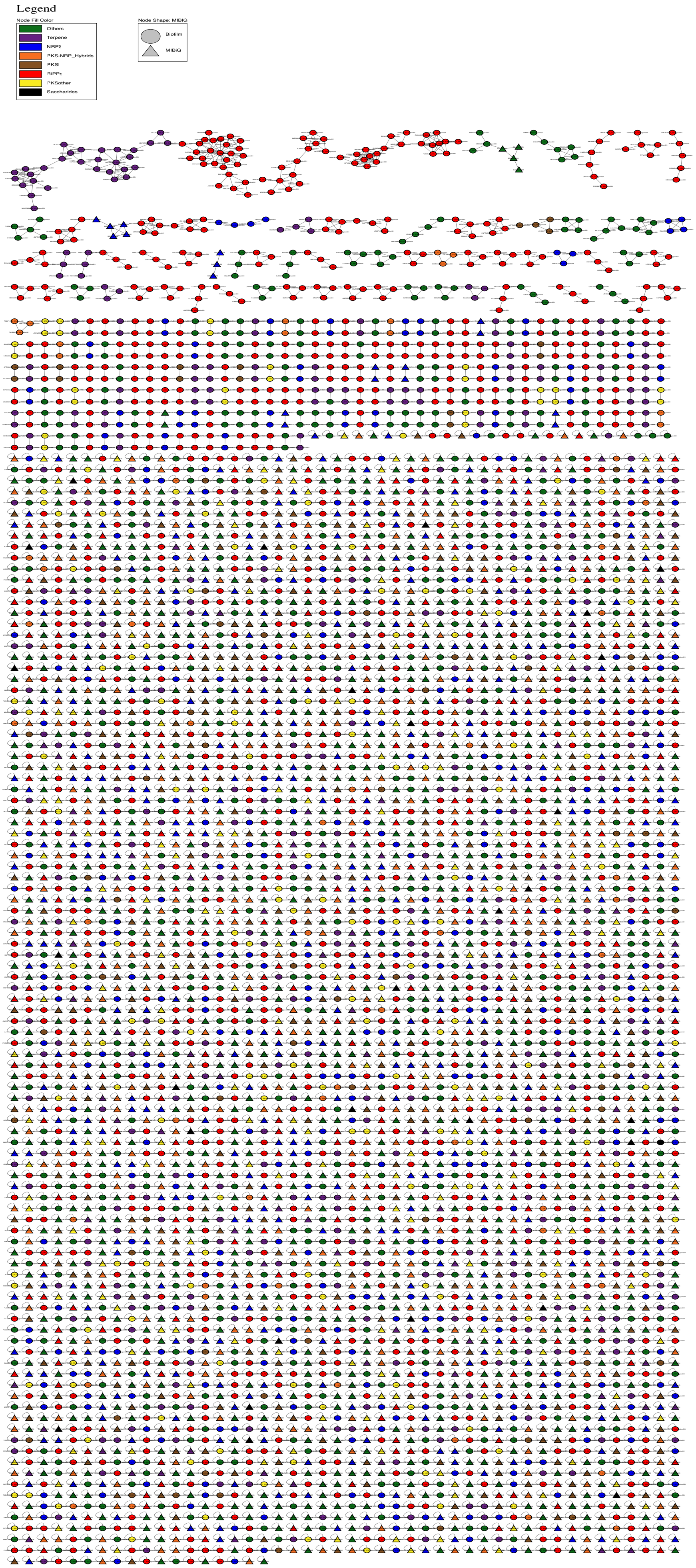
Legend
Node Fill Color
Others
Terpene
NRPS
PKS-NRP_Hybrids
PKS
RiPPs
PKSother
Saccharides
Node Shape: MIBIG
Biofilm
MIBiG

Supplementary Fig. 7: BGC similarity network of AMOR biofilms. Cluster similarity network of BGCs from AMOR biofilms compared with the MIBiG 3.1 database, generated using BiG-SCAPE v1.1.5 (similarity cutoff = 0.3). Nodes represent BGCs and are coloured by BiG-SCAPE class. Circular nodes denote AMOR biofilm BGCs, while triangular nodes represent MIBiG reference clusters. All singletons are shown.
