# Supplementary material for: Diverse biosynthetic pathways in Arctic hydrothermal biofilms
Source: Nat Commun. 2026 Sep 3;17:10469. doi: 10.1038/s41467-026-77384-5 (PMC13635244; doi:10.1038/s41467-026-77384-5)
Supplement: Supplementary file 4 [file 41467_2026_77384_MOESM2_ESM.pdf]

Description of Additional Supplementary Files

File Name: Supplementary Data 1
Description: DNA sequencing and metagenome statistics of bioilms from the Arctic Mid-Ocean Ridges. Metagenome IDs corresponds to samples in main Table 1.

File Name: Supplementary Data 2
Description: Nanopore long-read sequencing of RNA exctracted from hydrothermal bioilms. Metatranscriptome IDs corresponds to samples in main Table 1.

File Name: Supplementary Data 3
Description: Database of non-redundant MAGs (n=1140).

File Name: Supplementary Data 4
Description: BGCs predicted from 9 AMOR biofilms (n=2965).

File Name: Supplementary Data 5
Description: Expression profiles of BGCs across seven biofilms from Arctic deep-sea hydrothermal vents. The table includes normalized transcript abundance for each BGC, along with associated metadata such as BGC class, predicted product, and taxonomic affiliation.

File Name: Supplementary Data 6
Description: Gene Cluster Families (GCFs) formed from biosynthetic gene clusters (BGCs) identified in Arctic hydrothermal vent biofilms and from the MIBiG 3.1 database. Clustering was performed using BiG-SCAPE v1.1.5 with a similarity cut-off of 0.3.

File Name: Supplementary Data 7
Description: Statistical results from Kruskal–Wallis analyses and subsequent Dunn's pairwise post hoc tests. BGC classes included: ['Hybrid', 'NRP', 'Other', 'Polyketide', 'RiPP', 'Terpene'].

File Name: Supplementary Data 8
Description: Top 50 Blastp hits of the identified HcnAB in the Candidatus Heimdallarchaeaceae archaeon MAG Aegir_M39_B184.
